# Enhancing Enzyme Activity With Mutation Combinations Guided by Few‐Shot Learning and Causal Inference

**DOI:** 10.1002/anie.7768514

**Published:** 2026-06-22

**Authors:** Lin Guo, Xiaoguang Yan, Yali Lu, Shengxin Nie, Mingyue Ge, Yukun Li, Weiguo Li, Xiaochun Zhang, Dongmei Liang, Yihan Zhao, Hongxiao Tan, Xiling Chen, Shilong Fan, Yefeng Tang, Jianjun Qiao, Boxue Tian

**Affiliations:** ^1^ MOE Key Laboratory of Bioinformatics State Key Laboratory of Molecular Oncology Beijing Frontier Research Center for Biological Structure School of Pharmaceutical Sciences Tsinghua University Beijing China; ^2^ State Key Laboratory of Synthetic Biology Key Laboratory of Systems Bioengineering (Ministry of Education) School of Synthetic Biology and Biomanufacturing Tianjin University Tianjin China; ^3^ Zhejiang Institute of Tianjin University Shaoxing China; ^4^ College of Life Sciences Inner Mongolia University Hohhot China; ^5^ Beijing Advanced Innovation Center for Structural Biology School of Life Sciences Tsinghua University Beijing China

**Keywords:** causal inference, mutagenesis, physics‐inspired few‐shot learning, protein design, protein language models

## Abstract

Designing enzyme sequences to enhance product yield represents a fundamental challenge in metabolic engineering. Here, we established a workflow that integrates computational predictions with efficient experimental iteration to obtain outsized gains in product yield. Based on causal inference and examination of published datasets, we realized and ultimately experimentally confirmed that in vivo unit yield (yield/expression) can serve as an attractive surrogate for aqueous *k_cat_*/*K_m_* when optimizing for activity. In our workflow, we initially predict activity‐enhancing single mutants by calculating the binding affinities of reactive intermediates, followed by experimental investigations of unit yield. Subsequently, we predict activity‐enhancing mutation combinations using a few‐shot learning model we developed called Physics‐Inspired Feature Selection of Protein Language Models (PIFS‐PLM), which requires only 60–100 experimentally examined mutation combinations as input. In a case study of a bicyclogermacrene (BCG) synthase, we achieve a 73‐fold increase in BCG yield or a 15% increase in BCG selectivity based on combinations of 12 individual mutations, and provide extensive crystallographic and biochemical evidence for impacts from specific mutations. Thus, optimizing for unit yield is highly efficient as an alternative to optimizing for thermostability, and our study provides a powerful workflow for the efficient engineering of high‐yield enzyme variants.

## Graphical abstract

We present an integrated workflow that predicts activity‐enhancing mutation combinations from minimal experimental data. By proposing in vivo unit yield (yield/expression) as a surrogate for *k_cat_*/*K_m_* through causal inference, and visualizing local activity landscape, it effectively guides product yield improvement.

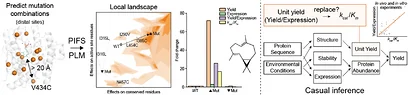

## 1 Introduction

Designing enzyme sequences to boost chemical product yield is a fundamental challenge in metabolic engineering [1]. Mutations affecting enzyme function can be roughly categorized into two types: Those influencing catalytic activity and selectivity (typically for active site residues), and those that influence protein abundance [2, 3, 4, 5, 6, 7]. While active site mutations can directly influence catalytic activity, functionally relevant mutations at positions outside the active site are prevalent in proteins, contributing, for example, to protein stability, protein‐protein interactions, coenzyme binding, and conformational changes [8]. Early computational efforts toward enzyme sequence design have used physics‐based or statistical‐based energy functions to guide enzyme activity optimization [9]. Most of these methods are based on either activation energy or protein thermostability.

An established general strategy for enhancing enzymatic activity is optimizing protein thermostability [10]. Many enzyme design methods rely on this strategy, and a commonly used design method is to constrain a catalytic region and then focus design efforts on other positions [11]. A profound shift in methodology has recently occurred with the advent of machine learning (ML) methods [7, 12, 13, 14, 15, 16, 17, 18]. A surge in protein language models (PLMs) has opened new avenues for protein sequence design [19, 20, 21]; these models learn physical and evolutionary attributes of proteins from training data. Unsupervised learning with PLMs can be employed to predict functional sites, and to guide optimization toward specific applied goals in protein engineering generally (i.e., including beyond enzyme engineering) [22, 23]. For example, a structure‐informed protein language model can effectively guide the unsupervised evolution of protein complexes, achieving significant improvements in the performance of therapeutic antibodies against SARS‐CoV‐2 variants [23]. Supervised learning with PLMs requires experimental data for training and prediction. Previous studies have demonstrated the effectiveness of supervised fine‐tuning of PLMs in protein engineering [24, 25]. For example, a machine learning approach that efficiently constructs virtual fitness landscapes, enabling screening of millions of protein sequences and achieving performance comparable to traditional high‐throughput methods [25].

Combining activity‐enhancing single mutants can manifest in outsized gains for product yield improvement [26, 27]. Despite the progress achieved through directed evolution and AI‐aided mutation prediction, predicting mutation combinations remains a challenge [28]. Protein expression must be considered in metabolic engineering, as it directly influences product yield [5]. We are intrigued that directed evolution efforts and AI methods based on thermostability optimizations cannot differentiate between activity‐enhancing and expression‐enhancing mutants [29]. Experimentally verifying the *k_cat_*/*K_m_* (the gold standard of enzyme activity) for single mutants and mutation combinations is a tedious and resource‐intensive process. Further, when the mutational space is vast and available training data is scarce, it becomes difficult to train a generalizable model to predict mutation combinations likely to enhance activity [28].

Here, we established a workflow that integrates computational predictions with efficient experimental iteration. By using causal inference and examining datasets from published reports [27, 30, 31, 32, 33, 34, 35, 36] of successful yield‐boosting engineering efforts, we realized that in vivo unit yield (yield/expression) may be suitable as a rough‐and‐ready replacement for *k_cat_*/*K_m_* in metabolic engineering efforts. In our workflow, we first predict single mutation by evaluating the binding affinities of reactive intermediates, and experimentally validate that the single mutants do increase activity. To further boost the product yield of enzymes via mutation combinations, we integrate physical and evolutionary knowledge with PLMs, and have developed a physics‐inspired feature selection (PIFS) model that estimates the mutational effects on catalytic efficiency and protein abundance. Specifically, we evaluate mutational effects on the active site residues and conserved residues, and our PIFS‐PLM model provides insight into the local activity landscape of the enzyme. In a case study of a bicyclogermacrene (BCG) synthase, we achieved a 73‐fold increase in BCG yield. The same workflow also delivered a 15% increase in BCG selectivity, through combinations of 12 individual mutations. We subsequently employed crystallography, thermostability/expression measurements, kinetics measurements, and molecular dynamics simulations to evaluate the impacts of the selected mutations on biosynthesis. Our study supports that optimizing for expression and for unit yield is highly efficient as an alternative to optimizing for thermostability and provides a powerful tool for the efficient engineering of high‐yield enzyme variants harboring multiple mutations.

## 2 Results and Discussion

### 2.1 Workflow for the Identification of Activity‐Enhancing Mutation Combinations

**FIGURE 1:**
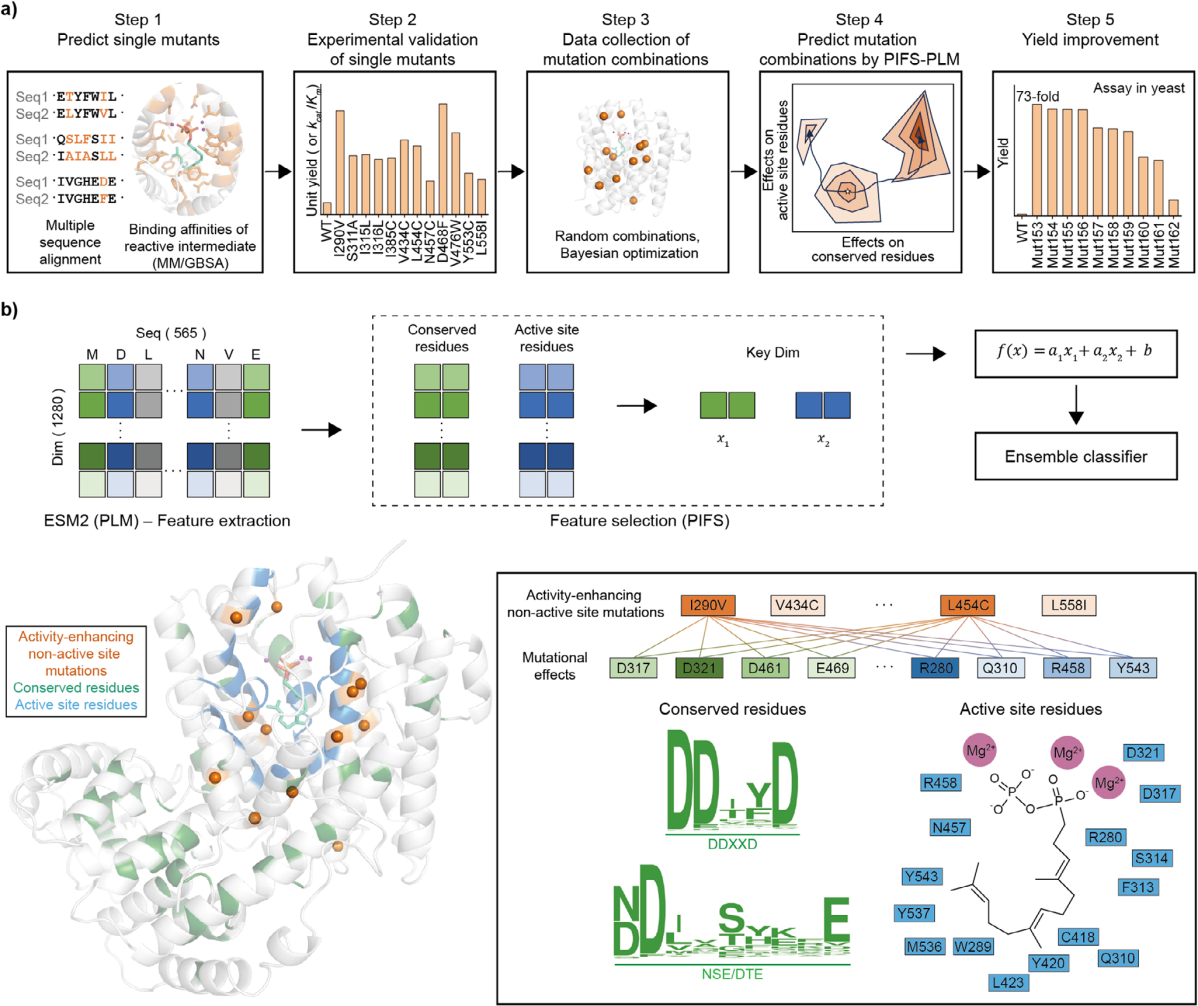
Workflow for identifying activity‐enhancing mutation combinations. (a) Integration of computational predictions and experimental validation. After screening single mutants, combinatorial mutants were predicted. (b) Schematic of the PIFS‐PLM method, including feature selection from functionally relevant active site or conserved residues and ensemble classification (see Methods section for details).

To enhance product yield through mutations of the wild type (WT) enzyme, combining verified activity‐related mutations is generally more efficient than targeting other mutation sites. Using the unit yield definition, we can verify activity‐enhancing mutations in vivo. We have established a workflow that integrates computational predictions with experimental iteration (Figure 1a). First, single mutation sites are predicted by using multiple sequence alignment (MSA) or by computing the molecular mechanics with generalized born and surface area solvation (MM/GBSA) binding affinities of the reactive intermediate for all possible single mutants. The MM/GBSA approach has been reported previously in enzyme function prediction (but not for activity enhancement), under the assumption that enzymes inherently bind transition states and intermediates [37, 38, 39]. We then validate the unit yield of yield‐enhancing single mutants both in vitro and in vivo to exclude mutants that only enhance expression without improving activity.

We then gather in vivo experimental data for a subset of combinations of the aforementioned single mutants (60‐100 mutation combinations), followed by a Bayesian optimization step to generate an initial training dataset (20–30 mutation combinations, methods). Bayesian optimization is a method for identifying input parameters that maximize or minimize an objective function by constructing a model, typically a Gaussian process, and iteratively updating it to approximate the global optimum. The PIFS‐PLM model (Figure 1b) is then used to predict optimal mutation combinations, which are expressed in cells and evaluated for product yield.

PIFS‐PLM contains three steps (Figure 1b). In the first step, we identify a subset of functionally relevant residues based on active site locations and sequence conservation, and then analyze their PLM feature changes in terms of each mutation combination (Figures 1b and S1a–c). We use the widely adopted PLM ESM2 (650 M parameters) [20] for feature extraction, selecting features based on physicochemical information about the enzymes. ESM2 features consist of a dimensionality of 1280 multiplied by the number of residues. Of note, experimentally verified non‐active site residues that enhance activity (Figure 1b in orange) are excluded from this subset of active site and conserved residues. The dimension exhibiting the largest response to our experimental data—that is, the PLM feature that is, apparently most sensitive to mutation effects—is used for building PIFS‐PLM model (Figure S1d–h). Conserved sites are generally associated with overall protein structural stability, and mutations at other positions that perturb these conserved residues may consequently influence protein stability and protein abundance. Although residues in the active site pocket may also contribute to stability or protein abundance, we assume their greater contribution lies in catalysis. Non‐active site residues contribute to catalysis by propagating physical interactions with active site residues [40], as well as by affecting protein abundance [5, 6]. Accordingly, we categorize mutational effects into two major types of residues: Active site residues and conserved residues.

In the second step, we assess the predicted magnitude of changes resulting from mutation of the pre‐selected conserved and active site residues within the chosen dimension. This provides insights into how specific mutations are likely to influence the enzyme activity. Separate values are calculated for each mutant based on their conserved (*x*_1_) and active site (*x*_2_) residue characteristics. This design effectively transforms the feature space into a linearly separable space while reducing the parameter count, enabling the model to capture higher‐order epistasis through feature aggregation. In the final step, classification models are trained using conserved and active site residue changes (relative to WT) as variables, aiming to predict yield.

### 2.2 Design and Characterization of Activity‐Enhanced Single Mutants

**FIGURE 2:**
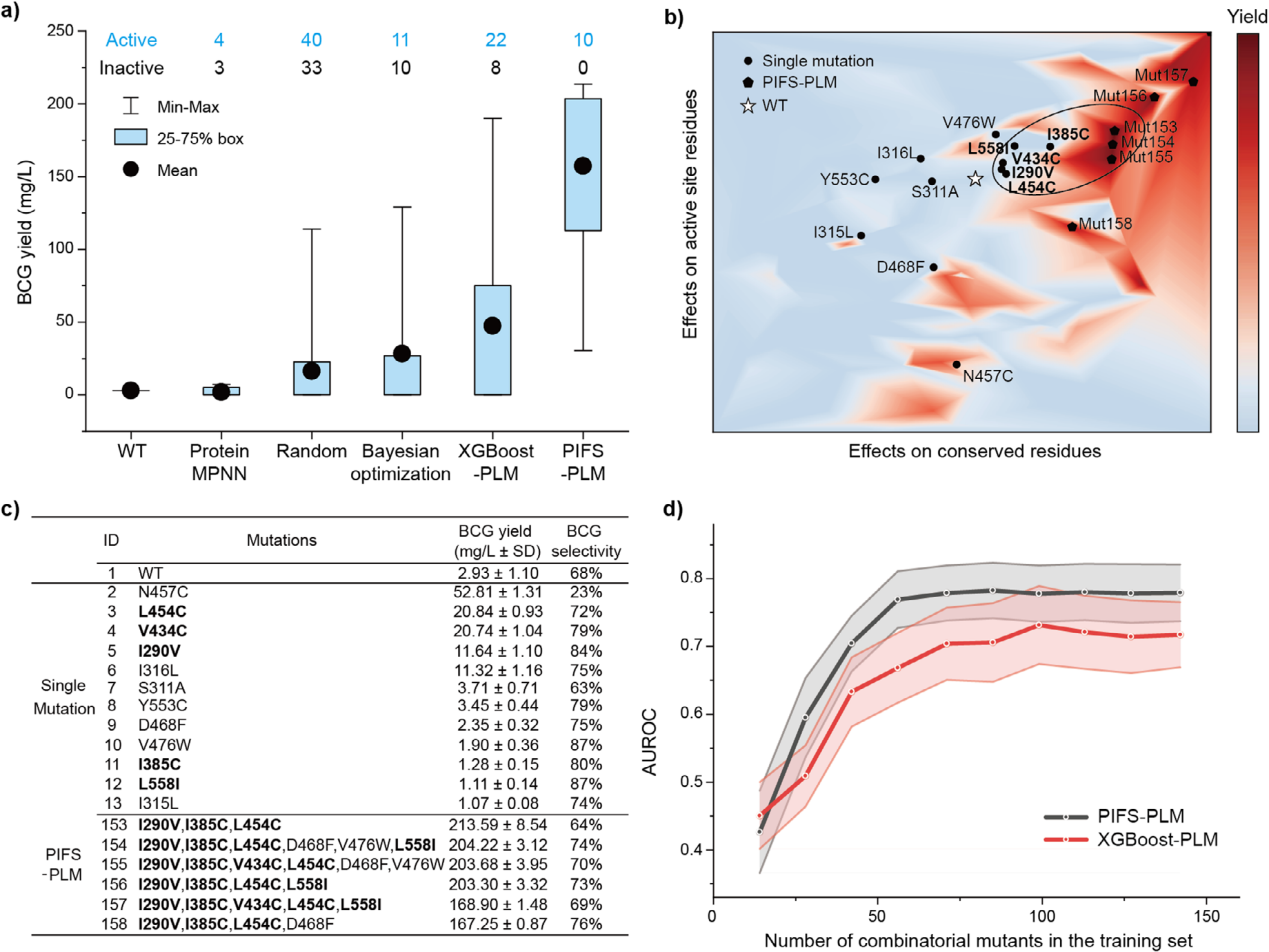
Optimization of LdTPS5 through PIFS‐PLM‐guided mutation combinations. (a) Boxplots of BCG yields in yeast for mutants obtained through ProteinMPNN (direct prediction without filtering), random screening, Bayesian optimization, XGBoost‐PLM, and PIFS‐PLM; the number of inactive mutants is indicated above each box. (b), (c) Visualization of the local activity landscape explored by PIFS‐PLM (b), with a table presenting additional BCG yield data for activity‐enhancing single and high‐yield combinatorial mutants (c). (d) Evaluation of PIFS and XGBoost prediction accuracy with increasing number of combinatorial mutants in the training set, as measured by AUROC.

Taking a BCG synthase as an example for yield enhancement (Figure 2a), this enzyme catalyzes the conversion of farnesyl diphosphate (FPP) to BCG. BCG and its derivatives possess intriguing bioactivities [41]; for example, partheniol, guayulin A, and euglobal‐III exhibit fungistatic, allergenic, and cytotoxic properties, respectively (Figure S2). Testing a total of three enzymes from three species in using a previously reported yeast strain with high FPP yield showed that LdTPS5 from *Lippia dulcis* had the highest BCG yield in yeast (2.93 mg/L, Tables S1 and S2) [42, 43, 44].

We evaluated potential single mutation sites based on the MM/GBSA predicted binding affinities for the product precursor carbocation intermediate for all possible single mutants (Figure S3a), containing 6213 (327 × 19) potential mutations. Notably, most (5141, 82.74%) of the mutations were predicted as deleterious, as indicated by positive binding affinities (relative to LdTPS5^WT^) (Figure S3b). Subsequently, we experimentally tested 90 predicted beneficial single mutants distributed across eight residues: 26 single mutants exhibited either higher yield or improved activity compared to the LdTPS5^WT^ (Figure S3c and Table S3).

In addition to the MM/GBSA prediction, we employed the commonly used rational design approach MSA [45] to identify potential single mutants for LdTPS5. This analysis predicted nine putative sites for yield‐improving mutations. (Figure S3d and Table S3). Subsequent experimental validation in yeast confirmed that, six single mutants substantially increased enzyme activity, including I290V and I315L, which were also predicted as yield‐enhancing by MM/GBSA. Finally, experimental determination of *k_cat_*/*K_m_* values for the 12 single mutants (I290V, S311A, I315L, I316L, I385C, V434C, L454C, N457C, D468F, V476W, Y553C, and L558I) confirmed that each showed improved activity (2.85‐ to 9.32‐fold) over the LdTPS5^WT^, with the D468F, I290V, and V434C mutants providing the greatest enhancements (9.32‐, 8.76‐, and 6.31‐fold, respectively; Figure S3e). Although these improvements observed in the single mutants were less than an order of magnitude, as is commonly reported for single mutants in enzyme engineering, we hypothesized that utilizing these variants as modular building blocks for combinatorial mutagenesis could further enhance their additive effects. Experimental validation of these combined mutants supported this synergistic effect of multiple weakly beneficial mutations. Notably, selection of the initial candidate mutations through MM/GBSA or MSA serves only as a screening tool, independent of the feature selection process for PIFS‐PLM training.

### 2.3 Predicting Activity‐Enhancing Mutation Combinations With PIFS‐PLM

We then randomly combined these 12 single mutations and conducted Bayesian optimization to generate an initial dataset for training a PIFS‐PLM model. Subsequent assays for all 10 mutant combinations predicted by PIFS‐PLM are active, with nine of them (LdTPS5^Mut153‐161^) producing BCG at levels exceeding 100 mg/L. Among them, LdTPS5^Mut153^ (I290V/I385C/L454C) exhibited the highest BCG yield (213.59 mg/L). Another mutant, LdTPS5^Mut154^ (I290V/I385C/L454C/D468F/V476 W/L558I), achieved a yield of 204.22 mg/L with an improved selectivity (Figure 2b,c). These results showcase the capacity of the PIFS‐PLM model to predict elite‐performing mutant combinations.

To directly assess the extent to which combinatorial mutants enhanced catalytic efficiency, we purified several mutants and measured their *k_cat_*/*K_m_* values. We found that the top‐performing combinatorial mutants, LdTPS5^Mut143^ and LdTPS5^Mut153^, exhibited 21.27‐ and 16.69‐fold higher *k_cat_*/*K_m_* compared to LdTPS5^WT^, respectively (summarized in Table S4). These results provide direct kinetic evidence that the mutation combinations confer synergistic improvements in the intrinsic catalytic efficiency of LdTPS5 beyond the additive effects of individual mutants.

The PIFS‐PLM model allows us to generate a “local landscape of enzyme activity” that can provide an intuitive understanding of the yield enhancement process (Figures 2b and S4). We decompose the effects of mutations into two dimensions to combine mutations with consistent directions; this decomposition approach is based on activity and effects other than activity (e.g., expression and stability), which are in general respectively related to active site and conserved residues.

For the initial stage with random combinations of single mutations, the local landscape map presents scattered, isolated peaks, indicating the (intended) lack of directionality in the search (Figure S4a). After the Bayesian optimization from experimental data, the local landscape maps present broader peaks that are more connected (Figure S4b). Comparison of local landscape maps suggested that the PIFS‐PLM ventured into areas that were not predicted as high‐yield by Bayesian optimization (Figure S4d). Thus, the local landscape maps can identify potential high‐activity areas to target for further mutagenesis efforts, while also identifying isolated, low‐activity points with apparently minimal potential. These results suggest that local maps from our PIFS‐PLM model can identify enzyme regions that are likely to be fruitful (or not) for additional mutational efforts. For example, N457C fell outside the fruitful region (southern quadrant, Figure 2b) and exhibited substantially reduced selectivity (23%, Figure 2c), leading to its exclusion from combinatorial predictions.

### 2.4 Prediction Accuracy and Generalizability of PIFS‐PLM

A lack of data quality and quantity for specific conditions is often a bottleneck for enzyme sequence design in metabolic engineering [28], a situation that has encouraged the use of few‐shot learning methods [25, 46]. Although PLMs like ESM2 have shown impressive versatility in capturing transferable patterns across diverse tasks [24], fine‐tuning PLMs on small datasets can easily lead to overfitting [25]. Two common approaches for addressing overfitting include incorporating knowledge about enzyme function [47] and reducing variable complexity [48]. Our PIFS‐PLM uses both approaches, focusing on functionally relevant features and reducing variable dimensionality.

To benchmark PIFS‐PLM prediction accuracy, we first compared it against XGBoost‐PLM, a gradient boosting classifier [49] previously used to achieve top performance in the enzyme activity prediction task at the AI4Science Cup 2024. For LdTPS5 yield prediction, the success rate for exceeding the high‐yield threshold (>24 mg/L) was 0.5 (15/30) for XGBoost‐PLM versus 1.0 (10/10) for PIFS‐PLM (Table S5). PIFS‐PLM typically reaches an accuracy range of 0.7–0.8 on the training data, and maintains accuracy on the validation and test sets (Figure S1c). To evaluate the generalizability of PIFS‐PLM against XGBoost‐PLM, we compared their AUROC values using different data fractions (on MutDataset152, details see methods). XGBoost‐PLM models trained with 30%–100% of the data achieved AUROC values between 0.63 and 0.73, while PIFS‐PLM model attained AUROC values of 0.70–0.78 for the same data range (Figure 2d). Notably, using approximately 40% of the data (*N* = 60) obtained models with relatively good predictive accuracy (AUROC = 0.77). Therefore, the accuracy of PIFS‐PLM is apparently less sensitive to changes in data volume than XGBoost‐PLM, suggesting that. PIFS‐PLM has stronger generalizability.

Comparison with ProteinMPNN [13, 18] showed that PIFS‐PLM achieved 29.70‐fold and 1.64‐fold improvements in variant optimization for LdTPS5 and BdTPS11, respectively (Table S6). Notably, single mutants of LdTPS5 and BdTPS11 predicted with the MM/GBSA‐based approach respectively achieved 2.88‐fold and 6.52‐fold improvements over the best mutation predictions generated by ProteinMPNN.

To interrogate the basis of PIFS‐PLM predictions, we examined mutation sites across TP, TN, FP, and FN combinations. Minimum distances from each mutation site to substrate FPP showed no significant differences among the four categories (Figure S5a), indicating that substrate proximity does not determine prediction accuracy. Conservation analysis via MSA, however, revealed that V434C frequency differed markedly between TP and FP, and between TN and FN (Figure S5b), identifying V434C as a critical determinant for distinguishing high‐ from low‐yield mutants.

In the few‐shot learning scenario, obtaining generalizable models presents significant challenges, necessitating the incorporation of prior knowledge [50]. Our visualizations using local landscape maps illustrate the effectiveness of PIFS‐PLM in computational‐experimental iterations, allowing for the identification of fruitful regions during the optimization process. The method can improve enzyme activity with fewer than 200 experimental mutation combinations for training, which is a scale that is tenable and should provide value for both academic research and industry. Notably, the PIFS‐PLM framework requires only three essential inputs: A MSA, an active‐site definition, and minimal mutant combination data. As feature selection for this model relies on general evolutionary and physical principles rather than enzyme‐specific patterns, the PIFS‐PLM model is consequently theoretically applicable to any enzyme system and can be efficiently trained within just a few CPU hours.

In practical applications of PIFS‐PLM, it is not strictly necessary to limit the analysis to MSA or MM/GBSA for predicting activity‐enhancing single mutants. Experimentally validated activity‐enhancing single mutants can be used for mutation combination design, regardless of whether they are derived from random mutagenesis, rational design, or machine learning models. However, users need to employ random combinations or Bayesian optimization to generate the initial training dataset for PIFS‐PLM, and must also define the active site and conserved residues specific to the target enzyme (for example using the 3D structure and MSA) prior to the PLM feature selection step.

The general applicability of our workflow was further assessed with the divergent terpene synthase BdTPS11 (35.16% identity to LdTPS5). This enzyme catalyzes the formation of β‐macrocarpene from FPP, which is the precursor to the non‐volatile sesquiterpene phytoalexin zealexin A1, a compound with extensive antifungal and insecticidal activities [51]. We found that variants designed with PIFS‐PLM displayed > 10‐fold increases in β‐macrocarpene yields relative to BdTPS11^WT^ (Figure S6 and Table S7). In addition to optimizing enzyme activity, we explored whether the PIFS‐PLM framework could be extended to predict and improve enzyme selectivity. To this end, we applied the same modeling pipeline to a re‐labeled LdTPS5 dataset incorporating changes in product selectivity relative to the LdTPS5^WT^. Encouragingly, the model could identify promising candidates, and experimental validation showed that the top two predicted combinatorial mutants, respectively, displayed 11% and 15% higher selectivity than the LdTPS5^WT^ (Table S8). These results demonstrated that PIFS‐PLM has the potential to guide multi‐objective enzyme engineering beyond activity, including tuning selectivity.

### 2.5 PIFS‐PLM Improves Enzyme Activity

**FIGURE 3:**
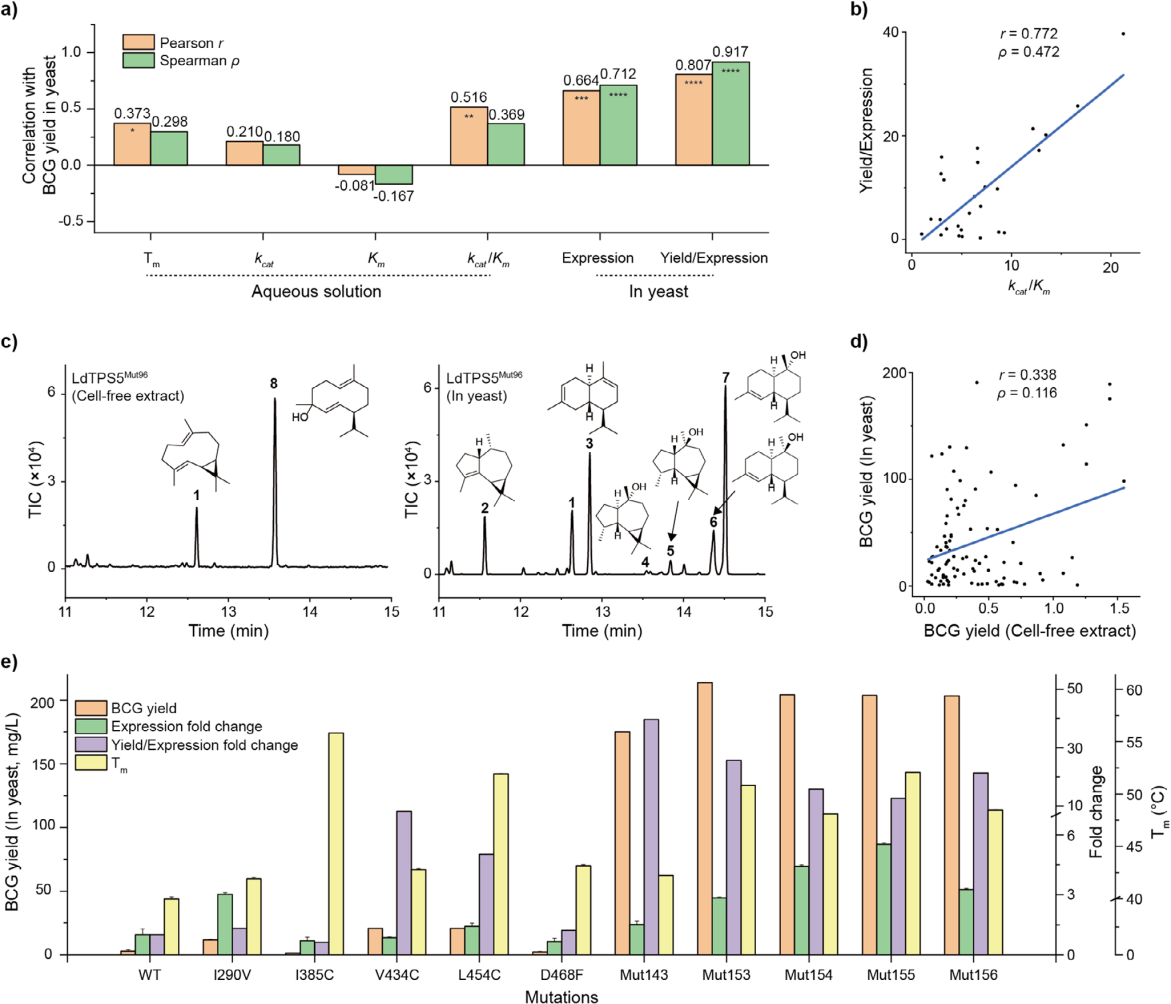
Optimizing for expression and unit yield is highly efficient as an alternative to optimizing for thermostability. (a) Pearson and Spearman correlation coefficients examining BCG yield with T_m_, *k_cat_*, *K_m_*, *k_cat_*/*K_m_*, expression (assessed using a fusion reporter), and unit yield (*i.e*., yield/expression). Statistical analysis was performed using unpaired two‐tailed Student's *t*‐test. *p* value: *p < 0.05, **p < 0.01, ***p < 0.001, ****p < 0.0001; no asterisks, not significant. (b) A strong positive correlation was detected between *k_cat_*/*K_m_* and unit yield. Pearson's *r* and Spearman's *ρ* are shown. (c) Certain mutants (e.g., LdTPS5^Mut96^) show dramatically different product profiles in yeast and cell‐free extract, which produced BCG and germacrene D‐4‐ol as major products in cell‐free extracts, but produced these two as minor products in yeast. (d) The Pearson's *r* and Spearman's *ρ* between in yeast and cell‐free extract BCG yield are weak. (e) BCG yield, expression, yield/expression, and thermostability for indicated mutants. Error bar represents the standard deviation of the mean.

To understand how mutation combinations enhance BCG yield, we examined whether the observed increases could be attributed to protein thermostability, *k_cat_*/*K_m_*, and/or expression levels (Figure 3a). We encountered challenges in analyzing the purified LdTPS5 mutants, as protein inclusion bodies invariably formed. Consequently, we employed three datasets: Purified enzymes, in yeast data, and cell‐free extracts (Tables S4, S5, and S9). We observed a strong correlation between *k_cat_*/*K_m_* from purified enzymes and unit yield in yeast, with Pearson and Spearman coefficients of 0.772 (*p* < 0.0001) and 0.472 (*p* = 0.013), respectively, implying that unit yield may serve as a reliable estimate of in vivo enzyme activity (Figure 3b).

To assess whether BCG yield and product profiles were consistent across experimental systems, BCG production was measured for each mutant in both cell‐free extracts and yeast (Table S9). Interestingly, the product profiles of some mutants in yeast were very different from those of the same mutants in cell‐free extracts. For example, LdTPS5^Mut96^ produced BCG and germacrene D‐4‐ol as major products in cell‐free extracts, but produced these two as minor products in yeast (Figure 3c). Furthermore, we observed a weak correlation between these two different experimental conditions, with Pearson and Spearman coefficients of 0.338 and 0.116, respectively (Figure 3d). We observed a moderate correlation between the aqueous *k_cat_*/*K_m_* and the BCG yield in yeast, with Pearson and Spearman coefficients of only 0.516 and 0.369, respectively (Figure S7). These results indicate that the product profile difference between in vitro and in vivo experiments must be taken into account for activity optimization and, moreover, suggest that using in vivo unit yield is likely the more promising approach.

We then sought to investigate how changes in T_m_ and expression levels affect BCG yield in yeast (Figures 3e and S7). Notably, all single mutants exhibited increased T_m_, indicating improved thermostability compared to the LdTPS5^WT^. The correlation between T_m_ and BCG yield is moderate, with Pearson and Spearman coefficients of 0.373 and 0.298, respectively. Moreover, we also found that increased LdTPS5 expression levels were strongly correlated with increased BCG yield in yeast, with Pearson and Spearman coefficients of 0.664 and 0.712, respectively (Figure 3a). In many cases, optimizing expression levels is sufficient; the focus in metabolic engineering often lies on refining the expression system rather than the enzyme sequences themselves. By initially selecting activity‐enhancing single mutants, the mutation combinations predicted by PIFS‐PLM can improve both enzyme activity (unit yield) and expression.

**FIGURE 4:**
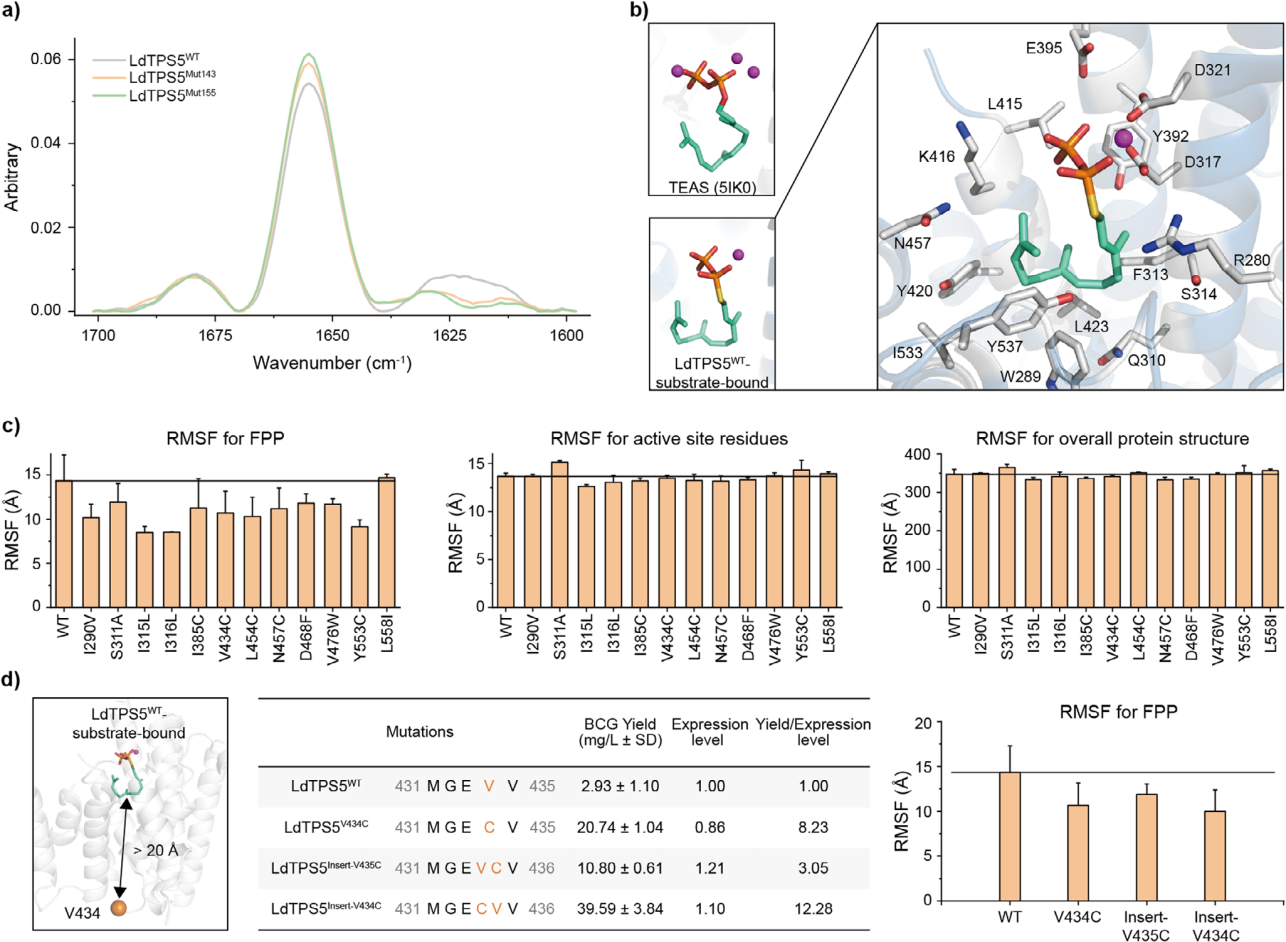
Structural basis of activity‐enhancing mutations. (a) MMS spectral differences between LdTPS5^WT^‐FsPP and mutant–FsPP complexes. (b) Crystal structure of LdTPS5^WT^ bound to the substrate analog FsPP at 2.8 Å resolution. (c) RMSF of FPP (modeled from the crystallographic FsPP), active site residues, and the overall protein for LdTPS5^WT^and mutants, calculated over the final 10 ns of 300 ns MD simulations. (d) Experimental results (left) and MD data (right) for LdTPS5^V434^ sequence variants. Error bars represent the standard deviation of the mean.

Given the understanding that mutations often affect enzyme activity by altering the protein structure [52], we examined whether activity‐enhancing mutations affect protein structure, initially by using microfluidic modulation spectroscopy (MMS) to probe for structural change events in the mutant proteins. We observed differences in the MMS spectra between the LdTPS5^WT^ and three mutants (Figures 4a and S8). We then attempted to determine the structures of the LdTPS5^WT^ and mutants. We successfully obtained substrate‐free and substrate‐bound crystal structures of LdTPS5^WT^ (Figure 4b and Table S10), as well as a substrate‐free structure of LdTPS5^Mut143^ (I290V/I385C/V434C/L454C/V476W/L558I), which had a 21‐fold enhancement in aqueous *k_cat_*/*K_m_* (Table S4). Of note, despite extensive efforts, we were unable to obtain the substrate‐bound crystal structures for LdTPS5^Mut143^ or for other high‐activity mutants.

The overall LdTPS5^WT^‐substrate‐bound structure is similar to a homology model that we initially generated using 5‐epi‐aristolochene synthase from tobacco as the template (PDB: 5IK0, RMSD = 1.5 Å), supporting that our starting homology model used for MM/GBSA calculations is reasonable. Our size exclusion chromatography data for purified LdTPS5^WT^ and for various mutants indicated that protein predominantly exists as a monomer in solution (Figure S9), yet all of the crystal structures were dimers. These differences may arise from the fact that the conformations observed in the crystal structure do not correspond to the stable conformations in solution [53]. Notably, despite high similarity between the monomer crystal structures of LdTPS5^WT^ and LdTPS5^Mut143^ (Cα RMSD = 0.34 Å; Figure S10a), we observed localized conformational differences in certain active site residues. For instance, residue T417 exhibits an RMSD value of 1.02 Å. Previous studies of two (E)‐β‐farnesene synthases have identified this site as associated with *k_cat_*, where mutations resulted in 4.0‐ and 6.7‐fold decreases in *k_cat_* [54]. In this study, LdTPS5^Mut143^ exhibits a 6.23‐fold increase in *k_cat_* and a 3.35‐fold decrease in *K_m_* compared to LdTPS5^WT^. This finding suggests that combining specific mutations can lead to synergy between the catalytic (*k_cat_*) and substrate binding (*K_m_*) steps.

To explore how the relevant mutations affect LdTPS5, we carried out extensive MD simulations of LdTPS5^WT^ and all 12 single mutants using their predicted monomer structures (Figures 4c and S11–S15). Conventional approaches for enhancing catalytic efficiency typically involve two principal mechanisms: Either restricting the conformational freedom of the substrate, FPP, to promote its shift toward a reactive, high‐energy conformation, or lowering the transition state energy to reduce the activation barrier for the catalytic step. These simulations predicted that root‐mean‐square fluctuation (RMSF) of the substrate FPP is reduced by an average of 24.4% in the single mutants that displayed enhanced enzymatic activity, suggesting increased stability in the substrate binding conformation (Figure S11a). Notably, no change was predicted for the overall RMSF of LdTPS5 versus any of single mutant monomers we examined (Figure S12), implying that these mutations facilitate stabilization of the substrate in an apparently reaction‐favorable conformation.

Despite the potential for an entropic cost, wherein reduced flexibility enhances substrate binding (reflected in a lower *K_m_*), but compromises *k_cat_*, our observations of improved overall *k_cat_*/*K_m_* ratio in experimental data for the 12 single mutants demonstrate a net positive effect on catalytic efficiency (Table S4). Moreover, our data indicate that these 12 single mutants had heterogeneous effects on enzyme kinetics, with seven mutations (*e.g*., D468F and I290V) increasing *k_cat_* while concurrently decreasing *K_m_*, four mutations selectively optimizing *k_cat_* (e.g., L454C and V476W), and one enhancing only *K_m_* (L558I). Overall, these coordinated changes in *k_cat_* and *K_m_* resulted in synergistic improvements in catalytic efficiency (*k_cat_*/*K_m_*) of these mutants. This stabilization idea is supported by our aforementioned experimental results showing the increased activity (*k_cat_*/*K_m_*; Figure S3e). Additionally, an analysis of the RMSF of the active site residues did not reveal a consistent trend; that is, both increases and decreases in RMSF were observed among the mutants. This lack of clear pattern suggests that, the stabilization of the substrate conformation may be driven by long‐range interactions.

We then selected the mutation site furthest from the active site, V434C, and generated two more sequence variants with cysteine residues inserted before or after V434. Experimental validation showed that LdTPS5^Insert‐435C^ and LdTPS5^Insert‐434C^ exhibited 3.05‐ and 12.28‐fold higher unit yield compared to LdTPS5^WT^, respectively (Figure 4d). Protein Structure Network (PSN) analysis [55] revealed an enhanced long‐range communication pathway in V434C, absent in LdTPS5^WT^, connecting the mutation site to active site residues Q310 and W289 (Figure S16). Meanwhile, MD simulations suggested that these mutations contribute to stabilizing the substrate conformation. This stabilization likely lowers the activation energy required for substrate catalysis, thereby directly contributing to the improved yields/expression levels observed in the combinatorial mutants. Notably, the LdTPS5^Mut143^‐substrate‐free structure did not show disulfide bond formation at the mutation site, which argues against any contribution from such bond(s) in the observed enzyme activity increase (Figure S10b). Taken together, these findings suggest that, distal mutations affect local structures and propagate these changes to the active site, and these changes likely enhance activity by facilitating the binding of the small molecule substrate in a reaction‐favorable conformation compared to LdTPS5^WT^.

### 2.6 A Causal Inference Model to Clarify Relationships Among Enzyme Thermostability, Activity, and Expression

**FIGURE 5:**
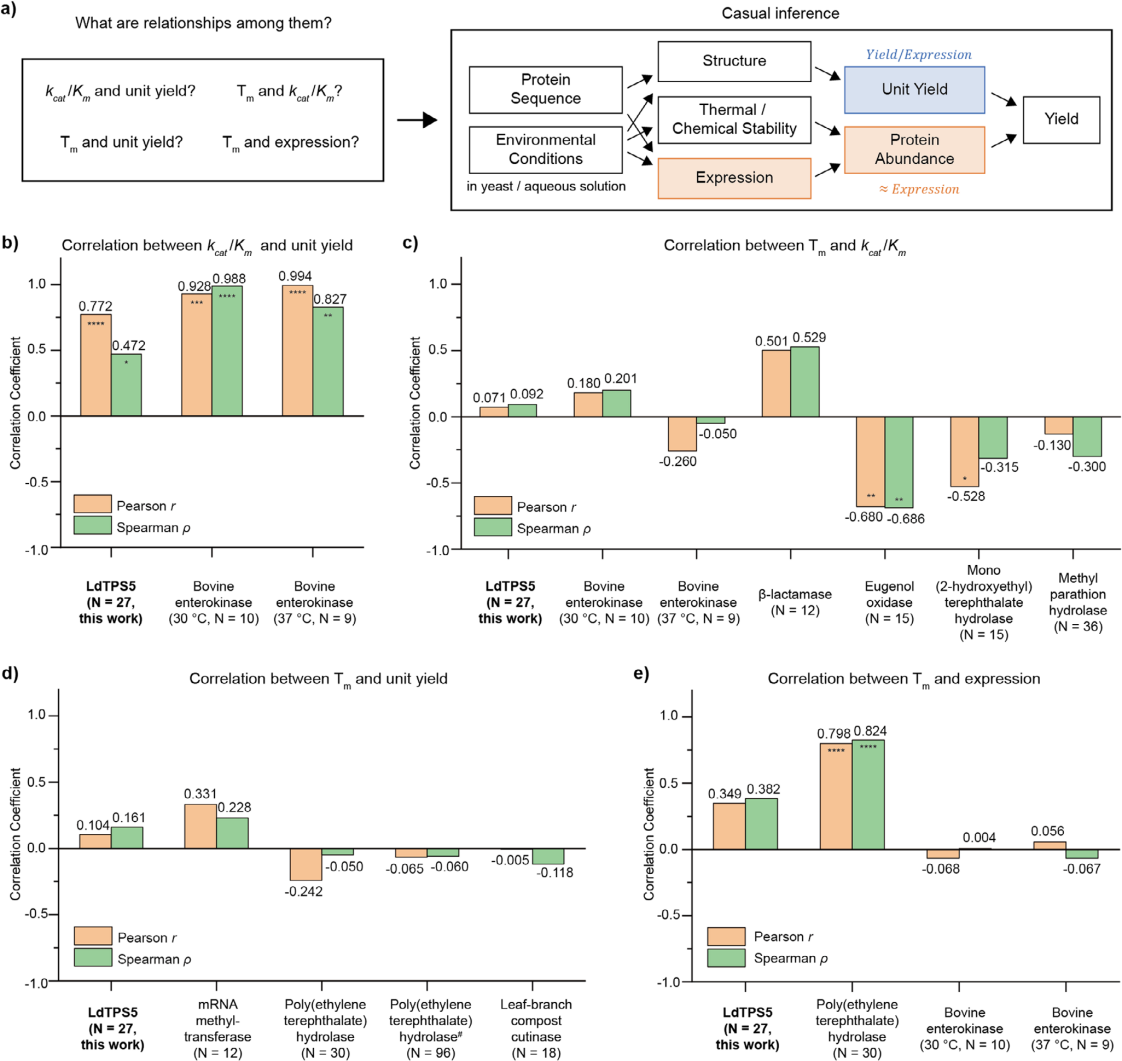
Causal inference model and correlation analyses of enzyme properties. (a) Diagram of variables affecting enzymatic yield. (b) Correlation between *k_cat_*/*K_m_* and unit yield for the indicated enzymes. (c) Correlation analysis between T_m_ and *k_cat_*/*K_m_* for the indicated enzymes. (d) Correlation analysis between T_m_ and unit yield for indicated enzymes. (e) Correlation analysis between T_m_ and expression level for the indicated enzymes. Statistical analysis was performed using unpaired two‐tailed Student's *t*‐test. *p* value: *p < 0.05, **p < 0.01, ***p < 0.001, ****p < 0.0001; no asterisks, not significant. *N* represents sample number; ^#^denotes different studies of the same enzyme. A total of 27 representative LdTPS5 mutants are shown; see Table S4 for detailed experimental data of each mutant.

To investigate the factors contributing to product yield enhancement at both the cellular and culture levels, we propose a theoretical model represented by a causal inference diagram (Figure 5). In this model, enzyme sequences and environmental conditions serve as causes, yield serves as the effect, and intermediate variables include activity, thermostability, and expression levels. Environmental conditions refer to different expression/reaction systems. Thermostability is defined as the melting temperature (T_m_) of the enzyme in vitro, and expression levels are quantified using the P2A‐mediated EGFP co‐expression and fluorescence assay (Methods), in yeast or in other living systems.

Since sequence editing can concurrently affect enzymatic activity, stability, and expression, separating these factors can in theory better guide enzyme optimization. We can assume two variables: Unit yield and protein abundance (Figure 5a). While measuring aqueous *k_cat_*/*K_m_*, the gold standard for enzyme activity, is time‐consuming, we posit that “unit yield”, specifically yield divided by expression level, can serve as a surrogate for aqueous *k_cat_*/*K_m_*. We found a strong correlation between unit yield (in vivo) and aqueous *k_cat_*/*K_m_*, through analyzing literature data and our own experiments (Figure 5b). The relationship between thermostability and enzyme activity is still controversial: Enzyme activity is not highly correlated with stability [56]. We reviewed various types of enzymatic data from the literature, including in vitro experiments and metabolic engineering efforts in *Escherichia coli*, *Saccharomyces cerevisiae*, and *Pseudomonas putida*. Our analysis revealed no significant correlation between in vitro activity (*k_cat_*/*K_m_*) and in vitro thermostability (T_m_), with 6 out of 7 cases showing Pearson and Spearman coefficients below 0.2 (Figure 5c,d). Therefore, optimizing for thermostability can help preclude structurally unstable proteins, but seems unlikely to yield large gains for enzyme activity enhancement.

In one out of the four cases for which expression data was available, we detected a strong correlation (Pearson and Spearman coefficients > 0.7) between the thermostability of mutant variants and protein expression levels (Figure 5e). This suggests that improved thermostability can increase the quantity of effective enzyme in cases where the WT enzyme is thermally unstable, thereby enhancing overall product yield; this scenario helps explain how stability optimization can sometimes (but not always) lead to the identification of high‐yield mutants. In our causal inference model, T_m_ acts as an indirect factor; once protein abundance is measured and unit yield calculated, T_m_ requires no further consideration. While optimizing stability is a viable approach, optimizing expression levels is likely to be more efficient, as thermostable sequences with low expression still result in low product yield. Therefore, in this study, we prioritized optimizing for enzyme activity and expression over thermostability. Further, for substrates that are difficult to synthesize, in vitro experiments can be costly. Measuring product yield and expression levels provides a direct means to calculate unit yield, which we found to be highly correlated with enzyme activity (Figure 5a,b). To obtain meaningful experimental activity data, iterations should be conducted under consistent experimental conditions [57]. However, given a dramatic difference between product profiles/yield in vitro and in vivo (Figure 3c,d and Table S9), it remains unclear which should be prioritized for training a model. Based on unit yield defined in our model, in vivo activity data can be used directly without considering in vitro *k_cat_*/*K_m_*.

## 3 Conclusion

Biotechnology relies on engineered enzymes to produce valuable chemicals, but improving their catalytic performance typically requires extensive experimentation. Conventional screening methods mostly identify single mutations, and systematically exploring the vast combinatorial space to discover top‐performing variants remains prohibitively expensive. In this study, through causal inference analysis, we examined the relationships among variables influencing enzyme catalysis in the context of metabolic engineering. We propose that unit yield, defined as yield divided by expression level, can be understood as a viable substitute for aqueous *k_cat_*/*K_m_* in assessing in vivo enzyme activity, offering a cost‐effective and time‐efficient alternative. We developed an integrated workflow that combines a physics‐inspired step for single mutation prediction with a protein‐language‐model‐based few‐shot learning step to select suitable mutation combinations. This strategy achieved a 73‐fold increase in product yield (or a 15% gain in selectivity) from sparse experimental data. PIFS‐PLM demonstrated superior accuracy and generalizability compared to Bayesian optimization, XGBoost‐PLM, and ProteinMPNN. A further advantage of this approach is its generation of local landscape maps that can be used to identify enzyme regions that are likely to be fruitful (or not) for additional mutational and engineering efforts. This workflow, which requires minimal experimental data and computational resources, enables efficient enzyme engineering and has significant potential for both academic and industrial applications, providing a scalable method for optimizing enzyme activity.

## Author Contributions

**Lin Guo**: investigation, writing – original draft, methodology, visualization, formal analysis. **Yukun Li**: investigation. **Xiaoguang Yan**: investigation, funding acquisition, writing – original draft. **Hongxiao Tan**: investigation. **Xiling Chen**: investigation. **Mingyue Ge**: investigation. **Yali Lu**: investigation. **Shilong Fan**: visualization. **Dongmei Liang**: investigation. **Weiguo Li**: investigation. **Shengxin Nie**: investigation. **Yefeng Tang**: writing – review editing. **Boxue Tian**: conceptualization, funding acquisition, writing – original draft, writing – review editing, methodology, formal analysis, project administration, supervision, resources. **Yihan Zhao**: investigation. **Jianjun Qiao**: conceptualization, funding acquisition, writing – review editing, project administration, resources, supervision. **Xiaochun Zhang**: investigation visualization.

## Conflicts of Interest

The authors declare no conflicts of interest.

## Supporting information




**Supporting File**: anie73297‐sup‐0001‐SuppMat.pdf.

## Data Availability

The data that supports the findings of this study are available in the supplementary material of this article.
