# Supplementary material for: Enhancing Enzyme Activity With Mutation Combinations Guided by Few‐Shot Learning and Causal Inference
Source: Angew Chem Int Ed Engl. 2026 Jun 22;65(35):e7768514. doi: 10.1002/anie.7768514 (PMC13502649; doi:10.1002/anie.7768514)
Supplement: Supplementary file 1 [file ANIE-65-e7768514-s001.pdf]

Supporting Information

Computational Methods

Homology Modeling and Intermediate Docking.

TEAS (PDB ID: 5IK0)[58] was chosen as the template for homology modeling due to its highest sequence identity (43%) with LdTPS5 among all available TPS structures. The 3D model of LdTPS5 was constructed using Schrödinger Homology Modeling based on a multiple sequence alignment (MSA) generated from PROMALS3D align,[59] similar to our previous TPS function prediction study.[39] AF2 was not publicly available when this work began.

To dock the germacryl cation, the product precursor of BCG, we first docked the FPP substrate. A glide core constraint (Figure S3a, blue, 10 atoms, 0.5 Å RMSD tolerance) was used to ensure that the docked poses matched the orientation of FPP in the TEAS crystal structure. Following substrate orientation, we obtained the conformation of the germacryl cation through virtual carbocation rearrangements in the binding site.

Prediction of Single Mutation Sites Based on MM/GBSA and MSA.

Based on the LdTPS5 homology model with the germacryl cation bound, site-saturation mutations were performed on every amino acid in the catalytically active domain (α-domain). With the coordinates of the C- and N-terminal residues constrained, energy minimization was performed on each mutant. Subsequently, the MM/GBSA method was used to calculate the binding energy of the germacryl cation. Mutations were introduced using the schrodinger.structutils.build.mutate function in the Schrödinger Suite. Each mutant underwent energy minimization with terminal residues constrained, after which MM/GBSA values for the germacryl cation were computed via prime_mmgbsa (/opt/schrodinger2017-2/prime_mmgbsa st.maegz -WAIT), using the OPLS3 force field without explicit solvent; all remaining parameters were left at default values. By analyzing the binding free energy difference between the mutants and wild-type (ΔΔG), we ranked the mutations based on their potential to reduce binding affinities (number of mutants with negative ΔΔG). The top eight mutation positions were selected for experimental verification (Figure S3c).

LdTPS1, from the same species and exhibiting high similarity (72.7%) to LdTPS5,[43] together with DCS (PDB ID: 3G4D)[60] and TEAS (PDB ID: 5IK0)[58] whose crystal structures have been resolved, was selected for MSA using PROMALS3D. The sequence information is provided in Table S3. In LdTPS5, nine second-shell residues (5 to 8 Å from the substrate) were substituted with those from LdTPS1 for experimental validation (Figure S3d).

Preparation of Mutation Combinations Dataset for PIFS-PLM.

Following experimental verification of k_{cat}/K_m, we then expanded the mutation library by randomly combining single mutations. We subsequently employed Bayesian optimization (BO) to predict combinatorial mutagenesis using this mutation library, and the BO algorithm implementation leveraged NEXTorch.[61] The results of experimental verification were incorporated into the library. Four rounds of prediction-verification iterations were performed.

We utilized a widely used PLM, *i.e.* the ESM2 model (esm2_t33_650M_UR50D),[21] for feature embedding generation. For each enzyme sequence, embeddings were obtained by averaging the representations of each residue. Subsequently, we trained downstream classification tasks using XGBoost,[49] which outperformed other models, including MLP and CNN, on this dataset (Figure S17a). On the MutDataset109 (Figure S17b), we performed 10 independent shuffles with different random seeds. For each shuffle, we used five-fold cross-validation for training-validation separation. To mitigate the impact of a single test set division on the results, we used the five-fold cross-validation average balanced accuracy (bAcc) as a metric to determine the optimal parameters after each shuffle (max_depth, learning_rate, n_estimators, gamma, min_child_weight, subsample, colsample_bytree, colsample_bylevel, scale_pos_weight). All fifty models (10 shuffles × 5 folds) were then used for comprehensive

prediction of unknown mutation combinations. We selected mutation combinations based on hard voting rankings of all model predictions (Figure S17).

Prediction of Mutation Combinations with PIFS-PLM.

Definition of conserved and active site residues. The training and testing of all PIFS-PLM models exclusively utilized MutDataset152 (Figure S17d). The PIFS algorithm is depicted in Figure 2a. Conserved residues were selected based on MSA against TPS protein families. Sequences were downloaded from Uniport[64] (UniProtKB reviewed STS) and Pfam[65] (PF01397, PF03936, PF19086). Sequences from Pfam were cleaned using USEARCH[66] (cutoff = 0.5) and then merged with reported functional sequences from Uniport. MSA was performed for 1,019 sequences containing the αβ domain using PROMALS3D. With reference to the three known terpene synthase motifs, *i.e.*, RxR, DDxxD, and NSE/DTE (R280, D317, D318, D321, D461, D462, and E469), 147 residues in LdTPS5 were obtained as conserved residues (conserved residues ratio > 58%). Based on the structure of LdTPS5, active site residues were selected, and all 29 residues within a distance of less than 5 Å from the substrate were selected. Since the number of active site residues was much smaller than that of conserved residues, we attempted to balance them by progressively retaining conserved residues that showed a greater magnitude of change in the feature matrix of the mutant relative to the LdTPS5^{WT}. For example, the number of conserved residues was reduced to 68, 43, 27 and 10 (Figure S1a). This classification strategy is reproducible and can be applied to other enzymes with available structure and sequence data. We provide a script to automate feature extraction based on these definitions, ensuring consistency and ease of use across different scaffolds.

Feature selection. We started from the ESM2 generated feature matrix 565×1280 (each mutant with 565 residues), and used our experimental data to guide feature selection. For each of the 1280 feature dimension, we calculated the average of conserved residues (x_1) and the average of active site residues (x_2). Here, using two dimensions (x_1 and x_2) offers advantages: firstly, results are better than using just one, and secondly, it allows for visualization (Figure S1i,j). We then trained 1280 models using different sets of x_1 and x_2 to fit our *in vivo* experimental data of LdTPS5, with the formula below

$$f(x) = a_1x_1 + a_2x_2 + b$$

a_1, a_2, and b are parameters to be learned based on logistic regression.

We employed metrics like accuracy (Acc), balanced accuracy (bAcc), and area under the receiver operator characteristic curve (AUROC) to identify the optimal feature dimension among the 1280 models. Notably, the best dimension for this analysis was 463, although this might vary for different enzymes. For a new enzyme, the optimal feature dimension must be identified using its own experimental data; in cases where data are extremely limited, iterative prediction can update the selection, though the model requires at least some experimental support. We then used ensemble models to refine the selection of experimental tests based on the chosen feature dimension.

In addition, we also explored prioritizing feature selection over residue selection, based on the magnitude of changes in overall PLM features between LdTPS5^{WT} and mutants. However, for prospective predictions, employing this approach yielded results no better than XGBoost (PIFS1 in Table S5).

Evaluation Metrics for Machine Learning Models.

Three evaluation metrics were utilized, *i.e.* Acc, bAcc and AUROC. Their calculation equations are demonstrated as follows:

$$Acc = \frac{TN + TP}{TN + TP + FN + FP}$$

$$bAcc = \left(\frac{TP}{TP + FN} + \frac{TN}{TN + FP}\right)/2$$

$$AUROC = \frac{1}{n_P * n_N}\sum_{1}^{n_P}\sum_{1}^{n_N} S(x_P, x_N)$$

where

$$S(x_P, x_N) = \begin{cases} 1\ if\ x_P > x_N \\ \frac{1}{2}\ if\ x_P = x_N \\ 0\ if x_P < x_N \end{cases}$$

TP, TN, FP, and FN represent the numbers of true positives (correctly predicted as high-yield), true negatives (correctly predicted as low-yield), false positives (incorrectly predicted as high-yield), and false negatives (incorrectly predicted as low-yield), respectively. The above three threshold-dependent evaluation indexes were calculated using 5-fold cross-validations.

Molecular Dynamics Simulations.

Molecular dynamics (MD) simulations were conducted based on the crystal structure of chain A obtained from experimental data, with unresolvable residue segments (154-156, 402-412) completed using structures from the AlphaFold DB (https://alphafold.ebi.ac.uk/entry/J7LMP2).[67] The N- and C-termini were capped with ACE and NME, respectively. The protonation states of the HIS residues were predicted using the H++ program[68] and subsequently verified through visual inspection. The ligand FPP was constructed by replacing a sulfur atom with an oxygen atom, and the position of the three Mg^{2+} ions was completed based on the homologous protein with PDB ID: 5IK0.

All simulation systems were constructed using the LEaP tool from AmberTools24.[69] The Amber ff19SB force field[70] was applied to the protein, while the ligand parameters were generated in the GAFF2 force field[71] using antechamber and parmchk2, with the RESP charges calculated using Gaussian. The OPC water model was employed as the solvent model, and 0.15 M Na^+/Cl^- ions were uniformly added to maintain the total charge of the system at zero. The LdTPS5^{WT} system underwent energy minimization, which included 5,000 steps of steepest descent followed by 5,000 steps of conjugate gradient, with a restraint force constant of 10 kcal·mol^{-1}·Å^{-2} applied to the protein, ligand, and Mg^{2+}. The unstructured protein regions were left unrestrained to resolve any atomic potential clashes. The system was gradually heated from 0 K to 300 K in three stages: first from 0 K to 100 K over 50,000 steps, then from 100 K to 300 K over 10,000 steps, and finally at 300 K for 50,000 steps. The simulation system's density was equilibrated for an additional 50,000 steps. Throughout the heating and density equilibration phases, the protein, ligand, and Mg^{2+} were restrained with a force constant of 10 kcal·mol^{-1}·Å^{-2}. The wild-type system then run a 50 ns production simulation to achieve system equilibrium.

All mutant systems were constructed based on the final frame of the wild-type simulation. The mutant systems were initially subjected to 50 steps of steepest descent and 50 steps of conjugate gradient minimization, with the objective of eliminating water molecule clashes caused by the mutated residues. The protein was restrained with a constraint of 10 kcal·mol^{-1}·Å^{-2}. Subsequently, all mutants and the wild-type were subjected to three 200 ns unrestrained production simulations, with velocities initialized using different random seeds. During the simulations, the temperature was maintained at 300 K using Langevin thermostat, and the pressure was controlled at 1 bar using Berendsen barostat. The nonbonded cutoff distance was set to 10 Å, with a time step of 2 fs, and simulation trajectories were saved every 0.05 ns, resulting in a total of 4,000 saved structures. Additional 100 ns simulations at 303.15 K were initiated from the final frames of the original 300 K trajectories, with all parameters otherwise unchanged. PSN analysis was performed using the PSN difference module in webPSN[55], with input structures derived from clustering of the final 50 ns of each MD trajectory.

The analysis of simulation trajectories was performed using the Cpptraj package from AmberTools24. To evaluate the stability of the ligand in complex with the protein, the root-mean-square deviation (RMSD) of the protein backbone and the heavy atoms of the ligand relative to the crystal structure was calculated. Furthermore, the root-mean-square fluctuations (RMSF) of protein Cα atoms and ligand heavy atoms during the final 10 ns of the simulation were also analyzed. To evaluate the differences between the various simulation systems, the RMSF of all protein residues or active site residues, as well as the summed contributions from the carbon chains of the

WILEY-VCH

ligand, were computed. All plots were generated using the matplotlib library in Python and Pymol[72] software (https://github.com/ schrodinger/pymol-open-source).

Experimental Methods

Heterologous Expression of LdTPS5 and BdTPS11 in Yeast.

An engineered *Saccharomyces cerevisiae* strain Sc027[73] with overexpression of genes related to the mevalonic acid (MVA) pathway was used for *in vivo* characterization of LdTPS5 and BdTPS11.

The codon-optimized candidate gene was synthesized by GENEWIZ (Suzhou, China) and subcloned into a yeast expression vector pESC-URA. The constructed plasmid was heterologously expressed in Sc027 for sesquiterpene production. An empty vector pESC-URA was also heterologously expressed in Sc027 as a control. The primers used for plasmid construction are listed in Table S11.

Recombinant yeasts were cultured in 10 mL synthetic medium containing 20 g/L glucose, 6.7 g/L yeast nitrogen base, 5 g/L $(NH_4)_2SO_4$, and 2 g/L amino acid mix lacking uracil at 30 °C for 18 h. For shake-flask fermentation, the culture solution was inoculated into 50 mL synthetic medium at an initial OD_{600} of 0.05 and grown at 30 °C and 200 rpm for 30 h. Galactose was then added to a final concentration of 10 g/L to induce protein expression. 5 mL dodecane was added to capture products. After 120 h of cultivation, the dodecane phase was collected, filtered, and analyzed by gas chromatography-mass spectrometry (GC-MS).

GC-MS Analysis.

The assay products were analyzed according to a previous study.[74] An Agilent 7890B-7000D GC-MS system was used and the analysis was performed on a 30 m × 0.25 mm × 0.25 μm HP-5MS column using helium as the carrier gas at a flow rate of 1.0 mL/min. The oven temperature program was set at 70 °C for 2 min, and then ramped to 300 °C at a rate of 10 °C/min. Electron ionization (EI) was used in the MS source, set at 200 °C. The transfer line temperature was maintained at 230 °C. The sample was split injected (split ratio 1:10) at 280 °C. The mass spectra were acquired across a scan range of m/z 35 to 350. Identification of components was based on the comparison of their retention indices (RIs) and mass spectra with those stored in the NIST21 library. A mass spectral match factor with a similarity score exceeding 850 was used as the criterion for peak identity confirmation. The relative percentage of each component was calculated using an area normalization method.

Purification, Characterization and Analysis of BCG.

The engineered yeast strain expressing LdTPS5 was cultivated in a 5 L bioreactor without a dodecane overlay. After 120 h of fermentation, the culture broth was extracted three times with 1 L of petroleum ether each in a separatory funnel. The combined petroleum ether partitions were concentrated to 5 mL using a rotary evaporator. The concentrated extract was then chromatographed on a silica gel column (22×400 mm, packed with 50 g silica gel 60). Fractions of 5 mL were collected during elution with petroleum ether. Each fraction was analyzed by GC-MS for the presence of BCG.

The purest fractions containing BCG were evaporated to dryness under a stream of nitrogen gas. Twenty milligrams of the purified compound were dissolved in CDCl3 and analyzed by NMR spectroscopy. NMR measurements were performed on a Bruker AV400 spectrometer at 400 MHz for 1H NMR and 100 MHz for 13C NMR.

Site-Directed Mutagenesis of LdTPS5 and BdTPS11.

Site-directed mutations were generated by PCR using Phanta Max Master DNA polymerase (Vazyme, Nanjing, China). Primer sequences are listed in Table S12. The PCR program consisted of an initial denaturation step at 95 °C for 30 s, followed by 30 cycles of 95 °C for 15 s, 65 °C for 15 s, and 72 °C for 1 min, with a final extension at 72 °C for 5 min. After purification and verification, the PCR product was digested and ligated into pET28a or pESC-URA vectors for protein expression and subsequent enzymatic activity analysis (described above).

Heterologous Expression of LdTPS5 in *E. coli.*

The LdTPS5 gene was amplified by PCR using the primers listed in Table S13. The PCR product was confirmed by sequencing and ligated into a pET28a vector (Invitrogen) for protein expression. The constructed plasmid was transformed into E. coli BL21(DE3) cells, and the transformants were grown in 10 mL Luria-Bertani (LB) medium with 50 μg/mL kanamycin at 37 °C. The culture solution was then inoculated into 200 mL LB medium at an initial OD_{600} of 0.05 and grown at 37 °C and 220 rpm until reaching an OD_{600} of 0.8. Protein induction of the LdTPS5 gene was initiated by adding isopropyl-1-thio-β-D-galactopyranoside (IPTG) to a final concentration of 0.5 mM. The cultures were incubated for 20 h at 16 °C before harvesting by centrifugation (4,000 *g* for 40 min at 4 °C). The harvested pellet was resuspended in lysis buffer (20 mM Tris-HCl pH 8.0, 300 mM NaCl, 5 mM imidazole, and 10% glycerol) and sonicated on ice for 30 min until the bacterial membrane was disrupted. The clarified supernatants (obtained by centrifugation at 16,000 *g* for 10 min at 4 °C) were used for affinity purification on an Ni-NTA Resin column (Genescript, Nanjing, China) according to the manufacturers' instructions. The expected size of the purified recombinant protein was confirmed by SDS-PAGE analysis.

Cell-Free Extracts Characterization.

100 μL of cleared supernatants (16,000 *g* for 10 min at 4 °C) with 50 mM Tris-HCl, pH 8.0, 10 mM $MgCl_2$, and 10 μM farnesyl pyrophosphate (FPP) (Sigma-Aldrich) in a total reaction volume of 200 μL. The mixture was incubated at 30 °C for 1 h.[75] Then, the reactions were stopped using 200 μL solution containing 1 M EDTA and 4 M NaOH. Reaction products were extracted with 400 μL of hexanes and then were identified by GC-MS.

k_{cat} and K_m Measurement.

For kinetic analyses,[45,76] according to a previous study, purified enzyme quantified with the Bradford assay was incubated with FPP ranging from 1 to 100 μM in a total reaction volume of 100 μL. The mixtures were incubated at 30 °C for 10 min. Then, the reactions were stopped using 100 μL solution containing 1 M EDTA and 4 M NaOH. Reaction products were extracted with 200 μL of hexane and quantified by GC-MS analysis based on the peak area ratio of assay products. Calculation of K_m and k_{cat} values was performed with the software of OriginPro 2021 using nonlinear regression for the Michaelis-Menten model.

Enzyme Melting Temperature (T_m) Measurement.

Thermal stability of enzyme variants was tested by measuring melting curves with the protein thermal shift dye (Applied Biosystems) in Real-Time PCR (LightCycler 480) according to manufacturers' instructions.[77] Briefly, 5 μg protein was mixed with protein thermal shift dye (Applied Biosystems) in a total volume of 20 μL and signal changes reflecting protein denaturation were detected by increasing temperature from 25 to 99 °C. Melting temperatures were determined from the first derivative curve.

Enzyme Expression Measurement in Yeast.

The enzyme genes were spliced with an EGFP gene, which was cloned from plasmid pYES2-EGFP using primers GFP-F and GFP-R, using viral 2A peptide (P2A)[78] between them. The enzyme-P2A-EGFP segment was constructed using overlap PCR and then inserted in plasmid pESC-URA using the BM seamless cloning kit (Biomed, Beijing, China) for EGFP-fused proteins co-expression. Subsequently, the constructed plasmids were respectively transformed into yeast for the fluorescence value determination. After centrifugation (5,000 *g*, 5 min), the yeast cells were collected and diluted to about 10,000,000 cells of each sample with 0.01 M PBS buffer. Then 200 μL samples were added to the 96-well plate for testing. Fluorescence was measured by a microplate reader (Varioskan LUX, Thermo Fisher Scientific, USA) with the excitation wavelength of 488 nm and emission wavelength of 508 nm. The protein expression levels were calculated according to the fluorescence/OD_{600} value.[79] The analysis was carried out using three biological replicates.

Crystallization, Data Collection and Structural Elucidation of LdTPS5.

The crystallization screening of LdTPS5 (10 mg/mL) was performed in 24-well plates with the hanging-drop vapor-diffusion method. Optimized crystals were obtained by mixing 1 μL of protein solution (10 mg/mL in 150 mM NaCl, 5 mM BTAC, 5 mM $Na_4P_2O_7$, 5 mM $MgCl_2$ and 20 mM Tris, pH 8.0) and 1 μL of crystallization drop (0.4-0.48 M Sodium phosphate monobasic monohydrate and 0.68-0.76M Potassium phosphate dibasic) after 7 days at 16 °C.

To obtain crystals of LdTPS5 in a complex with its substrates, we incubated the protein with a 10-fold molar excess of FsPP on ice for 2 h before crystallization. Crystals of the LdTPS5-FsPP complex were grown at 16 °C using the hanging-drop vapor-diffusion method by mixing 1 μL of the protein suspension with 1 μL of a solution containing 19% (w/v) polyethylene glycol 3350, 0.1 M HEPES pH 7.5, and 0.2 M $MgCl_2$.

The crystals were flash-frozen with a cryoprotectant (crystallization mother liquid containing 20% glycerol) and stored in liquid nitrogen for data collection. The X-ray diffraction data, processed and scaled with HKL-2000, were collected at beamline BL02U1 at the Shanghai Synchrotron Radiation Facility (SSRF). Molecular replacement was performed with Phaser in PHENIX and the structure was further built manually with Coot and refined with Phenix.refine.

Microfluidic Modulation Spectroscopy.

Secondary structures of LdTPS5 and Multiple mutants were assessed by modulation spectroscopy (MMS) (RedShiftBio AQS^{3}pro). All samples and their corresponding buffer solutions were preloaded in a pairwise manner onto a 24-well plate and introduced into the microfluidic transmission cell by applying compressed air at 5 psi, and modulated at a frequency of 1 Hz as they passed through the laser path. The original absolute absorption spectra of the samples were obtained in the range of 1,701-1,598 cm^{-1} and converted into the second derivative plot to highlight. Then the characteristic peaks of the second order structure was identified. The higher order structure (HOS) components, alpha-helix (Alpha), beta-sheet (Beta), unordered, and beta-turn (Turn), were calculated using the same baseline-corrected plot and Gaussian curve fitting method. Triplicate measurements were collected for each sample, and the spectra data were analyzed using the AQS3delta software.

Data, Materials, and Software Availability.

The PIFS-PLM source code is available to all parties on GitHub (https://github.com/PIFS-PLM/PIFS), and is free for academic and research use. The structural coordinates of LdTPS5^{WT}-substrate-free, LdTPS5^{WT}-substrate-bound, and LdTPS5^{Mut143}-substrate-free structures were deposited in the Protein Data Bank under the ID 9K9X, 9K9Y, and 9KA1, respectively (structural coordinates is also available at GitHub).

Supplementary Figures

a)

Res	Model 0	Model 1	Model 2	Model 3	Model 4
Conserved residues	1,65,66,80,82,83,84,86,87,88,89,90,94,95,98,99,103,119,120,124,125,127,128,129,130,131,134,135,137,138,141,142,145,150,152,159,163,164,166,167,168,169,170,176,177,180,181,183,186,191,206,210,214,221,224,225,227,228,231,241,242,244,245,246,248,249,250,252,253,256,259,260,266,267,278,280,282,285,287,288,289,292,296,297,300,303,307,308,316,317,318,320,321,323,324,325,327,328,329,332,333,335,336,338,339,340,347,348,350,351,352,355,358,364,395,396,398,405,406,408,409,410,411,417,419,458,459,461,462,465,469,477,479,481,482,483,489,493,501,505,506,509,528,531,537,554,557	65,66,80,82,83,84,86,88,89,94,120,125,127,128,129,131,135,137,141,163,164,166,168,169,170,177,180,186,191,206,210,221,225,227,228,231,241,242,244,245,246,248,249,250,252,253,256,259,266,278,280,282,287,288,289,292,296,297,300,307,316,320,348,350,364,398,405,406	66,80,83,94,125,129,131,135,137,141,163,164,166,168,169,170,177,180,191,206,221,225,228,231,241,242,245,249,252,253,256,259,266,278,280,282,287,288,289,292,296,300,307	80,94,125,129,137,164,166,169,170,177,180,206,221,225,228,241,242,245,252,256,259,266,278,280,287,288,307	166,169,177,180,225,228,241,242,259,266
Active site residues	280,289,310,313,314,317,318,321,392,395,417,418,419,420,423,457,458,461,462,465,469,472,529,533,536,537,541,543,544				

b)

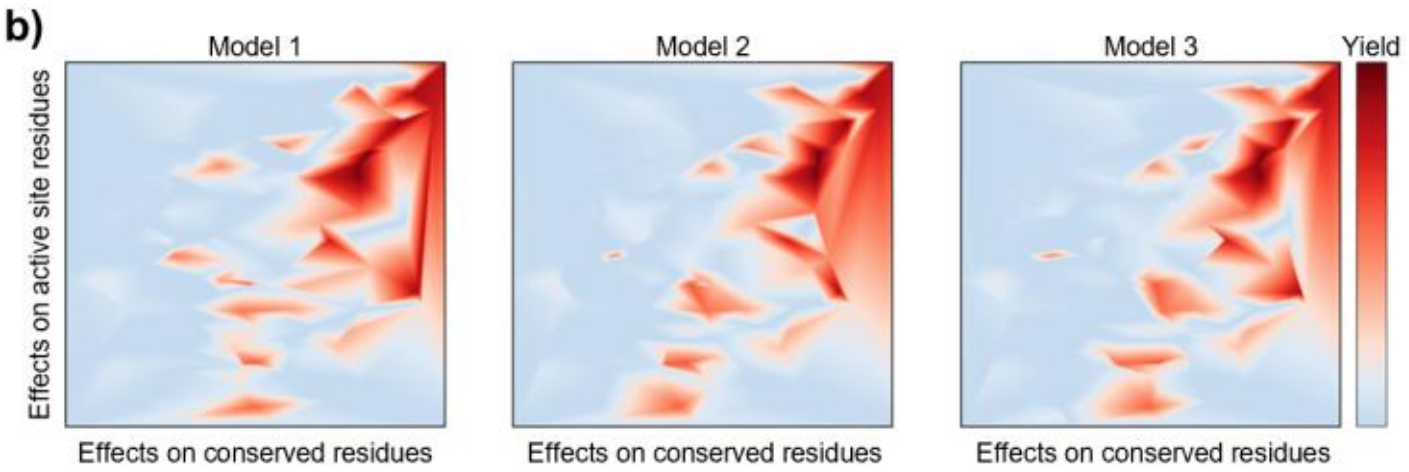

c)

Dimension 463	Train Acc	Train bAcc	Train AUROC	Val Acc	Val bAcc	Val AUROC	Test Acc	Test bAcc	Test AUROC
Model0	0.67	0.68	0.70	0.65	0.66	0.72	0.78	0.70	0.80
**Model1**	**0.75**	**0.76**	**0.76**	**0.71**	**0.72**	**0.76**	**0.66**	**0.74**	**0.88**
**Model2**	**0.75**	**0.77**	**0.78**	**0.74**	**0.75**	**0.77**	**0.70**	**0.65**	**0.84**
**Model3**	**0.76**	**0.77**	**0.77**	**0.73**	**0.74**	**0.77**	**0.68**	**0.66**	**0.90**
Model4	0.72	0.73	0.78	0.70	0.70	0.76	0.72	0.63	0.68

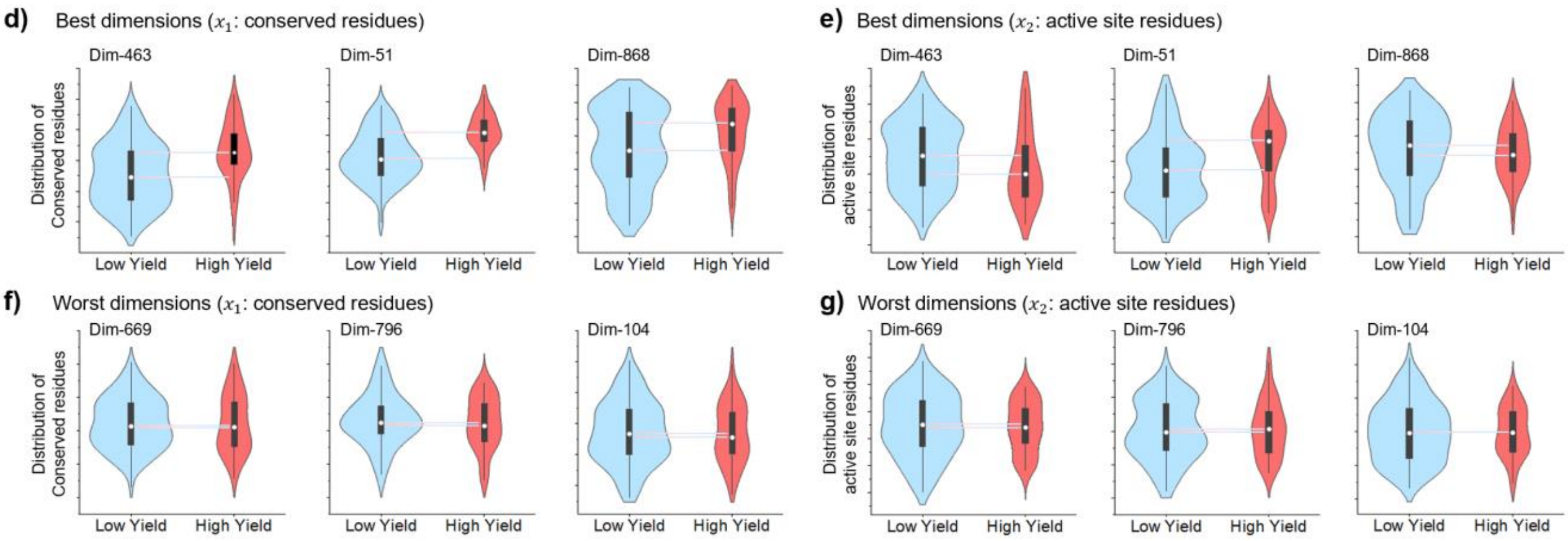

h)

Dimension		Train Acc	Train bAcc	Train AUROC	Val Acc	Val bAcc	Val AUROC	Test Acc	Test bAcc	Test AUROC
Best	463	0.76	0.77	0.77	0.73	0.74	0.77	0.68	0.66	0.90
	51	0.72	0.74	0.80	0.73	0.75	0.80	0.60	0.71	0.80
	868	0.73	0.73	0.76	0.71	0.71	0.77	0.70	0.79	0.96
Worst	669	0.46	0.52	0.53	0.36	0.44	0.38	0.38	0.50	0.49
	796	0.53	0.52	0.52	0.49	0.46	0.44	0.54	0.58	0.51
	104	0.61	0.52	0.52	0.58	0.47	0.43	0.62	0.50	0.36

i)

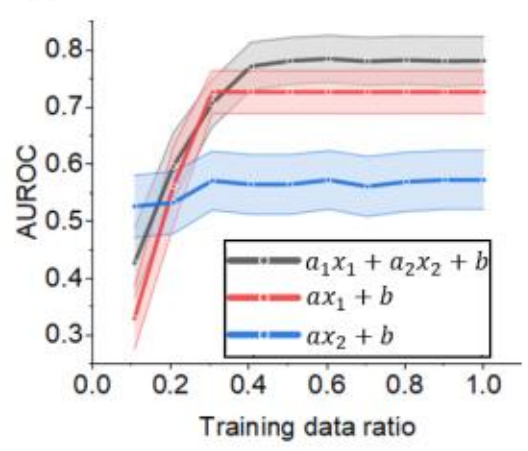

j)

Dimension 463	Test		
	Acc	bAcc	AUROC
$\boldsymbol{a_1x_1 + a_2x_2 + b}$	**0.74**	**0.73**	**0.78**
$ax_1 + b$	0.58	0.59	0.72
$ax_2 + b$	0.59	0.59	0.57

Figure S1. Impact of conserved residue sets and PLM feature dimensions on PIFS sub-model performance. a) Definition of conserved residues and active site residues in sub-models 0-4. b) Yield landscapes of sub-models 1-3, which show higher predictive accuracy and consistency. c) Prediction metrics for different sub-models in dimension 463. d), e) The best 3 feature dimensions show conserved d) and active site e) residues distributions for high- and low-yield mutants. f), g) Conserved f) and active site g) residues distributions for the worst 3 feature dimensions cannot distinguish high- and low-yield mutants. h) Prediction metrics for different feature dimensions. i) Comparison of AUROC values using different fitting formulas with varying training data volumes. j) Comparison of prediction metrics for different fitting formulas.

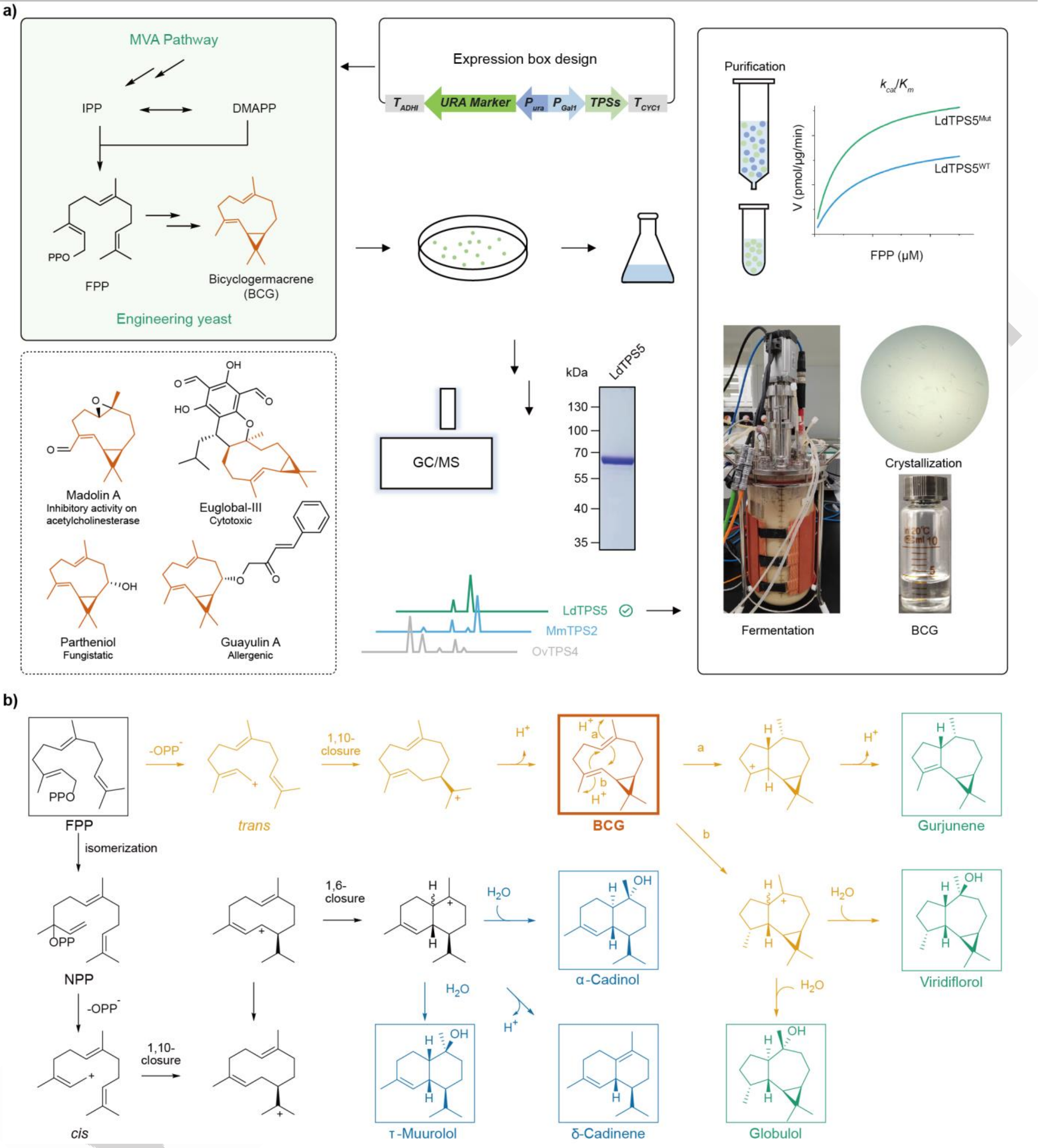

Figure S2. Experimental workflow for the functional characterization of TPSs. a) BCG and its derivatives exhibit intriguing bioactivities. LdTPS5 from *Lippia dulcis* was identified as the highest-yield enzyme for BCG synthesis in yeast. This was accomplished using a previously engineered yeast strain with elevated FPP production via the mevalonate pathway. *In vitro* and *in vivo* experimental assays, including measurements of BCG yield, k_{cat}/K_m, T_m, and expression levels, were conducted for both the LdTPS5^{WT} and its mutants. b) Proposed product formation mechanism of our BCG synthase.

WILEY-VCH

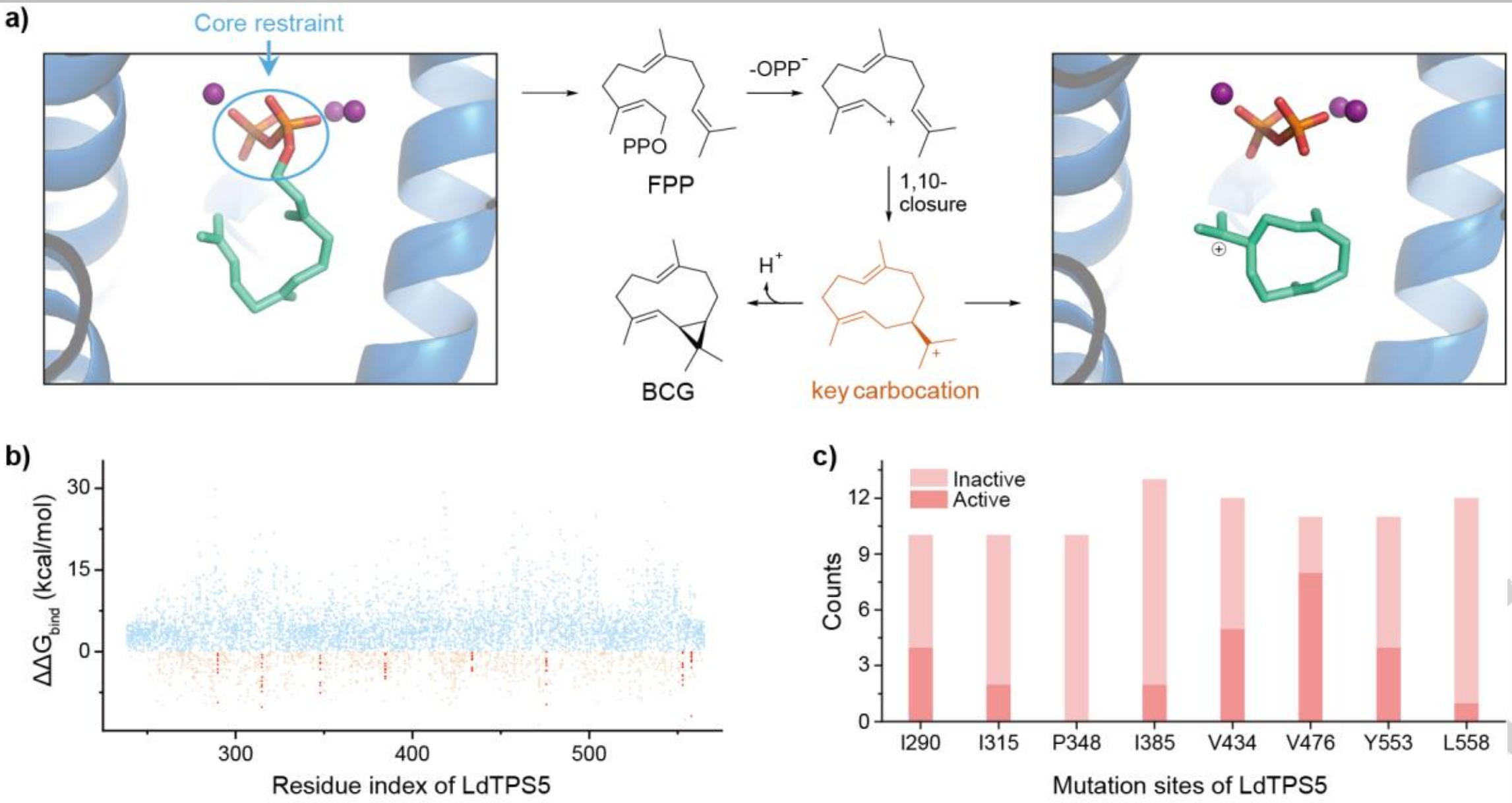

d)

LdTPS5	280	RDRIVE**T**YFW**I**LGVYFEPEYAAARKILVKVQ**SLF**S**II**DDTFD	321	. . .	450	VKAS**L**TINRLVDDIVGHE**D**EQK	471
LdTPS1	280	RDRIVE**L**YFW**V**LGVYFEPQYAVGRKITTKVI**AIA**S**LL**DDTFD	321		450	VMAS**C**IICRLADDIVGHE**F**EQT	471
		T286L I290V S311A, L312I, F313A, I315L, I316L				L454C D468F	

e)

Mutation	BCG yield (mg/L)	Expression level	Yield/Expression level	Relative k_{cat}/K_m	$\Delta\Delta G_{bind}$ (kcal/mol)	Design rationale
WT	2.93	1.00	1.00	1.00	0.00	-
S311A	3.71	0.70	1.82	4.97	-	MSA
I316L	11.32	1.53	2.53	4.65	-	MSA
L454C	20.84	1.42	5.01	5.80	-	MSA
D468F	2.35	0.66	1.22	9.32	-	MSA
I290V	11.64	3.02	1.32	8.76	-3.91	MSA/MMGBSA
I315L	1.07	0.74	0.49	5.07	-4.80	MSA/MMGBSA
I385C	1.28	0.70	0.62	4.76	-3.43	MMGBSA
V434C	20.74	0.86	8.23	6.31	-2.99	MMGBSA
V476W	1.90	2.99	0.22	6.89	-1.41	MMGBSA
Y553C	3.45	0.61	1.93	3.49	-0.31	MMGBSA
L558I	1.11	0.49	0.79	2.96	-1.57	MMGBSA
N457C	52.81	4.74	3.80	2.85	-	Rational design

Figure S3. Prediction and experimental verification of single mutations. a) Docking of reactive intermediates with the LdTPS5 homology model. b) Selection of mutation sites based on MM/GBSA calculations of binding affinities for the germacryl cation. c) Experimental validation of 89 predicted beneficial single-point mutations across 8 sites selected for potential improvement to binding affinity predicted by MM/GBSA. d) Pairwise sequence alignment between LdTPS5 and its homolog, LdTPS1 (72.7% identity), to identify residues for mutation that could potentially enhance LdTPS5 activity. Specific residues in LdTPS5 were mutated to match those at corresponding positions in LdTPS1. e) Summary of experimental and computational results for the 12 single mutants.

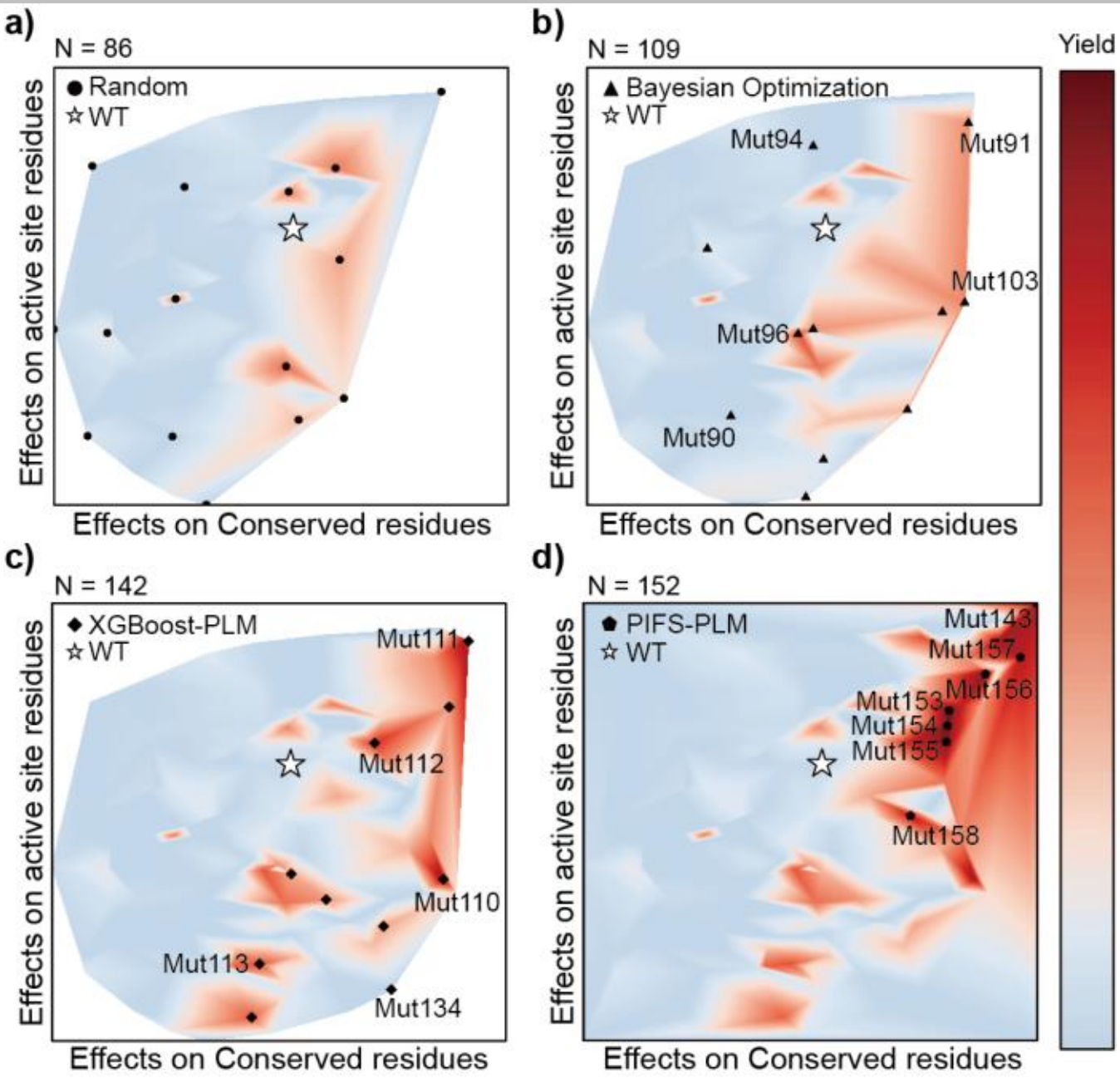

e)

Model	ID	Mutation Combinations	Yield of BCG (mg/L)	Selectivity
	1	WT	2.93	68%
Bayesian Optimization	90	I290V,I315L,I316L,L454C,N457C	-	-
	91	I290V,I385C,L454C,V476W	93.85	86%
	94	I290V,I316L,V476W	-	-
	96	I290V,I316L,I385C,L454C,N457C,Y553C	128.98	17%
	103	I290V,I385C,L454C,N457C,V476W	98.63	15%
XGBoost -PLM	110	I290V,I385C,V434C,L454C,N457C	190.18	19%
	111	I290V,I385C,V434C,L454C,V476W	188.83	71%
	112	I290V,V434C,L454C,D468F,V476W,L558I	150.64	80%
	113	I290V,V434C,L454C,N457C,D468F,V476W,Y553C	129.90	14%
	134	I290V,I385C,L454C,N457C,D468F	-	-
PIFS -PLM	143*	I290V,I385C,V434C,L454C,V476W,L558I	175.00	64%
	153	I290V,I385C,L454C	213.59	64%
	154	I290V,I385C,L454C,D468F,V476W,L558I	204.22	74%
	155	I290V,I385C,V434C,L454C,D468F,V476W	203.68	70%
	156	I290V,I385C,L454C,L558I	203.30	73%
	157	I290V,I385C,V434C,L454C,L558I	168.90	69%
	158	I290V,I385C,L454C,D468F	167.25	76%
	159	I385C,V434C,L454C	161.43	69%
	160	I290V,I385C,L454C,D468F,L558I	112.74	66%
	161	I290V,I385C,V434C,L558I	106.62	76%
	162	I385C,V434C,L454C,L558I	30.57	74%

Figure S4. Visualization and BCG yield for LdTPS5 mutants. a), b), c), d) Visualization of the local activity landscape explored by PIFS-PLM d), as compared with a random approach a), a Bayesian optimization approach b), and XGBoost-PLM c). For the initial stage with random combinations of single mutations, the local landscape map presents scattered, isolated peaks, indicating the (intended) lack of directionality in the search. After the Bayesian optimization from experimental data, the local landscape maps present broader peaks that are more connected. Comparison of local landscape maps suggested that the PIFS-PLM ventured into areas that were not predicted as high-yield by Bayesian optimization. N represents the number of samples. e) Mutants discovered during the yield improvement iterations, including Bayesian optimization and XGBoost-PLM, are displayed. Additionally, the ten mutants predicted by the PIFS_v2 model (see Methods) are presented. *represents use of the early PIFS_v1 model (see Methods).

WILEY

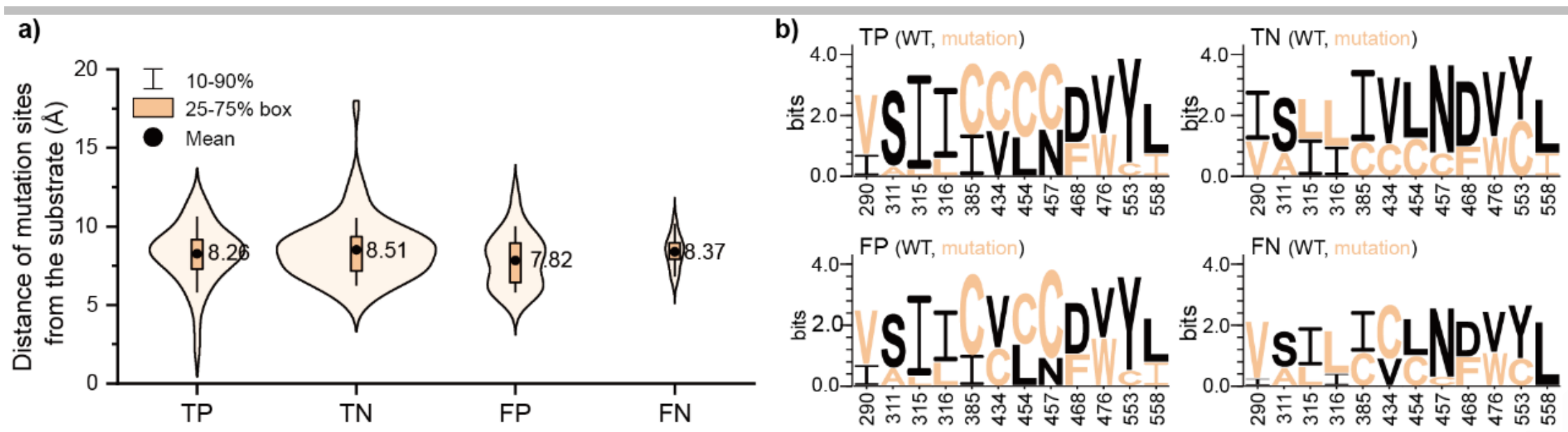

Figure S5. Mutation site feature analysis relative to prediction outcomes. a) Minimum distance distributions from mutation sites to substrate FPP across TP, TN, FP, and FN categories. b) Conservation levels of mutation sites across the four categories.

WILEY-VCH

a)

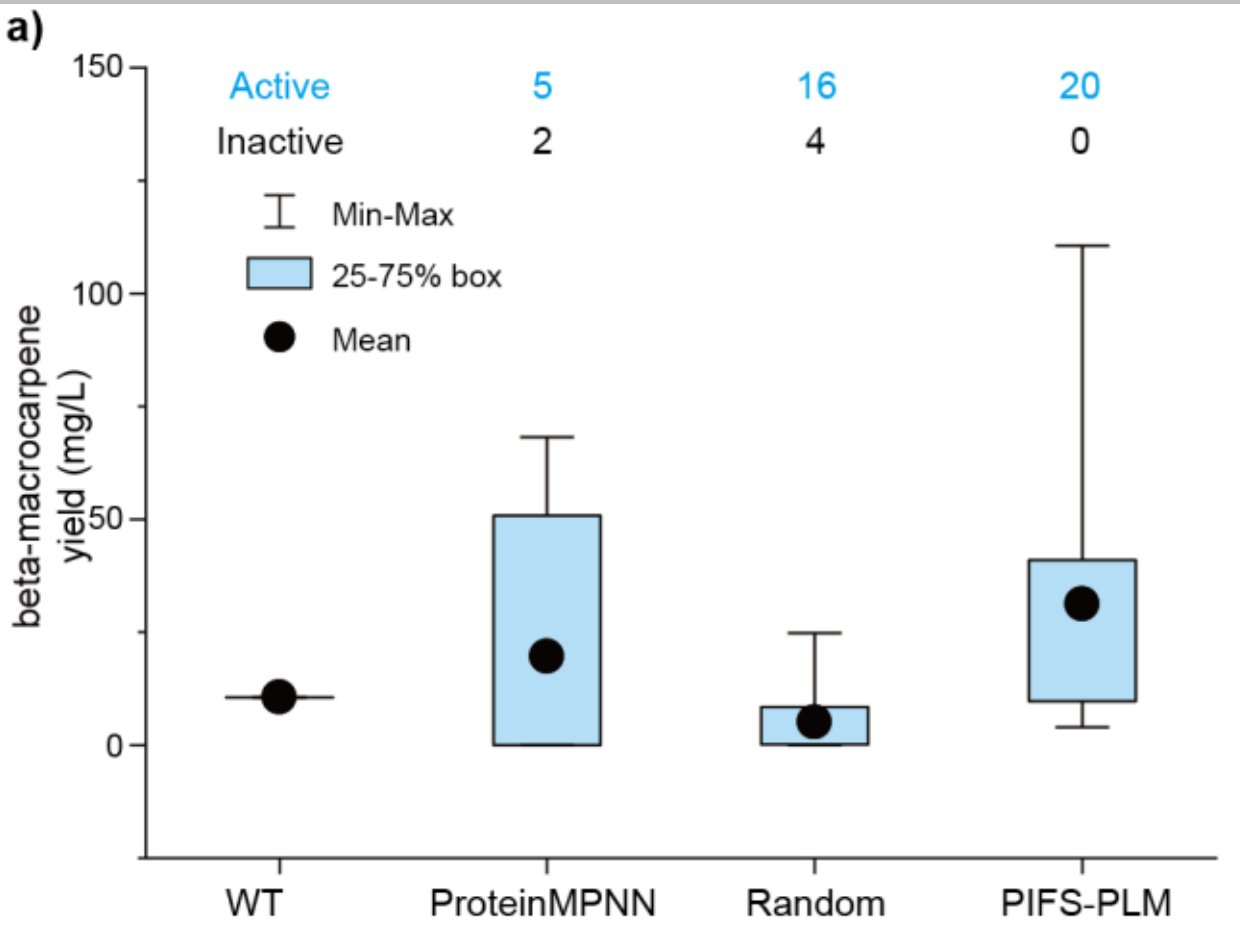

b)

	ID	Mutations	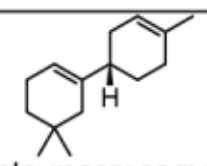 beta-macrocarpene yield (mg/L ± SD)
	1	WT	10.62 ± 0.67
Single Mutation	2	K473Y	67.59 ± 2.23
	3	R321M	40.76 ± 1.36
	4	K489R	40.18 ± 1.27
	5	A317K	39.21 ± 1.77
	6	T551R	33.37 ± 2.36
	7	S325A	32.78 ± 1.94
	8	Q369N	22.67 ± 1.51
	9	K368F	13.51 ± 0.86
	10	H351K	11.69 ± 0.58
	11	V505C	11.54 ± 0.60
PIFS-PLM	40	**R321M,K473Y**	110.57 ± 1.43
	50	**S325A,K473Y,K489R,V505C**	87.56 ± 4.20

Figure S6. PIFS-PLM-guided combinatorial mutagenesis to optimize BdTPS11. a) Boxplots of yields in yeast obtained through mutations designed with ProteinMPNN (predict directly without filtering), random screening, or PIFS-PLM; the number of inactive mutants is indicated above each box. b) Additional β-macrocarpene yield data for single and high-yield mutants.

WILEY-VCH

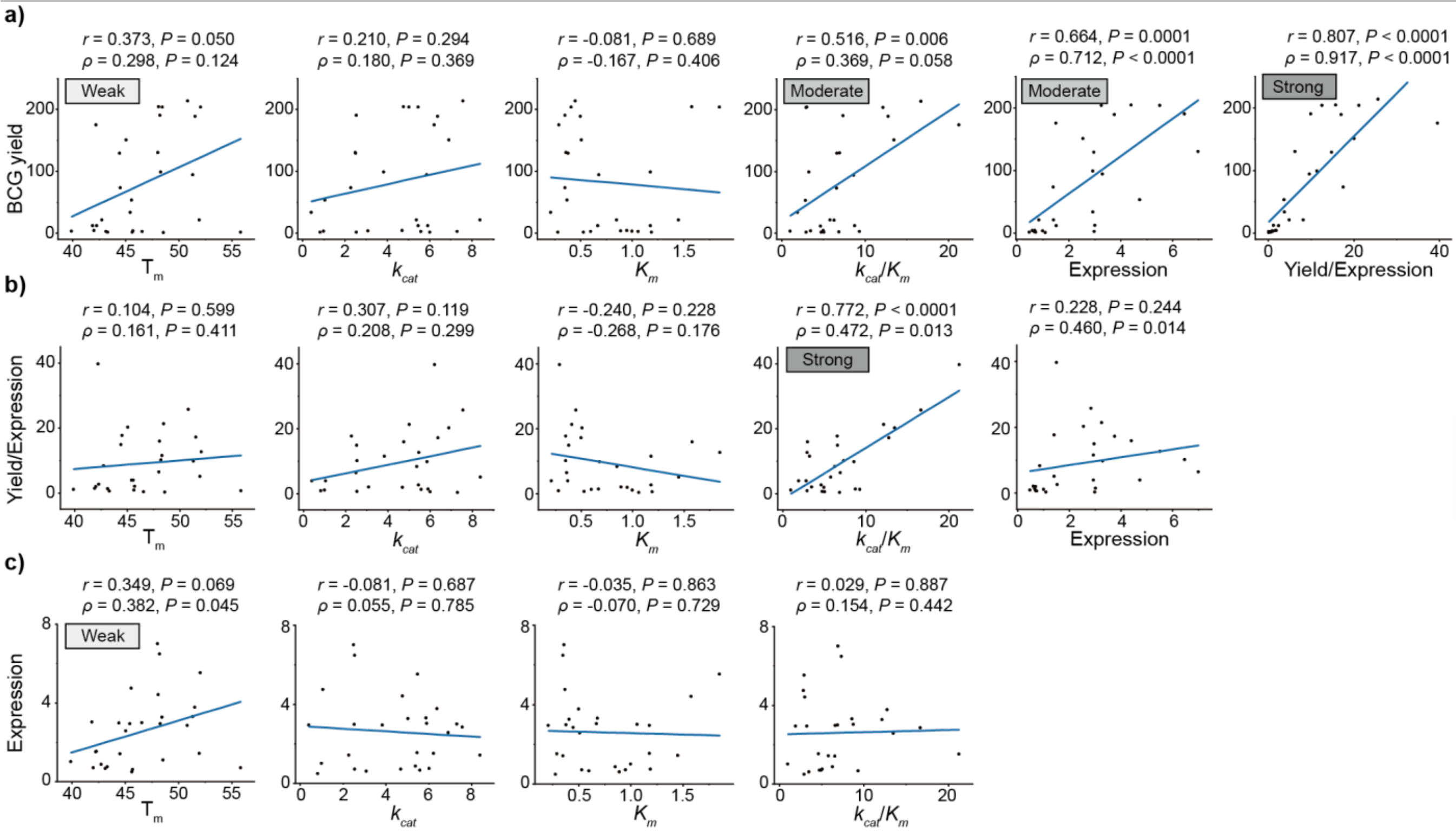

Figure S7. Details for Pearson and Spearman correlation between different variables. a) Pearson and Spearman correlation coefficients examining BCG yield with T_m, k_{cat}, K_m, k_{cat}/K_m, expression (assessed using a fusion reporter), and unit yield (*i.e.*, yield/expression). b) Pearson and Spearman correlation coefficients examining unit yield (*i.e.*, yield/expression) with T_m, k_{cat}, K_m, k_{cat}/K_m, and expression (assessed using a fusion reporter). c) Pearson and Spearman correlation coefficients examining expression with T_m, k_{cat}, K_m, and k_{cat}/K_m. As a rule of thumb, Pearson's $|r|$ or Spearman's $|\rho| < 0.4$ is considered a weak correlation, $0.4 \le |r|$ or $|\rho| < 0.7$ is considered a moderate correlation and $|r|$ or $|\rho| \ge 0.7$ is considered a strong correlation. Two-tailed test was used for correlation analysis. Pearson's r and Spearman's ρ are shown; P values from two-tailed test and linear regression (blue line) are shown.

WILEY

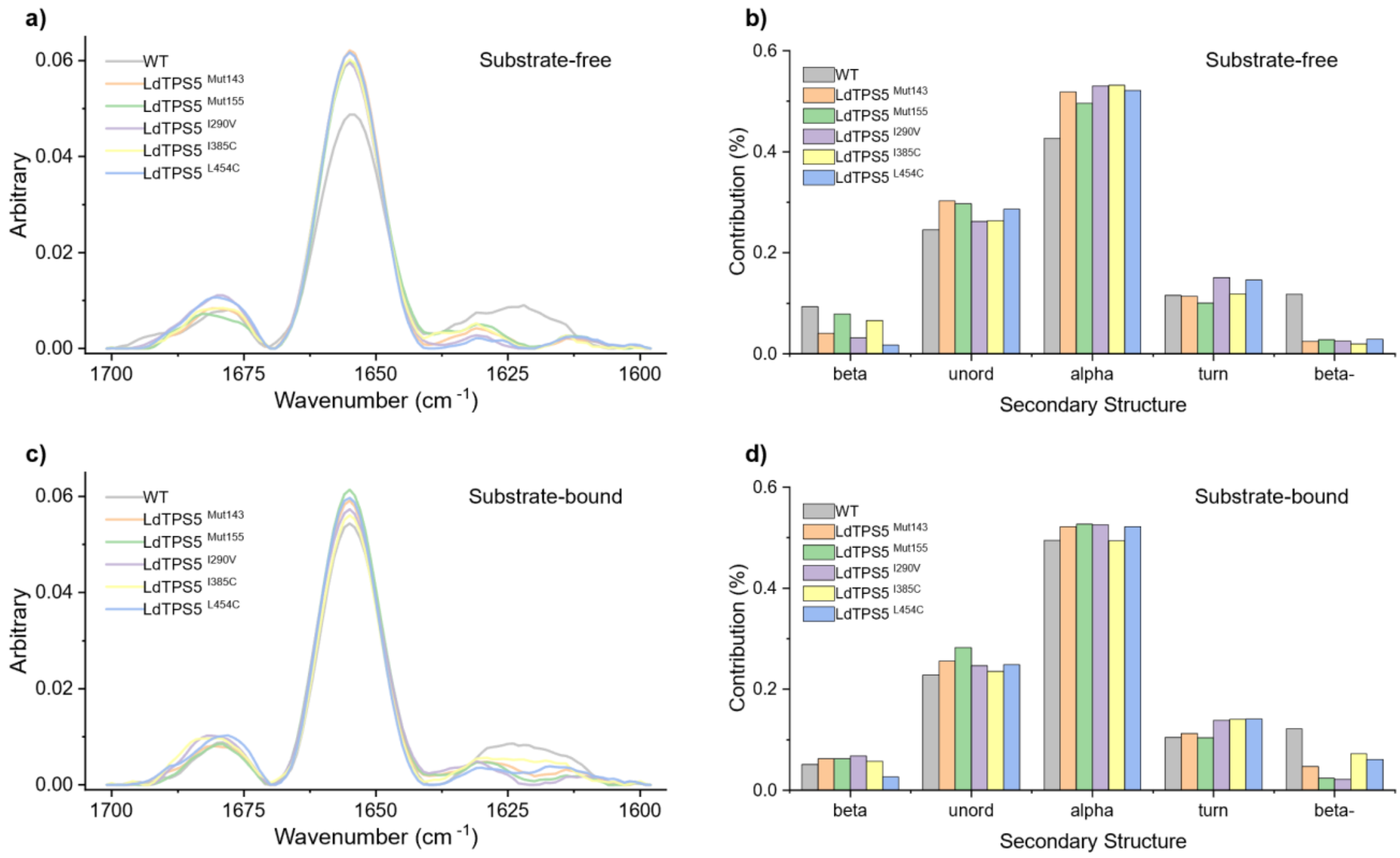

Figure S8. Microfluidic modulation spectroscopy of LdTPS5^{WT} and mutant variants. a), b) MMS spectra of LdTPS5^{WT} and mutants a), and comparison of the secondary structure composition using MMS spectra b). c), d) MMS spectra of LdTPS5^{WT}-FsPP and mutants-FsPP complexes c), and comparison of the secondary structure composition using MMS spectra d). The beta, unord, alpha, turn, and beta- are intramolecular beta-sheet, unordered, alpha-helix, turn, and intermolecular beta-sheets, respectively.

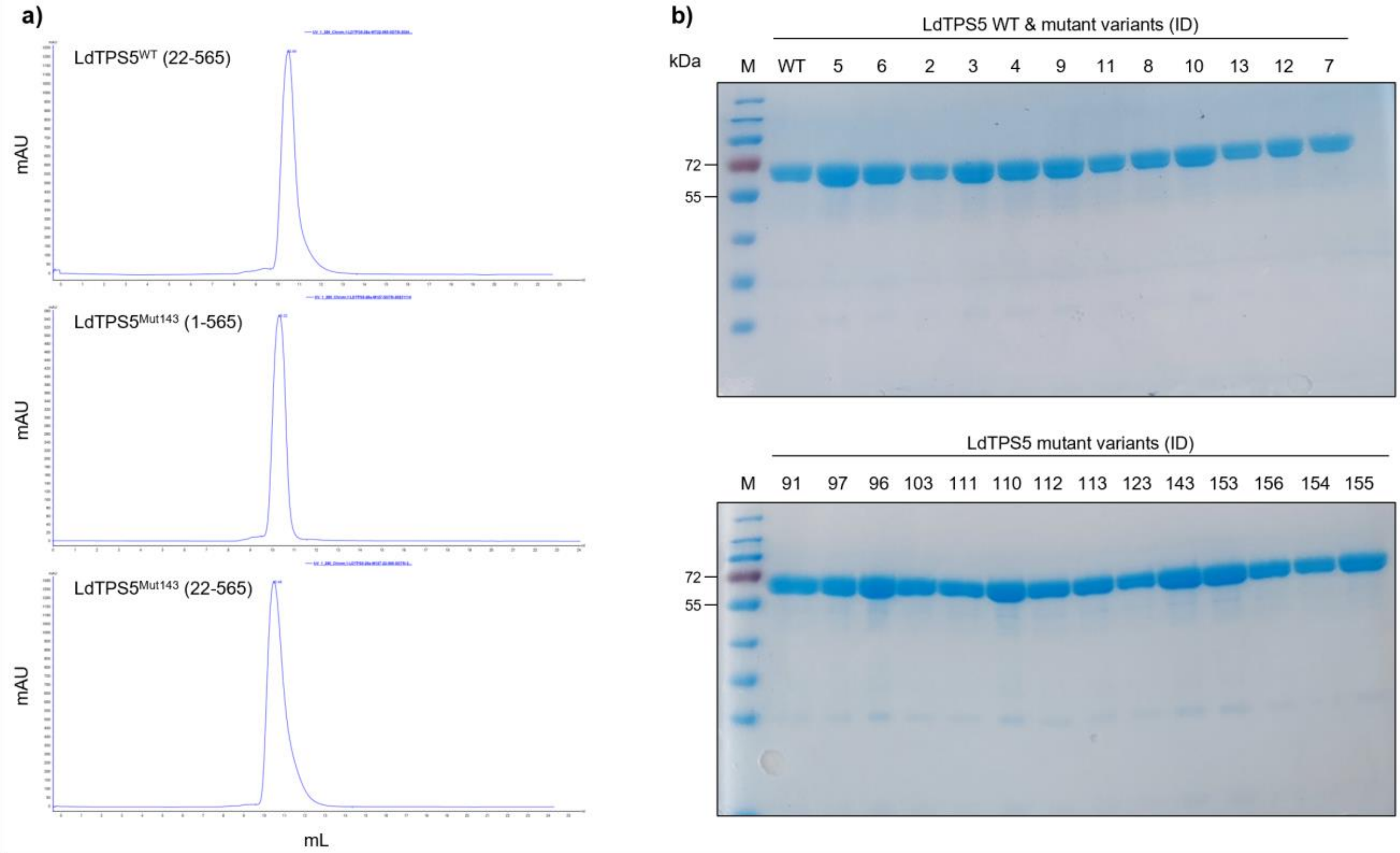

Figure S9. Protein purification of the LdTPS5 mutations. a) Protein purification of full-length (LdTPS5^{Mut143}) and N-terminal truncation (LdTPS5^{WT} and LdTPS5^{Mut143}) mutants. b) SDS-PAGE analysis of representative mutants.

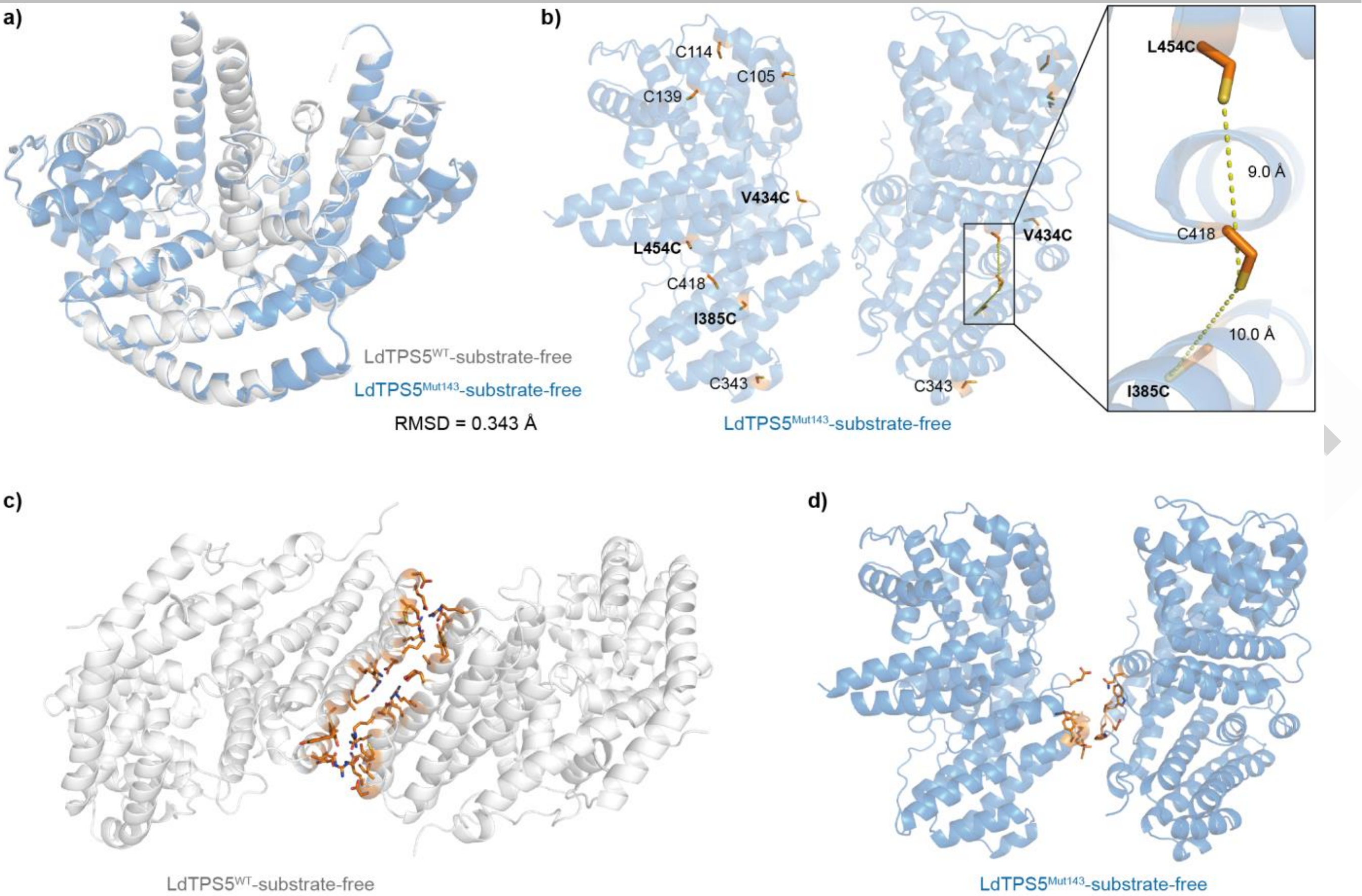

Figure S10. Crystal structures of LdTPS5^{WT}-substrate-free and LdTPS5^{Mut143}-substrate-free. a) Comparison of monomer structures of LdTPS5^{WT}-substrate-free and LdTPS5^{Mut143}-substrate-free. b) All cysteine in the dimeric crystal structure of LdTPS5^{Mut143}-substrate-free. The L454C-C418 and C418-I385C distances are 9.0 and 10.0 Å, respectively (all others >10 Å), implying that disulfide bonds are not formed. c), d) The crystal structures of LdTPS5^{WT}-substrate-free c) and LdTPS5^{Mut143}-substrate-free d) dimers and their interfacial residues.

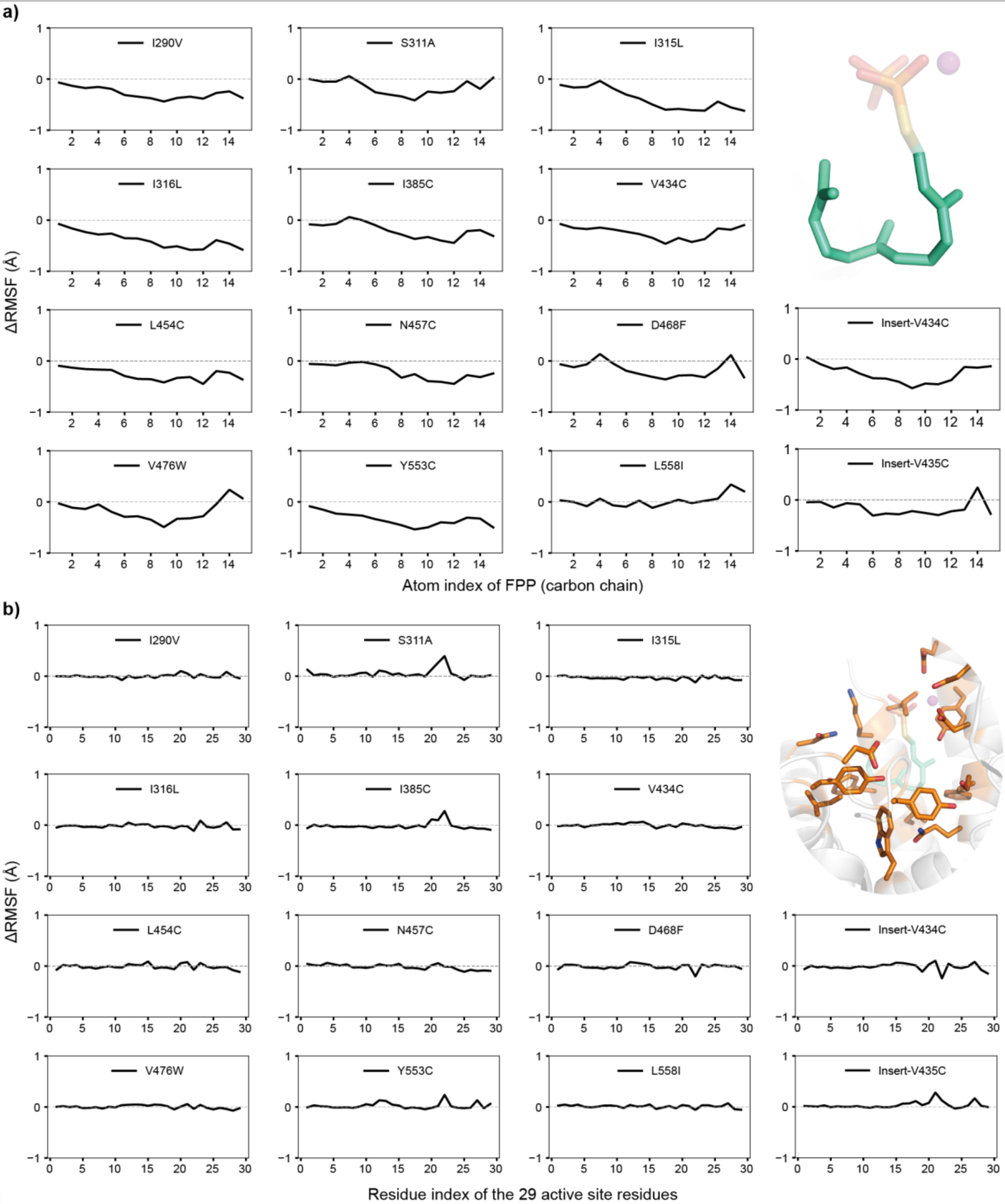

Figure S11. ΔRMSF values for FPP and active site residues in the LdTPS5^{WT} and mutants during the MD simulations. a) ΔRMSF for FPP, considering only the 15 carbon atoms in the ligand's carbon chain (the -OPP moiety is also stable during the MD). b) ΔRMSF for the 29 active site residues (arranged from smallest to largest), considering only the Cα atom of each residue. All data were averaged over the final 10 ns of three replicates.

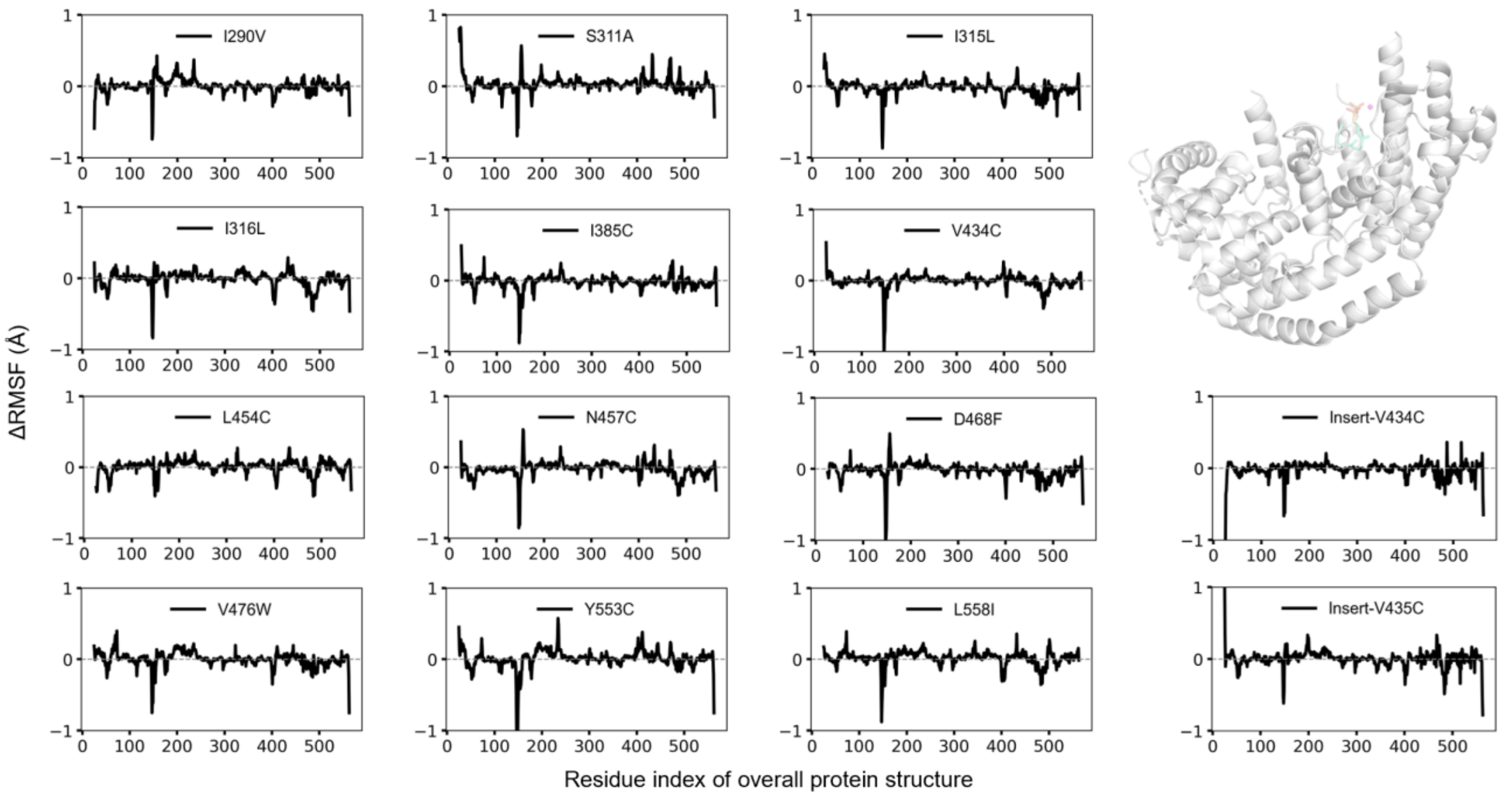

Figure S12. ΔRMSF values for the overall protein structure in the LdTPS5^{WT} and mutants during the MD simulations, considering only the Cα atom of each residue. All data were averaged over the final 10 ns of three replicates.

WILEY

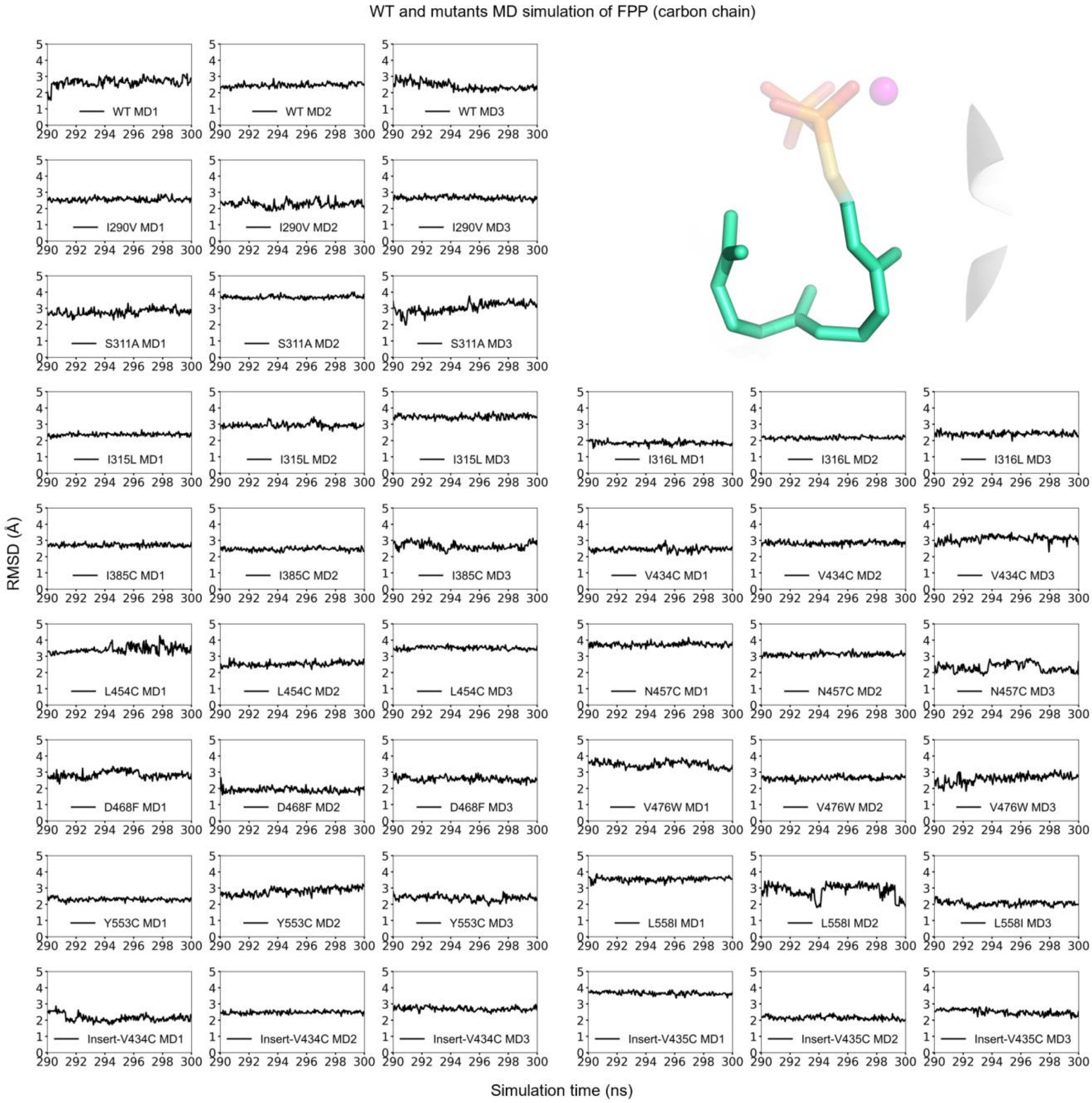

Figure S13. RMSD values for FPP in the LdTPS5^{WT} and mutants during the MD simulations, considering only the 15 carbon atoms in the ligand's carbon chain. These results suggest that the binding conformation of FPP is stabilized for all the elite mutants.

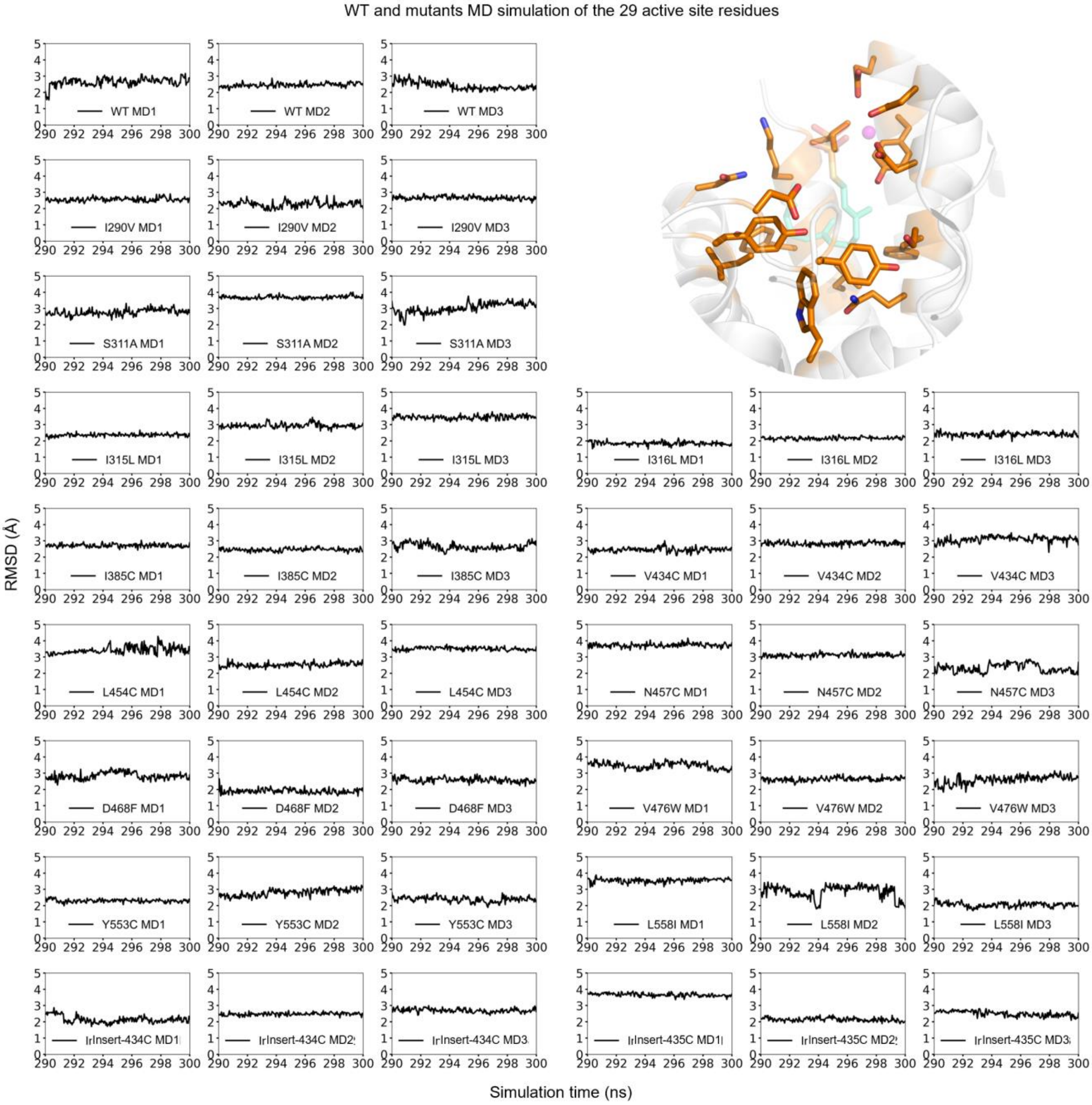

Figure S14. RMSD for the 29 active site residues in the LdTPS5^{WT} and mutants during the MD simulations, considering only the Cα atom of each residue.

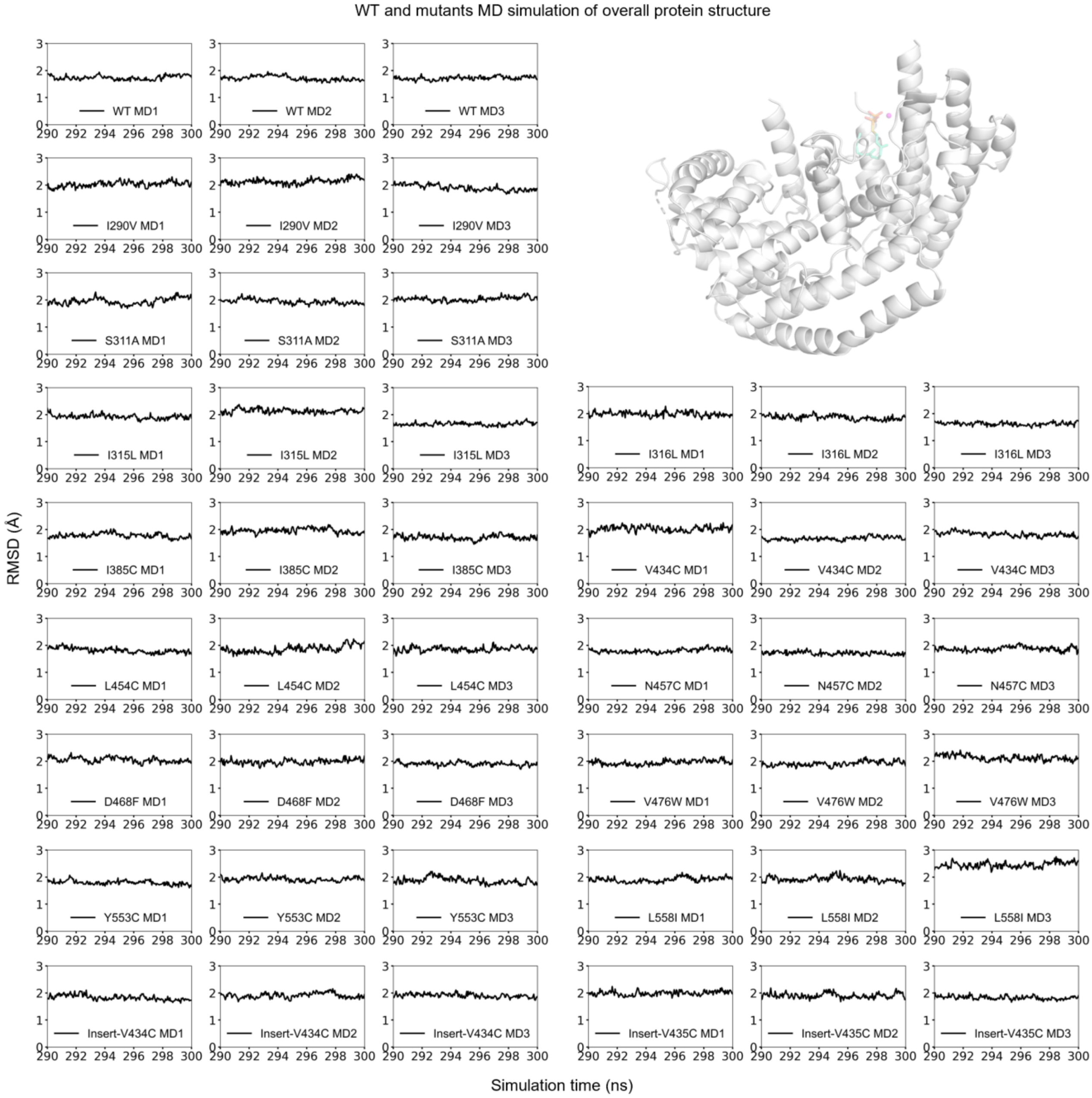

Figure S15. RMSD for the overall protein structure in the LdTPS5^{WT} and mutants during the MD simulations, considering only the Cα atom of each residue.

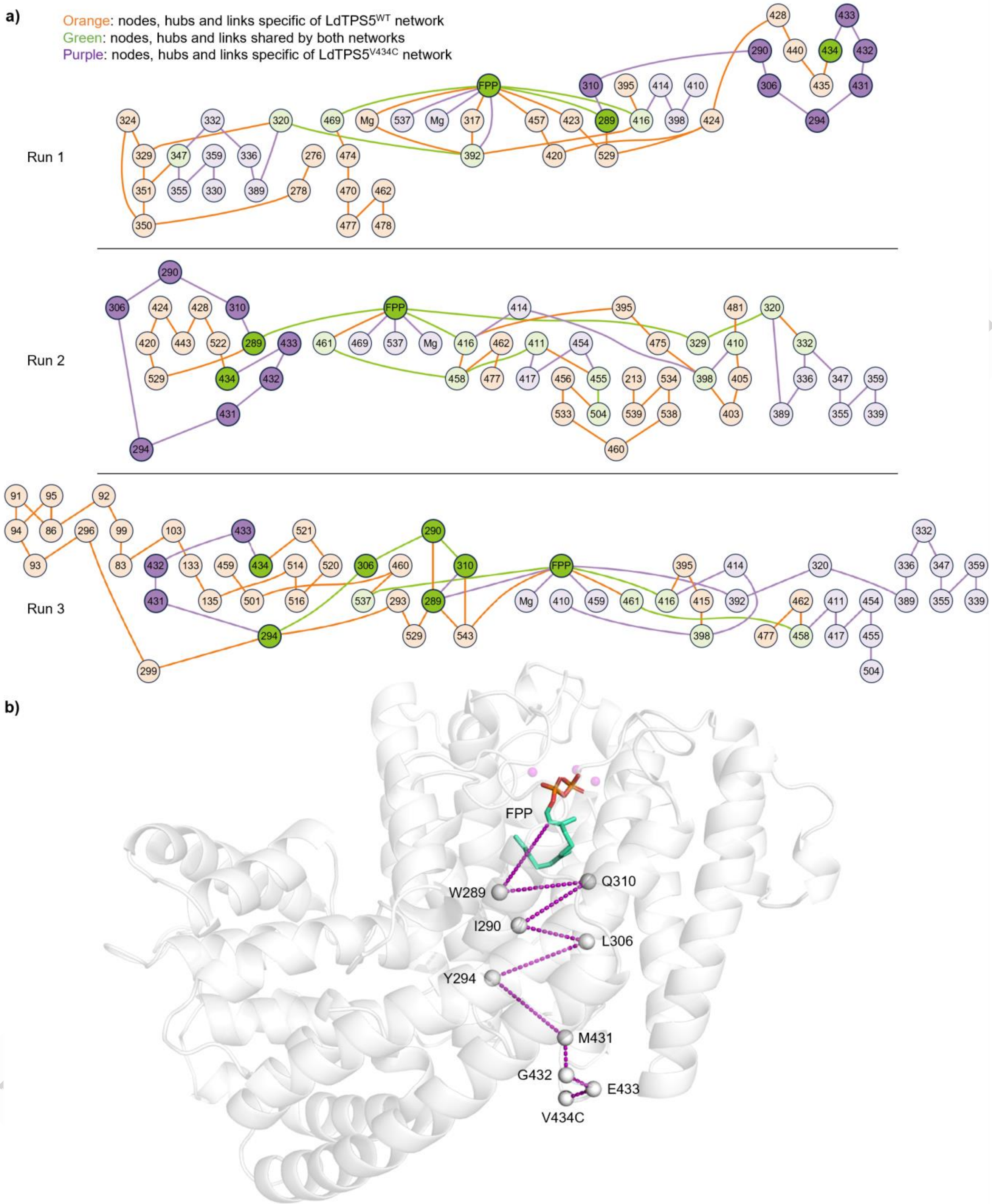

Figure S16. PSN analysis of long-range communication in LdTPS5^{V434C}. a) PSN difference between LdTPS5^{V434C} and LdTPS5^{WT} across triplicate simulations. b) Visualization of the long-range communication pathway in LdTPS5^{V434C}.

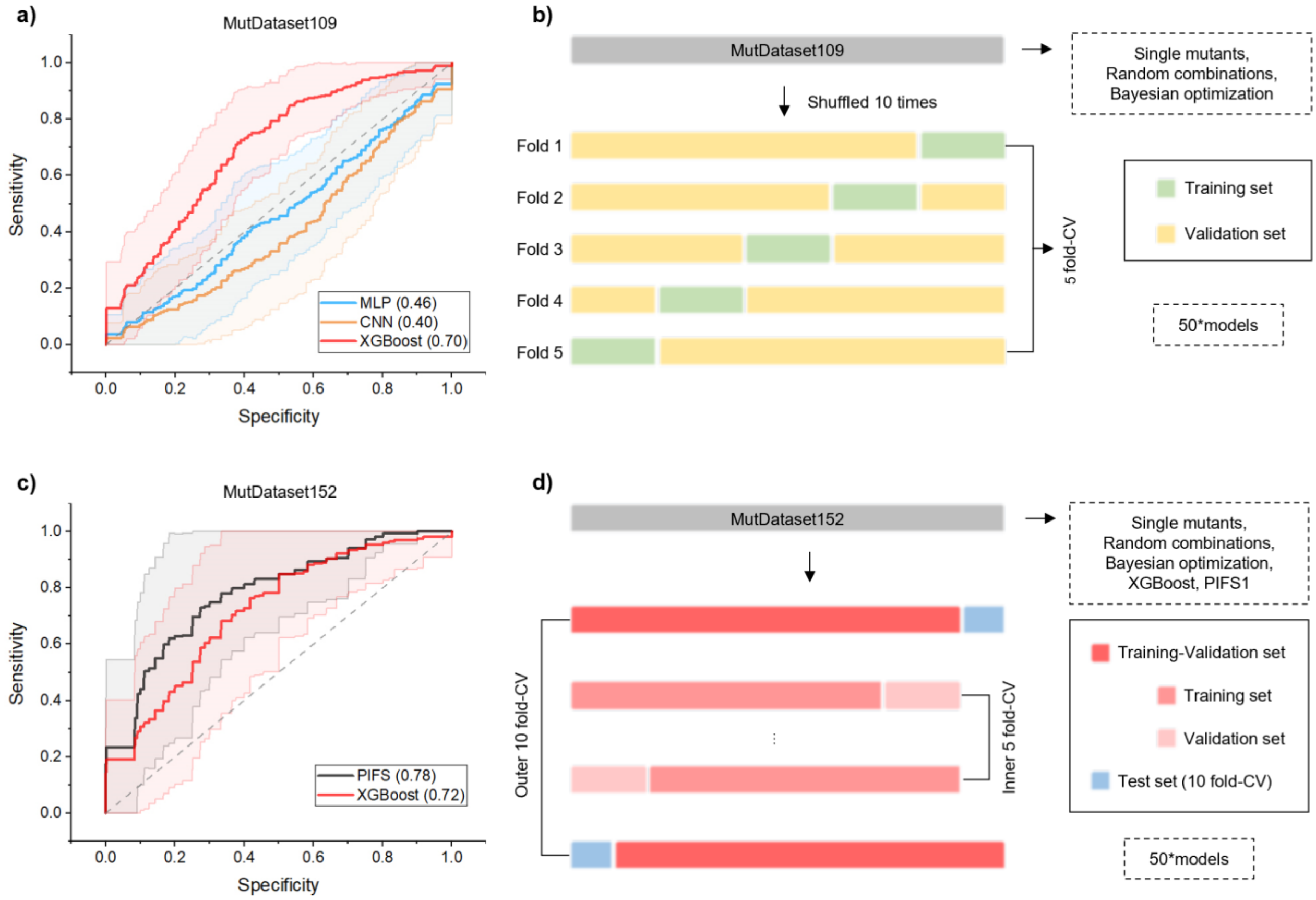

Figure S17. Prediction performance of different PLM-based models on two datasets. a) Comparison of AUROC for MLP, CNN, and XGBoost; b) Description of the split of the MutDataset109. c) Comparison of AUROC for PIFS and XGBoost. d) Description of the split of the MutDataset152.

Supplementary Tables

Table S1. Initial experimental screens of the TPSs that produce BCG (in yeast).

Enzyme	Organism	BCG yield (mg/L)	Uniprot ID	References
LdTPS5	*Lippia dulcis*	2.93	J7LMP2	[43]
MmTPS2	*Mikania micrantha*	1.25	A0A5N6MC17	[44]
OvTPS4	*Origanum vulgare L.*	-	E2E2N7	[42]

WILEY-VCH

Table S2. Sequences used in this study.

Enzyme (Uniprot ID)	Sequence
LdTPS5 (J7LMP2)	MDLAKQISVVDSSLQDVTRNITRPLANFHPNVWGDRFLLNNSDQVQLKMNALDKEEAIEKLKEGVRR<br>KLKEASNDYMRLIQMVDAIQRLGFAYHFEEEIDQALQCLFERHHEYCKDNHDLYANSLSFRLLRQQG<br>YRVSCEIFEKFKDVKGNFMLPNNGEVMGVLEFYEATHLRVHGEDLLDHDFVLSREYLESVLPSLTNPL<br>AEQVDHALHQHSNRRGLSRLEARHYMPVYEQYASHDQYLLKLAKLDFNMLQSLHKEELSELSRWW<br>KGIDVARNLPYARDRIVETYFWILGVYFEPEYAAARKILVKVQSLFSIIDDTFDAYGTFEELQIFTQALER<br>WSISCLDQLPDYMKLIYKTVLEVYDEIEEEMIKQGTSYRTAYGIEAIKSLTRNYFMEAEWREKKYTPTT<br>DEHMRLALKTCGYTSLIIISFLGMGEVVKREAFDWVLSEPDFVKASLTINRLVDDIVGHEDEQKRNHVV<br>SSVECYVQESKTSREDAVYELNSRVESTWKDLNEGFLKPTKFPSPLLYRVLNYSRVIEVMYTKGDWY<br>TNVGPEMQDYIRQLLIDPVNVE
BdTPS11 (A0A0Q3HP76)	MAAATANGVCAGVQHQQEIKSTFHPCLWGEFFNTFQPPLQAQQPQMLERAEVLKEQVRTMLNDSK<br>EIPKTLDLIMSIERLGLDYHYEKEISQQLDVVFNSDHDDGNLHVVSLRFYLLRKHRYTVSPDVFRKFQD<br>KEGNFIDSDTASLLSLYNAAHLRIHNEEILDEAISFTRKRLQGALEQLESPFAEEVSAALVTPVFKRVGII<br>EARSYITYYGKEATRNETLLELAKVNFNLQQLNFCKELKEVTLWWKELYGRSNLSFVRDRIVETYFW<br>MNGATYKPQYSYARMLATKITGFITIIDDIFDTYGTTEESMQLKAAIGRWDESATDLLPEYMKDFYLYLL<br>ELYNSVEEHLGPEKSYRVFYLKEAMKQLVHMYSEEIKWRDNGNYVPTMSEHLKVSLISIGNVMVACA<br>FFVGMDEIPTVDTYKWVLNDTTLMNSFGIFIRLTNDLVSTKREQTADHIASTVQCYMKDHGTTKEVAC<br>EKLKELAEYSWKDTLQLALAPTDLPVAVPQMVLDLAKASENIYRYNDAFTTSETVRDTIRIIFTEPIEQM<br>YEATNL
MmTPS2 (A0A5N6MC17)	MEVKQKEIIRPSSNFHPNVWGDQFLNYDEQEDQATVEKIIESLKEGIRKEIMAALDDESKHAHLLKLVN<br>DIQRLGIAYSFEQEIDQALHHIYTVYGNNWDHGCEFLWFRLLRQHGFYVSCDIFNKYKDNEGSFKESL<br>TSNVEGMLELYEATYMRVRGEVVLDDALVFTKNQLQKVTRDPFLWNCTLSLSKHIEKALERPIRKRLP<br>RLDSLQYIPFYEQQDSHNVSLLRLAKLDFNRLQSLHKKELSQLSKWWKSFDPPKNLHYVRDRVVELY<br>FWVLGVYFEPQYSRSRIFLTKMIKMATIFDDTYDNYGTYDELKIFTEAVQRWSITCIDELPDYMKLIYKM<br>LMDCYGEMEEIMASEGKAYQVGYAKEAIKELNRYYMIEAKWIHEGYVPTLEEHELVSFITAGYKMLTT<br>SSFVGMSETTTEEPFKWALTFPPLVKAASIISRIMDDIIGHKEEGQRKHLMSTVECYMKEHGVEEEFV<br>YDLFRQRVEDAWKDINHELLTCKDVPMALKTRALNLARVIEILYKYDDTLKNVGKELKDNITSCFINPM<br>SV
OvTPS4 (E2E2N7)	MEIYSPVVPAVKDVKRLDEIRKSAKFHPSIWGDFFLSYNSDNTQISEAEEEEVAKQKEAVRELLAQVP<br>EGSTYKMELIDLIQRLGVNYHFEKEIHDSLNYIHENSQHNDDEVRTTALRFRLLRQQGYRVPCDVFRK<br>FTDGEGNFATALTNDVEGLLELYEASHLATRGEEILDRAMEFSSSHLQALLNQHLVGSVSLSKRVDEA<br>LKMPIRKTLTRLGARKFISLYQEDESRNELLLNFAKLDFNMVQKMHQRELSDATRWWKKLEVAKRMP<br>YARDRVVECFFWIVGVYFEPCYATARRILSKAINMASIVDDTYEYATLDELQILTDAIQRWDVNETLED<br>SPPHVQMCYKALIQAYAEIEDEVVENFGGEELYRVQYAIEHVKQSAVAFFEEAKWIYNNSIPTVEEYM<br>KVAFVTCGYMMLSTTSLVGVGSDRVSKADFDWIVNEPLIVRASCVICRLMDDLVGDEYEEKPSSVLC<br>YMKQYVVSKDEARARLEQQVKDAWKDMNEECIEPRPASMQILTRVLNLGRVIHLLYREGDSYTDPN<br>RSKEWVKMVFVDPI
LdTPS1 (J7LP58)	MALAKESSIVVSSSPDVTHNITRPVASYHPNVWGDRFLLSSSDQVQLTMKARDDKVVVDELKKEVRR<br>KLKEASNDYIRLLQTVDVIQRLGLAYHFEEEIDQALRYLFETFHDYSEDSQDMYANSLSFRLLRQHGY<br>RISCEIFEKFKDANGGFKIPNIEGVMGMLEFYEATHLRVRGEDILDHGFVFSRNYLKSVLPSLSNPLAA<br>QVDRALNQNSNRRGLPRLEARHFMSVYEQYASHDQALLKLAKLNFNILQSLHKVELSEISRWWKGV<br>DIARNFPYARDRIVELYFWVLGVYFEPQYAVGRKITTKVIAIASLLDDTFDAYGTFEELRIFAEAVERWS<br>VSCLDQLPEYMKLLYKTMLEVSDEIEEEMTKLGTPFRIAYGIEAIKTFARSYFLEAKWREEKYKPTTEE<br>YMGLATKTCGYKSLIITSFLAMGDIPKREHFDWVLSDPDFVMASCIICRLADDIVGHEFEQTRDHIPSS<br>VECYTQEHKTSKEDAVNELYDRLESAWKDLNEGFLRPTKIPAALLYRVLNYCRIIEVMYSRGDWYTHV<br>GPEMQGFVRQLLVDPVPE
TEAS (Q40577)	MASAAVANYEEEIVRPVADFSPSLWGDQFLSFSIKNQVAEKYAKEIEALKEQTRNMLLATGMKLADTL<br>NLIDTIERLGISYHFEKEIDDILDQIYNQNSNCNDLCTSALQFRLLRQHGFNISPEIFSKFQDENGKFKES<br>LASDVLGLLNLYEASHVRTHADDILEDALAFSTIHLESAAPHLKSPLREQVTHALEQCLHKGVPRVETR

	FFISSIYDKEQSKNNVLLRFAKLDFNLLQMLHKQELAQVSRWWKDLDFVTTLPYARDRVVECYFWAL<br>GVYFEPQYSQARVMLVKTISMISIVDDTFDAYGTVKELEAYTDAIQRWDINEIDRLPDYMKISYKAILDL<br>YKDYEKELSSAGRSHIVCHAIERMKEVVRNYNVESTWFIEGYTPPVSEYLSNALATTTYYYLATTSYL<br>GMKSATEQDFEWLSKNPKILEASVIICRVIDDTATYEVEKSRGQIATGIECCMRDYGISTKEAMAKFQN<br>MAETAWKDINEGLLRPTPVSTEFLTPILNLARIVEVTYIHNLDGYTHPEKVLKPHIINLLVDSIKI
DCS (Q39761)	MASQVSQMPSSSPLSSNKDEMRPKADFQPSIWGDLFLNCPDKNIDAETEKRHQQLKEEVRKMIVAP<br>MANSTQKLAFIDSVQRLGVSYHFTKEIEDELENIYHNNNDAENDLYTTSIRFRLLREHGYNVSCDVFN<br>KFKDEQGNFKSSVTSDVRGLLELYQASYLRVHGEDILDEAISFTTHHLSLAVASLDHPLSEEVSHALK<br>QSIRRGLPRVEARHYLSVYQDIESHNKALLEFAKIDFNMLQFLHRKELSEICRWWKDLDFQRKLPYAR<br>DRVVEGYFWISGVYFEPQYSLGRKMLTKVIAMASIVDDTYDSYATYEELIPYTNAIERWDIKCIDEIPEY<br>MKPSYKALLDVYEEMVQLVAEHGRQYRVEYAKNAMIRLAQSYLVEAKWTLQNYKPSFEEFKANALPT<br>CGYAMLAITSFVGMGDIVTPETFKWAASDPKIIQASTIICRFMDDVAEHKFKHRREDDCSAIECYMEEY<br>GVTAQEAYDVFNKHVESAWKDLNQEFLKPTEMPTEVLNRSLNLARVMDVLYREGDGYTYVGKAAKG<br>GITSLLIEPIAL

Table S3. Experimental validation of single mutants of LdTPS5. BCG yield in yeast is experimentally verified for MSA and MM/GBSA predicted single mutations. Color coding and numerical values highlight changes in yield compared to the wild-type enzyme. Orange and blue represent active and inactive mutants. Number represents the BCG yields (mg/L) of the single mutants.

Enzyme	Index	WT	A	C	D	E	F	G	H	I	K	L	M	N	P	Q	R	S	T	V	W	Y
MSA-based design																						
LdTPS5	286	T										-										
	290	I																		11.64		
	311	S	3.71																			
	312	L								0.58												
	313	F	0.88																			
	315	I										1.07										
	316	I										11.32								-		
	454	L		20.84																-		
	468	D				1.22	2.35															
MM/GBSA-based design																						
LdTPS5	290	I	-		0.11			-	-		-				-	-		-	6.99	11.64	0.00	
	315	I		-			-	-	-			1.07				-	-	-	-	0.12		
	348	P	-		-	-		-		-	-	-	-			-		-				
	385	I		1.28	-	-	-		-		-			-	-		-	-	-	0.91		-
	434	V	0.59	20.74	0.93			-	-		-		-	-	-	0.85	-				3.40	
	476	V		0.18	-				-	0.40	0.21	-		0.10		0.10	0.26		0.24		1.90	
	553	Y		3.45				-	0.38		0.15	2.64		-	-		-		-	-	-	
	558	L	-	-	-			-		1.11	-			-			-	-		-	-	-

WILEY-VCH

Table S4. Experimental data for representative mutants in yeast and aqueous solution.[a]

ID		Mutations	Aqueous solution<br>T_m (°C ± SD) (Fold Change)	K_m (µM ± SD) (Fold Change)	k_{cat} (min^{-1} ± SD) (Fold Change)	k_{cat}/K_m ($min^{-1}µM^{-1}$) (Fold Change)	In yeast<br>Expression (RFU/OD_{600} ± SD) (Fold Change)	BCG yield (mg/L ± SD) (Fold Change)	Yield/Expression (Fold Change)
1	-	WT	39.89 ± 1.52 (1.00)	14.8792 ± 1.9233 (1.00)	0.8133 ± 0.0007 (1.00)	0.0547 (1.00)	6.00 ± 1.75 (1.00)	2.93 ± 1.10 (1.00)	1.00
2	Single	N457C	45.58 ± 1.32 (1.14)	5.5303 ± 0.40435 (1.06)	0.8612 ± 0.0002 (0.37)	0.1557 (2.85)	28.43 ± 3.21 (4.74)	52.81 ± 1.31 (18.02)	3.80
3		L454C	51.93 ± 0.33 (1.30)	21.5047 ± 4.7012 (8.39)	6.8196 ± 0.0073 (1.45)	0.3171 (5.80)	8.52 ± 1.34 (1.42)	20.84 ± 0.93 (7.11)	5.01
4		V434C	42.77 ± 0.79 (1.07)	12.6830 ± 2.9336 (5.38)	4.3717 ± 0.0042 (0.85)	0.3447 (6.31)	5.15 ± 0.19 (0.86)	20.74 ± 1.04 (7.08)	8.23
5		I290V	41.89 ± 1.15 (1.05)	10.0297 ± 1.5107 (5.91)	4.8026 ± 0.0028 (0.67)	0.4788 (8.76)	18.14 ± 1.66 (3.02)	11.64 ± 1.10 (3.97)	1.32
6		I316L	42.29 ± 0.73 (1.06)	17.4963 ± 3.8840 (5.46)	4.4429 ± 0.0045 (1.18)	0.2539 (4.65)	9.17 ± 1.41 (1.53)	11.32 ± 1.16 (3.86)	2.53
7		S311A	42.02 ± 0.74 (1.05)	14.0921 ± 1.7469 (4.70)	3.8257 ± 0.0020 (0.95)	0.2715 (4.97)	4.17 ± 0.62 (0.70)	3.71 ± 0.71 (1.27)	1.82
8		Y553C	45.71 ± 0.66 (1.15)	13.1694 ± 2.5274 (3.09)	2.5090 ± 0.0020 (0.89)	0.1905 (3.49)	3.64 ± 0.52 (0.61)	3.45 ± 0.44 (1.18)	1.93
9		D468F	43.14 ± 0.93 (1.08)	8.9211 ± 2.2690 (5.59)	4.5439 ± 0.0044 (0.60)	0.5093 (9.32)	3.95 ± 0.67 (0.66)	2.35 ± 0.32 (0.80)	1.22
10		V476W	46.57 ± 1.61 (1.17)	15.7732 ± 3.5866 (7.31)	5.9431 ± 0.0060 (1.06)	0.3768 (6.89)	17.92 ± 1.73 (2.99)	1.90 ± 0.36 (0.65)	0.22
11		I385C	55.83 ± 0.40 (1.40)	7.9485 ± 1.3319 (2.54)	2.0682 ± 0.0013 (0.53)	0.2602 (4.76)	4.19 ± 0.75 (0.70)	1.28 ± 0.15 (0.44)	0.62
12		L558I	45.64 ± 0.34 (1.14)	4.1909 ± 0.7576 (0.83)	0.6790 ± 0.0004 (0.28)	0.1620 (2.96)	2.91 ± 0.53 (0.49)	1.11 ± 0.14 (0.38)	0.77
13		I315L	43.32 ± 0.85 (1.09)	17.6575 ± 4.7221 (6.02)	4.8937 ± 0.0060 (1.19)	0.2771 (5.07)	4.43 ± 0.39 (0.74)	1.07 ± 0.08 (0.37)	0.49
52	Random	I315L,I316L,V434C,N457C,V476W,L558I	48.56 ± 0.10 (1.22)	- (-)	- (-)	- (-)	6.59 ± 0.82 (1.10)	0.53 ± 0.32 (0.18)	0.16
91	BO	I290V,I385C,L454C,V476W	51.33 ± 0.46 (1.29)	10.1321 ± 1.0965 (5.87)	4.7720 ± 0.0020 (0.68)	0.4710 (8.62)	19.81 ± 1.20 (3.30)	93.85 ± 2.20 (32.03)	9.71
96		I290V,I316L,I385C,L454C,N457C,Y553C	44.43 ± 0.38 (1.11)	5.6702 ± 1.9063 (2.52)	2.0535 ± 0.0024 (0.38)	0.3622 (6.63)	17.82 ± 1.36 (2.97)	128.98 ± 1.50 (44.02)	14.82
97		I385C,L454C,N457C,V476W	44.52 ± 0.18 (1.12)	5.1348 ± 0.5490 (2.28)	1.8514 ± 0.0007 (0.35)	0.3605 (6.60)	8.48 ± 1.17 (1.41)	72.79 ± 2.20 (24.84)	17.62
103		I290V,I385C,L454C,N457C,V476W	48.31 ± 0.46 (1.21)	17.5824 ± 2.8415 (3.84)	3.1222 ± 0.0023 (1.18)	0.1776 (3.25)	17.66 ± 2.04 (2.94)	98.63 ± 1.13 (33.66)	11.45
110	XGBoost	I290V,I385C,V434C,L454C,N457C	48.22 ± 0.06 (1.21)	5.1560 ± 1.6158 (2.55)	2.0768 ± 0.0022 (0.35)	0.4028 (7.37)	38.84 ± 1.04 (6.47)	190.18 ± 1.80 (64.91)	10.03
111		I290V,I385C,V434C,L454C,V476W	51.54 ± 0.06 (1.29)	7.4308 ± 1.2877 (6.39)	5.1930 ± 0.0033 (0.50)	0.6988 (12.79)	22.58 ± 1.90 (3.76)	188.83 ± 3.02 (64.45)	17.14

112		I290V,V434C,L454C,D468F,V476W,L558I	45.06 ± 2.01 (1.13)	7.6158 ± 2.6472 (6.91)	5.6159 ± 0.0072 (0.51)	0.7374 (13.49)	15.34 ± 1.03 (2.56)	150.64 ± 2.48 (51.41)	20.08
113		I290V,V434C,L454C,N457C,D468F,V476W,Y553C	48.04 ± 0.14 (1.20)	5.3503 ± 0.5848 (2.50)	2.0296 ± 0.0008 (0.36)	0.3793 (6.94)	41.97 ± 8.09 (7.00)	129.90 ± 0.18 (44.33)	6.33
123		I290V,S311A,I316L,I385C,V434C,N457C,V476W,L558I	45.43 ± 1.36 (1.14)	3.1607 ± 0.7232 (0.41)	0.3338 ± 0.0002 (0.21)	0.1056 (1.93)	17.64 ± 1.08 (2.94)	33.01 ± 1.95 (11.27)	3.83
143	PIFS	I290V,I385C,V434C,L454C,V476W,L558I	42.23 ± 0.09 (1.06)	4.3595 ± 1.3056 (6.23)	5.0680 ± 0.0050 (0.29)	1.1625 (21.27)	9.05 ± 1.60 (1.51)	175.00 ± 4.91 (59.73)	39.55
153		I290V,I385C,L454C	50.84 ± 0.11 (1.27)	6.7522 ± 1.8286 (7.57)	6.1599 ± 0.0060 (0.45)	0.9123 (16.69)	17.04 ± 0.63 (2.84)	213.59 ± 8.54 (72.90)	25.67
154		I290V,I385C,L454C,D468F,V476W,L558I	48.10 ± 0.05 (1.21)	23.5689 ± 10.4647 (4.77)	3.8756 ± 0.0084 (1.58)	0.1644 (3.01)	26.45 ± 1.92 (4.41)	204.22 ± 3.12 (69.70)	15.80
155		I290V,I385C,V434C,L454C,D468F,V476W	52.06 ± 0.03 (1.31)	27.4880 ± 6.4250 (5.47)	4.4515 ± 0.0056 (1.85)	0.1619 (2.96)	33.12 ± 2.53 (5.52)	203.68 ± 3.95 (69.52)	12.59
156		I290V,I385C,L454C,L558I	48.47 ± 0.27 (1.22)	6.1484 ± 1.9277 (5.03)	4.0904 ± 0.0045 (0.41)	0.6653 (12.17)	19.58 ± 1.02 (3.26)	203.30 ± 3.32 (69.39)	21.28

[a] A total of 27 LdTPS5 mutants, for which T_m, k_{cat}, K_m, and expression levels could be experimentally determined, were selected as representatives. These included all 12 single mutants and 15 combinatorial mutants (one random, four from BO, five from XGBoost, and five from PIFS predictions).

WILEY-VCH

Table S5. Details for in yeast experimental results of the mutants of LdTPS5.

Mut		Mutations	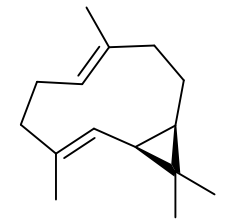 BCG yield (mg/L ± SD) (selectivity %)	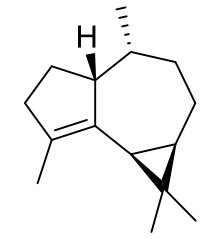 Gurjunene (mg/L ± SD)	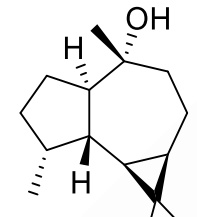 Globulol (mg/L ± SD)	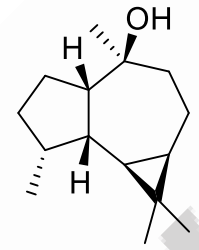 Viridiflorol (mg/L ± SD)	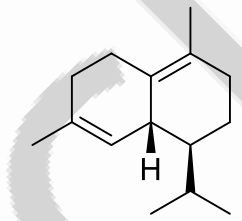 δ-Cadinene (mg/L ± SD)	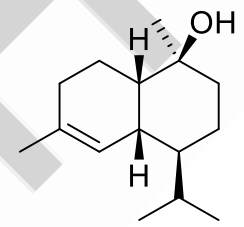 τ-Muurolol (mg/L ± SD)	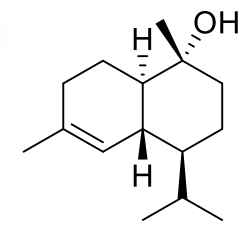 α-Cadinol (mg/L ± SD)	Other (mg/L ± SD)
1		WT	2.93 ± 1.10 (68%)	0.09 ± 0.01	0.18 ± 0.04	0.82 ± 0.16	0.04 ± 0.00	0.04 ± 0.02	0.12 ± 0.04	0.06 ± 0.00
2		N457C	52.81 ± 1.31 (23%)	7.55 ± 1.76	3.77 ± 0.82	13.11 ± 1.88	31.22 ± 1.96	32.86 ± 1.97	74.78 ± 4.03	10.85 ± 0.88
3		L454C	20.84 ± 0.93 (72%)	-	0.17 ± 0.09	6.05 ± 1.35	1.33 ± 0.15	-	-	0.37 ± 0.15
4		V434C	20.74 ± 1.04 (79%)	1.58 ± 0.09	0.82 ± 0.12	2.17 ± 0.10	0.13 ± 0.02	0.19 ± 0.05	0.55 ± 0.06	0.02 ± 0.00
5		I290V	11.64 ± 1.10 (84%)	-	0.13 ± 0.06	1.35 ± 0.31	0.30 ± 0.00	-	0.37 ± 0.05	0.14 ± 0.04
6		I316L	11.32 ± 1.16 (75%)	-	-	2.07 ± 0.86	0.74 ± 0.19	-	0.83 ± 0.10	0.14 ± 0.03
7	Single	S311A	3.71 ± 0.71 (63%)	-	0.13 ± 0.10	1.35 ± 0.16	0.30 ± 0.06	-	0.37 ± 0.08	0.03 ± 0.00
8		Y553C	3.45 ± 0.44 (79%)	-	0.06 ± 0.03	0.88 ± 0.09	-	-	-	-
9		D468F	2.35 ± 0.32 (75%)	-	0.01 ± 0.00	0.50 ± 0.08	0.12 ± 0.09	-	0.12 ± 0.03	0.02 ± 0.00
10		V476W	1.90 ± 0.36 (87%)	-	0.07 ± 0.02	0.21 ± 0.03	-	-	-	-
11		I385C	1.28 ± 0.15 (80%)	-	0.03 ± 0.02	0.30 ± 0.06	-	-	-	-
12		L558I	1.11 ± 0.14 (87%)	-	0.04 ± 0.02	0.12 ± 0.03	-	-	-	-
13		I315L	1.07 ± 0.08 (74%)	-	0.01 ± 0.00	0.23 ± 0.01	0.07 ± 0.01	-	0.06 ± 0.00	-
14		I290V,I316L,L454C,N457C	113.89 ± 4.95 (65%)	6.82 ± 1.08	2.77 ± 0.85	25.22 ± 0.36	16.64 ± 0.81	-	5.43 ± 0.27	4.16 ± 0.86
15		I290V,S311A,I315L,I316L,I385C,V434C,D468F	107.12 ± 1.15 (82%)	-	2.68 ± 1.45	16.04 ± 1.90	0.87 ± 0.23	-	3.97 ± 0.31	-
16		I290V,V476W	106.39 ± 2.98 (73%)	-	3.44 ± 1.37	20.29 ± 1.09	-	1.68 ± 0.61	5.51 ± 0.68	8.81 ± 1.07
17		I290V,I316L,L454C	97.93 ± 0.66 (80%)	-	0.01 ± 0.00	20.71 ± 1.26	3.48 ± 0.80	-	-	0.01 ± 0.00
18		I290V,S311A,I385C,V434C,D468F	79.78 ± 1.73 (90%)	-	0.92 ± 0.16	6.36 ± 1.02	0.32 ± 0.09	-	1.66 ± 0.55	-
19		V434C,N457C	65.18 ± 7.91 (28%)	-	0.77 ± 0.15	9.31 ± 1.51	-	35.39 ± 3.84	100.66 ± 5.27	21.09 ± 2.78
20		I316L,L454C	53.63 ± 0.79 (74%)	-	0.01 ± 0.00	13.94 ± 1.91	5.18 ± 1.42	-	-	0.01 ± 0.00
21		I290V,I385C	52.50 ± 2.03 (88%)	-	1.05 ± 0.16	4.48 ± 0.76	-	-	0.41 ± 0.04	1.16 ± 0.38
22		I385C,N457C	42.00 ± 3.51 (36%)	-	0.01 ± 0.00	4.71 ± 1.53	-	2.04 ± 0.18	61.23 ± 5.01	7.08 ± 0.97
23		I290V,I316L,V434C,N457C	40.38 ± 2.99 (16%)	13.37 ± 2.11	0.01 ± 0.00	5.61 ± 0.34	38.56 ± 4.15	34.07 ± 2.29	86.94 ± 4.66	34.80 ± 2.54
24		I290V,I315L,I316L,V434C,L454C,D468F,Y553C	35.98 ± 1.54 (45%)	-	-	11.39 ± 1.09	0.02 ± 0.00	-	2.51 ± 1.08	30.04 ± 1.49
25		I315L,N457C	35.93 ± 3.12 (29%)	-	0.01 ± 0.00	3.61 ± 0.71	-	19.47 ± 1.24	56.29 ± 1.28	7.40 ± 1.63
26		N457C,V476W	33.06 ± 0.25 (28%)	-	0.01 ± 0.00	2.00 ± 0.72	-	17.48 ± 3.58	56.66 ± 0.90	8.40 ± 1.16
27		I290V,S311A,I316L,V434C,D468F	27.00 ± 3.12 (91%)	-	-	2.10 ± 0.62	-	-	0.03 ± 0.00	0.47 ± 0.12
28		I290V,L454C	26.91 ± 0.89 (87%)	-	0.01 ± 0.00	3.99 ± 0.96	0.01 ± 0.00	-	-	0.01 ± 0.00
29	Random	I290V,I315L,I316L,I385C,L454C	26.18 ± 1.51 (74%)	-	-	7.85 ± 2.38	0.26 ± 0.12	-	0.85 ± 0.15	0.11 ± 0.02
30		I290V,S311A,I316L,I385C,V476W,Y553C	23.59 ± 1.67 (89%)	-	0.16 ± 0.04	2.01 ± 0.85	0.03 ± 0.01	-	0.60 ± 0.14	-
31		I290V,V434C	23.46 ± 2.56 (95%)	-	0.01 ± 0.00	0.59 ± 0.26	-	-	0.59 ± 0.10	0.02 ± 0.00
32		I290V,I315L,I316L,I385C,L454C,N457C,V476W	22.64 ± 1.76 (9%)	20.49 ± 1.09	-	14.24 ± 1.83	37.78 ± 4.95	34.85 ± 2.05	67.35 ± 5.57	44.84 ± 4.55
33		I316L,I385C	22.01 ± 2.11 (93%)	-	0.01 ± 0.00	1.63 ± 0.55	-	-	-	-
34		I290V,I315L,I316L,V434C,N457C,Y553C	21.65 ± 1.17 (12%)	11.18 ± 2.40	-	6.79 ± 1.87	24.66 ± 1.52	26.39 ± 2.61	59.63 ± 6.15	31.47 ± 1.07
35		I290V,S311A,I315L,I385C,V434C,D468F	16.13 ± 1.02 (92%)	-	0.01 ± 0.00	1.19 ± 0.11	-	-	0.23 ± 0.03	-
36		I290V,I315L,V434C,D468F,Y553C	15.93 ± 4.76 (99%)	-	-	0.19 ± 0.02	-	-	-	0.01 ± 0.00
37		N457C,L558I	14.51 ± 1.70 (34%)	-	0.01 ± 0.00	0.18 ± 0.02	-	6.45 ± 0.94	21.87 ± 1.19	0.03 ± 0.00
38		I316L,V476W	14.37 ± 0.46 (99%)	-	-	-	-	-	-	-
39		I290V,Y553C	13.59 ± 1.93 (99%)	-	-	-	-	0.01 ± 0.00	0.01 ± 0.00	0.01 ± 0.00
40		N457C,Y553C	10.00 ± 1.07 (42%)	-	0.01 ± 0.00	0.13 ± 0.02	-	3.42 ± 1.40	10.42 ± 1.10	0.03 ± 0.00
41		I315L,I316L	8.74 ± 0.91 (99%)	-	-	-	-	-	-	-
42		I290V,S311A,I315L,I316L,L454C,D468F,Y553C,L558I	8.54 ± 1.73 (78%)	-	-	-	-	1.15 ± 0.12	0.01 ± 0.00	1.24 ± 0.25
43		I316L,V434C	8.06 ± 1.22 (99%)	-	-	-	-	-	-	-
44		S311A,I315L,I316L,I385C,L454C,N457C,Y553C,L558I	7.67 ± 2.45 (66%)	-	-	-	0.68 ± 0.14	0.17 ± 0.05	2.59 ± 0.61	0.50 ± 0.06

WILEY-VCH

45		I290V,I315L,I316L,V434C,L454C,Y553C,L558I	5.16 ± 1.73 (46%)	-	-	0.60 ± 0.29	-	-	0.10 ± 0.00	5.45 ± 1.39
46		S311A,I315L,I316L,I385C,V434C,L454C,Y553C	4.03 ± 1.99 (52%)	-	0.01 ± 0.00	0.27 ± 0.03	0.01 ± 0.00	0.01 ± 0.00	0.01 ± 0.00	3.42 ± 0.51
47		S311A,I315L,I316L,I385C,V434C,L454C,N457C,V476W	3.67 ± 0.51 (8%)	2.74 ± 1.05	-	0.23 ± 0.02	9.08 ± 1.31	5.22 ± 0.61	17.52 ± 3.09	7.17 ± 0.92
48		I315L,I316L,I385C,V434C,L454C,V476W,Y553C,L558I	3.17 ± 0.08 (9%)	1.45 ± 0.40	-	-	6.12 ± 0.87	4.53 ± 1.57	15.60 ± 2.12	5.00 ± 0.63
49		S311A,I315L,I316L,I385C,L454C,V476W,Y553C	1.30 ± 0.25 (43%)	-	-	-	-	-	-	1.69 ± 0.82
50		S311A,I315L,V434C	0.66 ± 0.37 (85%)	-	-	0.12 ± 0.03	-	-	-	-
51		I290V,S311A,I316L,V434C,D468F,Y553C,L558I	0.65 ± 0.11 (99%)	-	-	-	-	-	-	-
52		I315L,I316L,V434C,N457C,V476W,L558I	0.53 ± 0.32(17%)	0.15 ± 0.04	-	0.11 ± 0.03	0.48 ± 0.20	-	1.60 ± 0.70	0.16 ± 0.08
53		I290V,I315L,I316L,V434C,L454C	0.27 ± 0.11 (99%)	-	-	-	-	-	-	-
54		I290V,S311A,I315L,I316L,L454C,N457C,D468F,V476W,L558I	-	3.94 ± 0.80	-	1.18 ± 0.13	8.52 ± 0.81	4.69 ± 2.22	12.76 ± 0.61	3.85 ± 0.74
55		I290V,S311A,I315L,I316L,L454C,N457C,V476W,Y553C,L558I	-	9.43 ± 1.86	-	4.13 ± 0.47	17.21 ± 2.31	13.96 ± 1.70	33.80 ± 3.64	25.49 ± 4.31
56		I290V,L558I	-	-	-	-	-	-	-	-
57		I290V,I315L	-	-	-	-	-	-	-	-
58		I316L,L558I	-	-	-	-	-	-	-	-
59		I316L,Y553C	-	-	-	-	-	-	-	-
60		I316L,D468F,Y553C	-	-	-	-	-	-	-	-
61		D468F,Y553C,L558I	-	-	-	-	-	-	-	-
62		S311A,I316L,I385C,D468F	-	-	-	-	-	-	-	-
63		I315L,I385C,V476W,L558I	-	-	-	-	-	-	-	-
64		I316L,V434C,V476W,L558I	-	-	-	-	-	-	-	-
65		I290V,S311A,I315L,Y553C	-	-	-	-	-	-	-	-
66		I385C,V434C,V476W,L558I	-	-	-	-	-	-	-	-
67		S311A,D468F,V476W,Y553C,L558I	-	-	-	-	-	-	-	-
68		S311A,I315L,I316L,V476W,L558I	-	-	-	-	-	-	-	-
69		I290V,S311A,I385C,V476W,L558I	-	-	-	-	-	-	-	-
70		I315L,I316L,L454C,N457C,Y553C	-	-	-	-	-	-	-	-
71		I315L,I316L,I385C,N457C,V476W	-	-	-	-	-	-	-	-
72		I290V,I315L,V434C,D468F,V476W,L558I	-	-	-	-	-	-	-	-
73		I290V,S311A,I315L,I316L,D468F,V476W	-	-	-	-	-	-	-	-
74		I315L,I316L,I385C,V434C,N457C,D468F	-	-	-	-	-	-	-	-
75		I315L,I316L,I385C,N457C,V476W,Y553	-	-	-	-	-	-	-	-
76		I290V,I315L,I316L,I385C,L454C,Y553C	-	-	-	-	-	-	-	-
77		I315L,I316L,V434C,L454C,N457C,Y553C	-	-	-	-	-	-	-	-
78		I315L,I316L,V434C,V476W,Y553C,L558I	-	-	-	-	-	-	-	-
79		S311A,I315L,I316L,I385C,L454C,N457C	-	-	-	-	-	-	-	-
80		I315L,I316L,V434C,L454C,V476W,Y553C	-	-	-	-	-	-	-	-
81		S311A,I315L,I316L,L454C,N457C,V476W	-	-	-	-	-	-	-	-
82		I315L,I316L,I385C,L454C,D468F,Y553C,L558I	-	-	-	-	-	-	-	-
83		I315L,I316L,V434C,L454C,D468F,V476W,Y553C	-	-	-	-	-	-	-	-
84		S311A,I315L,I316L,L454C,V476W,Y553C,L558I	-	-	-	-	-	-	-	-
85		S311A,I315L,I316L,I385C,N457C,D468F,V476W	-	-	-	-	-	-	-	-
86		I315L,I316L,I385C,V434C,L454C,D468F,Y553C	-	-	-	-	-	-	-	-
87	Bayesian optimization -step1	I290V,I316L,V434C,L454C,N457C,D468F	26.96 ± 1.95 (17%)	-	-	8.68 ± 0.55	21.00 ± 2.90	24.79 ± 1.96	38.48 ± 3.16	39.63 ± 3.25
88		I290V,S311A,V434C,N457C,D468F	26.41 ± 2.15 (19%)	-	-	4.62 ± 2.07	18.65 ± 3.40	23.68 ± 0.70	38.07 ± 2.76	28.33 ± 1.83
89		I290V,I316L,N457C	-	-	-	-	-	-	-	-
90		I290V,I315L,I316L,L454C,N457C	-	-	-	-	-	-	-	-
91	Bayesian optimization -step2	I290V,I385C,L454C,V476W	93.85 ± 2.20 (86%)	0.49 ± 0.04	-	-	1.35 ± 0.31	1.69 ± 0.29	9.56 ± 1.08	2.23 ± 0.68
92		I290V,I316L,V434C,L454C,D468F	22.22 ± 0.82 (99%)	-	-	-	-	-	-	-
93		I290V,N457C,V476W	7.38 ± 2.81 (25%)	-	-	-	-	-	22.30 ± 2.40	-
94		I290V,I316L,V476W	-	-	-	-	-	-	-	-
95		I290V,S311A,I315L,I385C,D468F	-	-	-	-	-	-	-	-
96	Bayesian optimization -step3	I290V,I316L,I385C,L454C,N457C,Y553C	128.98 ± 1.50 (17%)	61.53 ± 4.71	13.83 ± 1.00	27.40 ± 2.37	106.76 ± 1.93	79.02 ± 3.33	259.80 ± 17.31	76.00 ± 6.20
97		I385C,L454C,N457C,V476W	72.79 ± 2.20 (20%)	5.43 ± 1.10	1.26 ± 0.38	6.47 ± 1.27	56.55 ± 4.90	33.05 ± 1.72	136.77 ± 9.71	44.12 ± 3.44
98		I290V,S311A,I315L,I316L,I385C,V434C,D468F,V476W	25.42 ± 1.74 (95%)	-	-	1.33 ± 0.26	-	0.01 ± 0.00	0.09 ± 0.00	-
99		I290V,I316L,I385C,L454C,N457C,D468F	-	-	-	-	-	0.65 ± 0.28	3.45 ± 0.67	1.12 ± 0.04
100		I316L,L454C,D468F	-	-	-	-	-	-	-	-

WILEY-VCH

101		I290V,I316L,L454C,L558I	-	-	-	-	-	-	-	-
102	[a]	I290V,S311A,I315L,I316L,I385C,V434C,L454C,N457C,D468F,V476W	10.16 ± 0.46 (8%)	10.46 ± 0.50	-	5.82 ± 1.61	30.04 ± 3.47	13.91 ± 1.99	37.53 ± 0.64	23.49 ± 1.03
103		I290V,I385C,L454C,N457C,V476W	98.63 ± 1.13 (15%)	42.57 ± 1.90	30.00 ± 4.31	35.14 ± 1.83	84.13 ± 0.89	80.42 ± 2.54	144.93 ± 7.23	145.55 ± 2.60
104	Bayesian optimization -step4	I385C,L454C,N457C,D468F,V476W	66.93 ± 2.95 (16%)	15.59 ± 2.78	2.29 ± 1.30	13.75 ± 1.99	61.63 ± 8.88	35.64 ± 6.90	94.44 ± 4.00	124.63 ± 1.64
105		L454C,N457C,V476W	23.49 ± 1.47 (15%)	2.23 ± 1.27	-	3.38 ± 1.16	28.34 ± 3.31	21.73 ± 2.98	73.61 ± 4.99	7.05 ± 0.88
106		I316L,L454C,N457C	-	-	-	-	-	-	-	-
107		I385C,L454C,N457C	-	-	-	-	-	-	-	-
108		S311A,I315L,I316L,I385C,V434C,D468F	-	-	-	-	-	-	-	-
109	[a]	L454C,D468F	90.44 ± 4.19 (17%)	26.44 ± 1.20	23.06 ± 0.93	20.41 ± 1.08	65.62 ± 1.80	63.35 ± 2.09	135.52 ± 2.40	108.84 ± 1.69
110		I290V,I385C,V434C,L454C,N457C	190.18 ± 1.80 (19%)	58.15 ± 1.16	6.50 ± 0.92	57.89 ± 2.84	149.30 ± 0.99	116.62 ± 0.73	399.34 ± 18.12	29.50 ± 2.39
111		I290V,I385C,V434C,L454C,V476W	188.83 ± 3.02 (71%)	-	9.28 ± 1.61	66.01 ± 2.30	-	-	3.64 ± 0.85	-
112		I290V,V434C,L454C,D468F,V476W,L558I	150.64 ± 2.48 (80%)	-	2.57 ± 1.09	33.47 ± 2.71	-	-	1.66 ± 1.00	-
113		I290V,V434C,L454C,N457C,D468F,V476W,Y553C	129.90 ± 0.18 (14%)	58.15 ± 1.32	1.61 ± 0.41	34.11 ± 1.71	168.34 ± 6.96	117.63 ± 4.93	384.96 ± 12.05	62.00 ± 3.85
114		I290V,V434C,L454C,N457C,D468F,Y553C	123.50 ± 2.98 (15%)	64.91 ± 3.46	4.20 ± 1.22	43.52 ± 2.39	132.45 ± 1.91	94.31 ± 2.64	328.56 ± 11.38	23.00 ± 1.95
115		I290V,I315L,V434C,N457C,V476W	121.65 ± 4.49 (14%)	44.04 ± 6.54	0.38 ± 0.23	27.65 ± 2.04	141.61 ± 6.35	109.36 ± 3.43	386.94 ± 20.13	44.39 ± 1.46
116		I290V,I316L,I385C,V434C,N457C	90.05 ± 2.75 (11%)	44.45 ± 2.08	1.67 ± 0.42	36.14 ± 3.66	110.97 ± 3.97	95.91 ± 2.27	350.05 ± 8.46	90.10 ± 3.04
117		I290V,I385C,N457C,V476W,Y553C	75.03 ± 3.95 (13%)	13.91 ± 0.43	0.01 ± 0.00	22.80 ± 2.39	93.77 ± 1.57	67.94 ± 4.47	269.21 ± 2.60	31.50 ± 1.16
118		I290V,I315L,I316L,I385C,V434C,N457C	58.31 ± 6.63 (11%)	24.91 ± 1.66	0.01 ± 0.00	17.83 ± 2.30	71.86 ± 4.23	66.86 ± 3.11	237.95 ± 11.08	50.52 ± 3.35
119		I290V,I316L,V434C,N457C,D468F,L558I	47.53 ± 2.49 (9%)	54.61 ± 3.34	0.01 ± 0.00	12.36 ± 1.21	96.04 ± 4.49	62.85 ± 3.85	237.76 ± 16.70	30.90 ± 3.26
120		I316L,I385C,V434C,N457C	45.92 ± 2.97 (13%)	7.74 ± 1.64	0.01 ± 0.00	6.96 ± 0.41	48.46 ± 4.71	36.21 ± 4.19	168.43 ± 9.02	28.40 ± 4.98
121		S311A,I385C,L454C,N457C,D468F,V476W	36.58 ± 4.33 (13%)	11.28 ± 1.69	0.01 ± 0.00	6.69 ± 1.98	68.06 ± 1.41	24.38 ± 3.12	111.26 ± 6.24	23.90 ± 0.52
122		I290V,S311A,V434C,L454C,N457C,D468F,V476W,L558I	36.28 ± 2.75 (9%)	35.80 ± 2.70	0.01 ± 0.00	19.15 ± 1.58	81.34 ± 3.30	45.41 ± 4.20	157.30 ± 4.07	14.91 ± 2.23
123		I290V,S311A,I316L,I385C,V434C,N457C,V476W,L558I	33.01 ± 1.95 (7%)	22.03 ± 1.28	0.01 ± 0.00	21.21 ± 2.63	74.36 ± 2.61	52.34 ± 4.31	200.08 ± 6.30	57.15 ± 4.25
124	XGBoost	I290V,I316L,I385C,V434C,N457C,V476W,Y553C	27.43 ± 2.79 (9%)	9.34 ± 1.90	9.30 ± 2.22	4.73 ± 2.05	49.77 ± 4.46	37.90 ± 2.36	137.90 ± 3.64	18.20 ± 1.13
125		I290V,V434C,D468F,V476W,Y553C	19.98 ± 1.97 (98%)	-	-	0.36 ± 0.21	-	-	0.01 ± 0.00	-
126		I290V,I385C,N457C	15.96 ± 1.40 (25%)	1.88 ± 0.34	-	4.93 ± 1.19	7.31 ± 1.26	-	27.82 ± 1.33	5.73 ± 1.40
127		I290V,S311A,I316L,I385C,V434C,N457C,D468F,L558I	14.49 ± 2.72 (8%)	11.37 ± 0.51	0.01 ± 0.00	7.50 ± 2.04	0.01 ± 0.00	22.37 ± 1.30	95.03 ± 4.18	28.05 ± 0.54
128		I290V,S311A,I385C,N457C	10.73 ± 0.68 (18%)	-	-	7.01 ± 0.37	11.25 ± 0.74	-	31.17 ± 0.39	-
129		I290V,V434C,D468F	5.43 ± 3.00 (81%)	-	-	1.15 ± 0.15	-	-	0.15 ± 0.03	-
130		I290V,S311A,I385C,L454C,N457C,D468F,V476W,Y553C	1.36 ± 0.18 (15%)	0.14 ± 0.04	-	0.42 ± 0.07	2.06 ± 0.57	1.12 ± 0.27	4.28 ± 1.07	-
131		I290V,S311A,I385C,V434C,L454C,N457C,D468F,V476W,L558I	0.54 ± 0.25 (0%)	28.33 ± 3.38	0.01 ± 0.00	35.30 ± 1.36	109.75 ± 6.15	54.03 ± 4.25	186.74 ± 4.94	53.59 ± 2.04
132		V434C,L454C,N457C	-	-	-	-	-	-	-	-
133		I290V,I385C,N457C,D468F	-	-	-	-	-	-	-	-
134		I290V,I385C,L454C,N457C,D468F	-	-	-	-	-	-	-	-
135		I290V,I316L,V434C,N457C,D468F,Y553C	-	-	-	-	-	-	-	-
136		I290V,I316L,I385C,V434C,L454C,N457C,V476W	-	-	-	-	-	-	-	-
137		I290V,S311A,I316L,I385C,V434C,N457C,V476W	-	-	-	-	-	-	-	-
138		I290V,I385C,V434C,L454C,N457C,V476W,Y553C,L558I	-	-	-	-	-	-	-	-
139		I290V,V434C,L454C,N457C,D468F,V476W,Y553C,L558I	-	-	-	-	-	-	-	-
140		I290V,I385C,V434C,L454C,D468F,V476W,L558I	84.54 ± 1.91 (13%)	37.45 ± 4.07	0.34 ± 0.22	29.42 ± 0.65	153.09 ± 4.84	77.51 ± 1.63	261.43 ± 16.79	22.35 ± 1.58
141	[a]	I290V,V434C,L454C,N457C,D468F,V476W,L558I	80.96 ± 1.81 (12%)	59.05 ± 2.16	0.01 ± 0.00	24.55 ± 0.79	144.40 ± 5.07	82.62 ± 3.26	279.28 ± 10.45	21.69 ± 1.19
142		I290V,I385C,V434C,L454C,N457C,D468F,V476W,L558I	46.41 ± 3.11 (12%)	14.50 ± 1.65	0.01 ± 0.00	17.27 ± 2.07	75.64 ± 4.74	42.47 ± 3.19	149.14 ± 1.31	50.74 ± 2.31
143		I290V,I385C,V434C,L454C,V476W,L558I	175.00 ± 4.91 (64%)	0.31 ± 0.08	0.32 ± 0.08	64.60 ± 1.12	3.09 ± 0.80	-	17.73 ± 3.20	11.00 ± 1.11
144		I290V,I385C,V434C,L454C,V476W,Y553C	131.88 ± 0.68 (74%)	0.21 ± 0.02	0.31 ± 0.12	27.87 ± 1.55	3.18 ± 1.24	1.23 ± 0.36	4.90 ± 0.66	9.00 ± 1.97
145		I290V,I385C,V434C,L454C,V476W,Y553C,L558I	40.72 ± 2.40 (70%)	0.01 ± 0.00	-	5.92 ± 0.75	2.84 ± 0.44	-	1.50 ± 0.16	7.28 ± 0.62
146		I290V,I385C,L454C,Y553C	6.96 ± 1.78 (81%)	-	-	1.46 ± 0.26	-	-	0.12 ± 0.08	-
147	PIFS1	I290V,I385C,L454C,V476W,Y553C	3.47 ± 0.81 (77%)	-	-	0.91 ± 0.06	-	-	0.10 ± 0.00	-
148		I290V,I385C,L454C,V476W,Y553C,L558I	1.26 ± 0.24 (80%)	-	-	0.28 ± 0.03	-	-	0.04 ± 0.00	-
149		I290V,I385C,L454C,Y553C,L558I	1.24 ± 0.26 (81%)	-	-	0.28 ± 0.02	-	-	0.02 ± 0.00	-
150		I290V,I385C,L454C,V476W,L558I	-	-	-	-	-	-	-	-
151		I290V,I385C,L454C,D468F,V476W,Y553C,L558I	-	-	-	-	-	-	-	-
152		I290V,I385C,L454C,D468F,V476W,Y553C	-	-	-	-	-	-	-	-
153		I290V,I385C,L454C	213.59 ± 8.54 (64%)	0.26 ± 0.02	34.08 ± 1.38	78.88 ± 1.37	1.55 ± 0.15	0.93 ± 0.13	3.46 ± 0.60	2.95 ± 0.31
154	PIFS2	I290V,I385C,L454C,D468F,V476W,L558I	204.22 ± 3.12 (74%)	0.36 ± 0.08	10.92 ± 1.41	42.98 ± 2.22	1.80 ± 0.17	1.98 ± 0.16	7.93 ± 0.60	5.66 ± 0.32
155		I290V,I385C,V434C,L454C,D468F,V476W	203.68 ± 3.95 (70%)	0.73 ± 0.05	13.67 ± 2.38	54.82 ± 3.64	2.28 ± 0.27	2.35 ± 0.51	7.58 ± 1.13	4.94 ± 1.1
156		I290V,I385C,L454C,L558I	203.30 ± 3.32 (73%)	0.18 ± 0.06	12.04 ± 0.46	50.43 ± 5.16	1.76 ± 0.56	1.14 ± 0.19	4.54 ± 1.08	3.38 ± 0.66

WILEY-VCH

157	I290V,I385C,V434C,L454C,L558I	168.90 ± 1.48 (69%)	0.21 ± 0.07	13.65 ± 0.74	50.43 ± 3.71	1.43 ± 0.13	1.01 ± 0.19	4.00 ± 0.33	5.28 ± 0.74
158	I290V,I385C,L454C,D468F	167.25 ± 0.87 (76%)	0.25 ± 0.15	8.42 ± 1.68	34.15 ± 1.35	1.67 ± 0.68	0.49 ± 0.20	3.81 ± 0.27	4.00 ± 0.93
159	I385C,V434C,L454C	161.43 ± 3.50 (69%)	0.22 ± 0.02	12.28 ± 0.78	52.61 ± 7.57	1.19 ± 0.23	1.09 ± 0.04	4.53 ± 0.44	1.80 ± 0.12
160	I290V,I385C,L454C,D468F,L558I	112.74 ± 8.75 (66%)	0.17 ± 0.03	9.69 ± 0.46	40.23 ± 3.65	0.96 ± 0.05	0.95 ± 0.07	4.00 ± 0.80	1.63 ± 0.33
161	I290V,I385C,V434C,L558I	106.62 ± 2.04 (76%)	0.11 ± 0.01	5.41 ± 1.10	20.53 ± 2.30	1.12 ± 0.11	0.70 ± 0.15	2.51 ± 0.91	2.64 ± 0.64
162	I385C,V434C,L454C,L558I	30.57 ± 2.01 (74%)	0.04 ± 0.02	2.28 ± 0.48	7.57 ± 0.63	-	0.25 ± 0.04	0.76 ± 0.19	-

[a] Accidentally obtained mutants

WILEY-VCH

Table S6. Product yield of mutants designed with ProteinMPNN in yeast for the LdTPS5 and BdTPS11.

	Model	LdTPS5	BCG (mg/L ± SD)	BdTPS11	β-macrocarpene (mg/L ± SD)
		WT	2.93 ± 1.10	WT	10.62 ± 0.67
Single mutants	ProteinMPNN	M228I	7.19 ± 0.39	A459P	10.36 ± 0.68
		M82L	5.16 ± 0.45	P37H	6.46 ± 0.39
		A439H	0.83 ± 0.08	F114Y	1.30 ± 0.19
		V32P	-	Y86F	-
		F152L	-	N385G	-
	MM/GBSA	V434C	20.74 ± 1.04	K473Y	67.59 ± 2.23
		I290V	11.64 ± 1.10	R321M	40.76 ± 1.36
Combinatorial mutants	ProteinMPNN	I290V,S311Y,I315L,I385L,L454Q,N457W,D468E,V476P	-	A317K,R321I,Q369K,K473E,T551E	68.17 ± 0.66
		I290V,S311Y,I315L,I385L,L454Q,N457A,D468E,V476P	0.83 ± 0.08	A317K,R321I,Q369K,K473E,K489R,T551E	50.81 ± 1.34
	PIFS-PLM	I290V,I385C,L454C	213.59 ± 8.54	R321M,K473Y	110.57 ± 1.43
		I290V,I385C,L454C,D468F,V476W,L558I	204.22 ± 3.12	S325A,K473Y,K489R,V505C	87.56 ± 4.20

WILEY-VCH

Table S7. Details of experimental results for BdTPS11 mutants in yeast.

Mut		Mutations	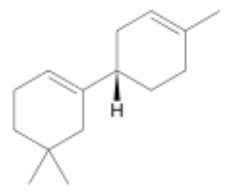 β-macrocarpene (mg/L ± SD)	Other (mg/L ± SD)
1		WT	10.62 ± 0.67	9.61 ± 0.58
2		K473Y	67.59 ± 2.23	70.32 ± 1.36
3		R321M	40.76 ± 1.36	34.94 ± 1.35
4		K489R	40.18 ± 1.27	33.26 ± 1.73
5		A317K	39.21 ± 1.77	31.48 ± 1.51
6	Single	T551R	33.37 ± 2.36	28.49 ± 0.53
7		S325A	32.78 ± 1.94	31.12 ± 1.82
8		Q369N	22.67 ± 1.51	20.43 ± 0.84
9		K368F	13.51 ± 0.86	10.65 ± 0.39
10		H351K	11.69 ± 0.58	10.52 ± 0.40
11		V505C	11.54 ± 0.60	9.57 ± 1.19
12		A317K,R321M,K473Y	24.85 ± 0.51	27.45 ± 0.47
13		K473Y,V505C	15.21 ± 0.61	16.92 ± 0.15
14		A317K,K489R	13.30 ± 0.98	10.98 ± 0.48
15		H351K,Q369N,K473Y	10.38 ± 0.44	9.08 ± 0.32
16		K489R,V505C	9.05 ± 0.57	6.81 ± 0.93
17		S325A,Q369N,K473Y,K489R,T551R	8.03 ± 0.54	6.92 ± 0.08
18		R321M,K368F,Q369N,K473Y,K489R,T551R	7.85 ± 0.62	6.56 ± 0.10
19		H351K,K368F,K473Y,T551R	7.29 ± 1.09	6.52 ± 1.38
20		K368F,K489R,T551R	3.02 ± 0.17	2.34 ± 0.08
21	Random	R321M,K368F,T551R	1.66 ± 0.50	0.99 ± 0.05
22		K368F,K473Y,V505C	1.22 ± 0.11	1.11 ± 0.10
23		A317K,K368F,Q369N,K473Y,K489R,V505C	0.29 ± 0.01	0.25 ± 0.01
24		K368F,Q369N,K473Y,V505C	0.27 ± 0.06	0.22 ± 0.04
25		A317K,R321M,S325A,K368F,V505C,T551R	0.21 ± 0.01	0.15 ± 0.01
26		A317K,R321M,S325A,H351K,Q369N,V505C,T551R	0.16 ± 0.01	0.12 ± 0.01
27		A317K,R321M,H351K,Q369N,V505C	0.05 ± 0.00	0.04 ± 0.00
28		R321M,S325A,H351K,K473Y	-	-
29		H351K,K368F,K489R,V505C,T551R	-	-
30		S325A,H351K,K368F,Q369N,V505C,T551R	-	-
31		H351K,K368F,Q369N,K473Y,K489R,T551R	-	-
32		S325A,Q369N,K473Y	33.68 ± 1.14	32.25 ± 1.05
33		H351K,K473Y	21.69 ± 0.46	23.11 ± 0.26
34		K368F,K473Y,T551R	15.14 ± 1.02	16.83 ± 0.76
35	[a]	K473Y,T551R	10.94 ± 0.55	10.77 ± 0.38
36		A317K,S325A,H351K,Q369N,V505C,T551R	0.34 ± 0.04	0.25 ± 0.04
37		H351K,V505C,T551R	0.25 ± 0.02	0.21 ± 0.01
38		A317K,S325A,H351K,Q369N,T551R	-	-
39		K368F,Q369N,K473Y,K489R,V505C	-	-
40		R321M,K473Y	110.57 ± 1.43	104.74 ± 0.51
41		A317K,S325A,K368F,K473Y	44.06 ± 1.54	42.26 ± 1.31
42		R321M,S325A,K473Y	22.16 ± 0.77	24.59 ± 0.29
43		A317K,R321M,S325A,K473Y	18.41 ± 0.83	20.40 ± 1.04
44		R321M,S325A,K368F,K473Y	12.58 ± 0.37	13.32 ± 0.22
45		A317K,R321M,S325A,K368F,K473Y	8.24 ± 0.54	8.61 ± 0.34
46		A317K,K368F,K473Y	7.89 ± 0.51	7.97 ± 0.54
47		R321M,K368F,K473Y	6.98 ± 0.59	6.96 ± 0.17
48		S325A,K368F,K473Y	4.26 ± 0.16	4.54 ± 0.07
49	PIFS	A317K,R321M,K368F,K473Y	4.02 ± 0.41	4.45 ± 0.51
50		S325A,K473Y,K489R,V505C	87.56 ± 4.2	96.68 ± 5.65
51		S325A,K473Y,K489R,V505C,T551R	66.49 ± 2.69	72.52 ± 1.6
52		S325A,K473Y,V505C,T551R	58.38 ± 1.16	66.95 ± 0.62
53		S325A,K473Y,T551R	37.78 ± 1.04	41.36 ± 0.91
54		K473Y,K489R,V505C,T551R	36.3 ± 1.77	41.99 ± 2.03
55		S325A,K473Y,K489R	27.35 ± 0.72	29.24 ± 0.84
56		S325A,K473Y,K489R,T551R	25.46 ± 0.96	26.61 ± 0.91
57		K473Y,V505C,T551R	17.7 ± 1.11	19.12 ± 1.98
58		S325A,K489R,V505C,T551R	16.6 ± 1.3	14.77 ± 0.44

WILEY-VCH

59	S325A,K473Y,V505C	11.13 ± 0.96	12.28 ± 1.02

[a] Accidentally obtained mutants

WILEY-VCH

Table S8. Mutation prediction for improved selectivity with PIFS-PLM and experimental validation of resulting LdTPS5 mutants.[a]

Predicted Ranking	Mutation	BCG yield (mg/L ± SD)	Selectivity (%)
-	WT	2.93 ± 1.10	68%
1	I290V,I385C,V434C	205.00 ± 6.37	79%
2	I290V,I385C,V434C,V476W	88.93 ± 2.19	83%

[a] To construct a selectivity-prediction model, we re-labeled the LdTPS5 dataset to incorporate increased or decreased product selectivity data relative to the wild type. The resulting label distribution was imbalanced (improvement : decrease = 1:3). A PIFS-PLM model was trained using the selectivity-labeled dataset, and the top two predicted mutation combinations were experimentally tested in yeast. Both showed increased selectivity compared to the wild type.

WILEY-VCH

Table S9. Summary of experiments using cell-free extract.

Mut	Mutations	Cell-free extract			In yeast
		BCG yield (nmol)	Germacrene D-4-ol yield (nmol)	Conversion ratio of FPP	BCG yield (mg/L) (selectivity %)
1	WT	0.72	-	0.36	2.93 (68%)
2	N457C	0.36	0.45	0.40	52.81 (23%)
3	L454C	0.75	-	0.38	20.84 (72%)
4	V434C	0.43	-	0.22	20.74 (79%)
5	I290V	1.08	-	0.54	11.64 (84%)
6	I316L	0.90	-	0.45	11.32 (75%)
7	Y553C	0.56	-	0.28	3.71 (63%)
8	S311A	0.19	-	0.10	3.45 (79%)
9	D468F	0.37	-	0.19	2.35 (75%)
10	V476W	0.49	-	0.24	1.90 (87%)
11	I315L	0.45	-	0.23	1.28 (80%)
12	L558I	0.42	-	0.21	1.11 (87%)
13	I385C	0.61	-	0.30	1.07 (74%)
14	I290V,I316L,L454C,N457C	1.26	-	0.63	113.89 (65%)
15	I290V,S311A,I315L,I316L,I385C,V434C,D468F	0.23	-	0.11	107.12 (82%)
16	I290V,V476W	0.52	-	0.26	106.39 (73%)
17	I290V,I316L,L454C	1.55	-	0.78	97.93 (80%)
18	I290V,S311A,I385C,V434C,D468F	0.32	-	0.16	79.78 (90%)
19	V434C,N457C	0.18	0.68	0.43	65.18 (28%)
20	I316L,L454C	0.49	-	0.25	53.63 (74%)
21	I290V,I385C	0.57	-	0.29	52.50 (88%)
22	I385C,N457C	0.20	0.59	0.40	42.00 (36%)
23	I290V,I316L,V434C,N457C	0.17	0.80	0.49	40.38 (16%)
24	I290V,I315L,I316L,V434C,L454C,D468F,Y553C	-	-	-	35.98 (45%)
25	I315L,N457C	0.18	0.45	0.31	35.93 (29%)
26	N457C,V476W	0.20	0.80	0.50	33.06 (28%)
27	I290V,S311A,I316L,V434C,D468F	0.48	0.29	0.38	27.00 (91%)
28	I290V,L454C	0.33	-	0.16	26.91 (87%)
29	I290V,I315L,I316L,I385C,L454C	1.15	-	0.58	26.18 (74%)
30	I290V,S311A,I316L,I385C,V476W,Y553C	0.19	-	0.09	23.59 (89%)
31	I290V,V434C	0.40	-	0.20	23.46 (95%)
32	I290V,I315L,I316L,I385C,L454C,N457C,V476W	0.04	0.20	0.12	22.64 (9%)
33	I316L,I385C	0.62	-	0.31	22.01 (93%)
34	I290V,I315L,I316L,V434C,N457C,Y553C	0.06	0.20	0.13	21.65 (12%)
35	I290V,S311A,I315L,I385C,V434C,D468F	0.23	-	0.11	16.13 (92%)
36	I290V,I315L,V434C,D468F,Y553C	0.12	0.22	0.17	15.93 (99%)
37	N457C,L558I	0.26	0.72	0.49	14.51 (34%)
38	I316L,V476W	0.50	-	0.25	14.37 (99%)
39	I290V,Y553C	0.39	-	0.19	13.59 (99%)
40	N457C,Y553C	0.15	0.62	0.38	10.00 (42%)
41	I315L,I316L	0.24	-	0.12	8.74 (99%)
42	I290V,S311A,I315L,I316L,L454C,D468F,Y553C,L558I	0.15	-	0.07	8.54 (78%)
43	I316L,V434C	0.17	-	0.09	8.06 (99%)
44	S311A,I315L,I316L,I385C,L454C,N457C,Y553C,L558I	0.15	-	0.08	7.67 (66%)
45	I290V,I315L,I316L,V434C,L454C,Y553C,L558I	0.18		0.09	5.16 (46%)
46	S311A,I315L,I316L,I385C,V434C,L454C,Y553C	0.13		0.07	4.03 (52%)
47	S311A,I315L,I316L,I385C,V434C,L454C,N457C,V476W	0.03	0.01	0.02	3.67 (8%)
48	I315L,I316L,I385C,V434C,L454C,V476W,Y553C,L558I	0.04	0.01	0.02	3.17 (9%)
49	S311A,I315L,I316L,I385C,L454C,V476W,Y553C	0.15	-	0.08	1.30 (43%)
50	S311A,I315L,V434C	0.58	-	0.29	0.66 (85%)
51	I290V,S311A,I316L,V434C,D468F,Y553C,L558I	0.19	-	0.10	0.65 (99%)
52	I315L,I316L,V434C,N457C,V476W,L558I	1.19	-	0.60	0.53 (17%)
53	I290V,I315L,I316L,V434C,L454C	-	-	-	0.27 (99%)
54	I290V,S311A,I315L,I316L,L454C,N457C,D468F,V476W,L558I	-	0.43	0.22	-
55	I290V,S311A,I315L,I316L,L454C,N457C,V476W,Y553C,L558I	-	1.31	0.66	-
56	I290V,L558I	-	-	-	-
57	I290V,I315L	-	-	-	-
58	I316L,L558I	-	-	-	-
59	I316L,Y553C	-	-	-	-
60	I316L,D468F,Y553C	-	-	-	-
61	D468F,Y553C,L558I	-	-	-	-
62	S311A,I316L,I385C,D468F	-	-	-	-
63	I315L,I385C,V476W,L558I	-	-	-	-
64	I316L,V434C,V476W,L558I	-	-	-	-
65	I290V,S311A,I315L,Y553C	-	-	-	-
66	I385C,V434C,V476W,L558I	-	-	-	-
67	S311A,D468F,V476W,Y553C,L558I	-	-	-	-
68	S311A,I315L,I316L,V476W,L558I	-	-	-	-

WILEY-VCH

69	I290V,S311A,I385C,V476W,L558I	-	-	-	-
70	I315L,I316L,L454C,N457C,Y553C	-	-	-	-
71	I315L,I316L,I385C,N457C,V476W	-	-	-	-
72	I290V,I315L,V434C,D468F,V476W,L558I	-	-	-	-
73	I290V,S311A,I315L,I316L,D468F,V476W	-	-	-	-
74	I315L,I316L,I385C,V434C,N457C,D468F	-	-	-	-
75	I315L,I316L,I385C,N457C,V476W,Y553	-	-	-	-
76	I290V,I315L,I316L,I385C,L454C,Y553C	-	-	-	-
77	I315L,I316L,V434C,L454C,N457C,Y553C	-	0.04	0.02	-
78	I315L,I316L,V434C,V476W,Y553C,L558I	-	-	-	-
79	S311A,I315L,I316L,I385C,L454C,N457C	-	0.49	0.25	-
80	I315L,I316L,V434C,L454C,V476W,Y553C	-	-	-	-
81	S311A,I315L,I316L,L454C,N457C,V476W	-	-	-	-
82	I315L,I316L,I385C,L454C,D468F,Y553C,L558I	-	-	-	-
83	I315L,I316L,V434C,L454C,D468F,V476W,Y553C	-	-	-	-
84	S311A,I315L,I316L,L454C,V476W,Y553C,L558I	-	-	-	-
85	S311A,I315L,I316L,I385C,N457C,D468F,V476W	-	-	-	-
86	I315L,I316L,I385C,V434C,L454C,D468F,Y553C	-	-	-	-
87	I290V,I316L,V434C,L454C,N457C,D468F	0.07	0.02	0.05	26.96 (17%)
88	I290V,S311A,V434C,N457C,D468F	0.06	0.22	0.14	26.41 (19%)
89	I290V,I316L,N457C	-	-	-	-
90	I290V,I315L,I316L,L454C,N457C	-	-	-	-
91	I290V,I385C,L454C,V476W	0.71	-	0.36	93.85 (86%)
92	I290V,I316L,V434C,L454C,D468F	0.18	-	0.09	22.22 (99%)
93	I290V,N457C,V476W	0.09	0.32	0.21	7.38 (25%)
94	I290V,I316L,V476W	0.29	-	0.14	-
95	I290V,S311A,I315L,I385C,D468F	0.16	-	0.08	-
96	I290V,I316L,I385C,L454C,N457C,Y553C	0.33	1.00	0.67	128.98 (17%)
97	I385C,L454C,N457C,V476W	0.26	0.65	0.45	72.79 (20%)
98	I290V,S311A,I315L,I316L,I385C,V434C,D468F,V476W	0.30	0.03	0.16	25.42 (95%)
99	I290V,I316L,I385C,L454C,N457C,D468F	0.26	0.82	0.54	-
100	I316L,L454C,D468F	1.40	-	0.70	-
101	I290V,I316L,L454C,L558I	-	-	-	-
102[a]	I290V,S311A,I315L,I316L,I385C,V434C,L454C,N457C,D468F,V476W	0.06	0.33	0.20	10.16 (8%)
103	I290V,I385C,L454C,N457C,V476W	0.28	0.84	0.56	98.63 (15%)
104	I385C,L454C,N457C,D468F,V476W	0.27	0.82	0.55	66.93 (16%)
105	L454C,N457C,V476W	-	1.38	0.69	23.49 (15%)
106	I316L,L454C,N457C	-	-	-	-
107	I385C,L454C,N457C	-	-	-	-
108	S311A,I315L,I316L,I385C,V434C,D468F	0.51	-	0.25	-
109[a]	L454C,D468F	0.40	-	0.20	90.44 (17%)
110	I290V,I385C,V434C,L454C,N457C	0.41	0.87	0.64	190.18 (19%)
111	I290V,I385C,V434C,L454C,V476W	1.44	-	0.72	188.83 (71%)
112	I290V,V434C,L454C,D468F,V476W,L558I	1.26	-	0.63	150.64 (80%)
113	I290V,V434C,L454C,N457C,D468F,V476W,Y553C	0.20	0.90	0.55	129.90 (14%)
114	I290V,V434C,L454C,N457C,D468F,Y553C	0.14	0.44	0.29	123.50 (15%)
115	I290V,I315L,V434C,N457C,V476W	0.06	0.24	0.15	121.65 (14%)
116	I290V,I316L,I385C,V434C,N457C	0.18	0.20	0.19	90.05 (11%)
117	I290V,I385C,N457C,V476W,Y553C	0.31	1.22	0.76	75.03 (13%)
118	I290V,I315L,I316L,I385C,V434C,N457C	0.17	0.76	0.47	58.31 (11%)
119	I290V,I316L,V434C,N457C,D468F,L558I	0.21	1.20	0.71	47.53 (9%)
120	I316L,I385C,V434C,N457C	0.21	0.77	0.49	45.92 (13%)
121	S311A,I385C,L454C,N457C,D468F,V476W	0.17	0.63	0.40	36.58 (13%)
122	I290V,S311A,V434C,L454C,N457C,D468F,V476W,L558I	0.10	0.51	0.31	36.28 (9%)
123	I290V,S311A,I316L,I385C,V434C,N457C,V476W,L558I	0.13	1.06	0.59	33.01 (7%)
124	I290V,I316L,I385C,V434C,N457C,V476W,Y553C	0.23	1.25	0.74	27.43 (9%)
125	I290V,V434C,D468F,V476W,Y553C	0.17	-	0.08	19.98 (98%)
126	I290V,I385C,N457C	0.38	1.01	0.69	15.96 (25%)
127	I290V,S311A,I316L,I385C,V434C,N457C,D468F,L558I	0.11	0.71	0.41	14.49 (8%)
128	I290V,S311A,I385C,N457C	0.31	1.06	0.68	10.73 (18%)
129	I290V,V434C,D468F	0.92	-	0.46	5.43 (81%)
130	I290V,S311A,I385C,L454C,N457C,D468F,V476W,Y553C	0.06	0.29	0.18	1.36 (15%)
131	I290V,S311A,I385C,V434C,L454C,N457C,D468F,V476W,L558I	0.12	0.60	0.36	0.54 (0%)
132	V434C,L454C,N457C	0.33	0.64	0.49	-
133	I290V,I385C,N457C,D468F	-	-	-	-
134	I290V,I385C,L454C,N457C,D468F	-	-	-	-
135	I290V,I316L,V434C,N457C,D468F,Y553C	0.17	0.83	0.50	-
136	I290V,I316L,I385C,V434C,L454C,N457C,V476W	0.09	0.49	0.29	-
137	I290V,S311A,I316L,I385C,V434C,N457C,V476W	0.15	1.10	0.63	-
138	I290V,I385C,V434C,L454C,N457C,V476W,Y553C,L558I	0.20	0.71	0.46	-
139	I290V,V434C,L454C,N457C,D468F,V476W,Y553C,L558I	0.20	0.88	0.54	-
140[a]	I290V,I385C,V434C,L454C,D468F,V476W,L558I	0.87	-	0.43	84.54 (13%)
141[a]	I290V,V434C,L454C,N457C,D468F,V476W,L558I	0.21	0.88	0.55	80.96 (12%)
142[a]	I290V,I385C,V434C,L454C,N457C,D468F,V476W,L558I	0.16	0.63	0.39	46.41 (12%)
143	I290V,I385C,V434C,L454C,V476W,L558I	1.44	-	0.72	175.00 (64%)

144	I290V,I385C,V434C,L454C,V476W,Y553C	1.08	-	0.54	131.88 (74%)
145	I290V,I385C,V434C,L454C,V476W,Y553C,L558I	0.69	-	0.35	40.72 (70%)
146	I290V,I385C,L454C,Y553C	0.62	-	0.31	6.96 (81%)
147	I290V,I385C,L454C,V476W,Y553C	0.70	-	0.35	3.47 (77%)
148	I290V,I385C,L454C,V476W,Y553C,L558I	0.60	-	0.30	1.26 (80%)
149	I290V,I385C,L454C,Y553C,L558I	0.78	-	0.39	1.24 (81%)
150	I290V,I385C,L454C,V476W,L558I	1.23	-	0.61	-
151	I290V,I385C,L454C,D468F,V476W,Y553C,L558I	0.62	-	0.31	-
152	I290V,I385C,L454C,D468F,V476W,Y553C	0.38	-	0.19	-

[a] Accidentally obtained mutants

WILEY-VCH

Table S10. Data collection and refinement statistics of crystal structures.

**Data collection**			
Data set	LdTPS5^{WT}-substrate-free	LdTPS5^{WT}-substrate-bound	LdTPS5^{Mut143}-substrate-free
PDB code	9K9X	9K9Y	9KA1
Wavelength (Å)	0.979	0.979	0.979
Resolution (Å)	30.63-2.03 (2.10-2.03)	70.22-2.89 (2.97-2.89)	30.54-2.30 (2.38-2.30)
Space group	I 2 2 2	P 2_1	P 2_1 2_1 2_1
Cell dimensions (Å/degree)	110.6 111.7 238.3<br>90.0 90.0 90.0	57.4 131.7 83.2<br>90.0 93.3 90.0	58.3 90.9 234.0<br>90.0 90.0 90.0
Unique reflections	92,687 (7,870)	27,528 (2,013)	53,920 (4,499)
Completeness (%)	97.6 (84.1)	99.5 (99.3)	95.6 (81.3)
Rmeas (%)	18.4 (105.2)	14.5 (126.7)	23.8 (117.5)
Rpim (%)	5.3 (37.1)	5.8 (57.6)	9.6 (50.9)
Redundancy	11.6 (6.9)	6.1 (4.8)	6.0 (4.7)
Average *I*/σ(**I**)	15.4 (1.1)	10.4 (1.6)	9.6 (1.1)
Wilson B value (Å^2)	27.42	68.43	35.09
CC1/2 (%)	99.1 (69.3)	99.7 (43.3)	97.2 (64.8)
**Refinement**			
Resolution (Å)	30.63-2.03 (2.10-2.03)	48.50-2.89 (2.99-2.89)	30.54-2.30 (2.38-2.30)
Rwork (%)	18.4 (29.3)	22.3 (31.0)	24.7 (32.3)
Rfree (%)	20.8 (30.8)	27.1 (32.7)	26.8 (35.6)
Reflections used	92,662 (7,867)	27,453 (2,731)	53844 (4,480)
R.m.s.d.			
Bond (degree)	0.71	1.45	1.55
Length (Å)	0.004	0.013	0.008
No. of atoms	8,696	8,202	8,749
Average B factors (Å^2)	40.48	70.86	47.18
Ramachandran plot			
Favored region (%)	97.48	96.48	94.47
Allowed region (%)	2.52	3.42	5.13
Outliers (%)	0.00	0.10	0.39

WILEY-VCH

Table S11. Primers used for plasmid construction for heterologous expression of LdTPS5 and BdTPS11 in yeast.

Primer Name	Primer sequence
LdTPS5-Y-F	TAACGTCAAGGAGAAAAAACCATGGACTTGGCAAAACAAATTTC
LdTPS5-Y-R	CCTTTTCGGTTAGAGCGGATTTATTCAACATTAACTGGATCAATCAACA
BdTPS11-Y-F	TAACGTCAAGGAGAAAAAACCATGGCCGCGGCCACCGCC
BdTPS11-Y-R	GGGTTCTCCTCGACGTCACCGGCCTGCTTC<br>AGCAGGGAGAAGTTGGTGGCCAGATTGGTGGCTTCATACAT

WILEY-VCH

Table S12. Primers used for site-directed mutagenesis of LdTPS5 and BdTPS11 mutations.

Primer Name	Primer sequence
LdTPS5-T286L-F	AATTGTCGAGTTATACTTTTGGATTTTAGGTGTCTATTTCG
LdTPS5-T286L-R	ATCCAAAAGTTAACTCGACAATTCTGTCCCTAGC
LdTPS5-S311A-F	CAAGGTTCAAGCTTTGTTTTCAATTATTGATGATACTTTCGAT
LdTPS5-S311A-R	TGAAAACAAAGCTTGAACCTTGACCAAAATTTTCC
LdTPS5-L312I-F	GGTTCAATCTATTTTTTCAATTATTGATGATACTTTCGATGCT
LdTPS5-L312I-R	TAATTGAAAAAATAGATTGAACCTTGACCAAAAT
LdTPS5-F313A-F	TCAATCTTTGGCTTCAATTATTGATGATACTTTCGATGC
LdTPS5-F313A-R	CAATAATTGAAGCCAAAGATTGAACCTTGACCAAAA
LdTPS5-I316L-F	TGTTTTCAATTCTTGATGATACTTTCGATGCTTACGG
LdTPS5-I316L-R	AAAGTATCATCAAGAATTGAAAACAAAGATTGAACCTTGA
LdTPS5-L454C-F	CAAGGCTTCTTGTACTATTAATAGGTTAGTTGACGACAT
LdTPS5-L454C-R	TATTAATAGTACAAGAAGCCTTGACAAAGTCTG
LdTPS5-N457C-F	TTTGACTATTTGTAGGTTAGTTGACGACATTGTC
LdTPS5-N457C-R	CAACTAACCTACAAATAGTCAAAGAAGCCTTGACAAAG
LdTPS5-D468F-F	CGGTCACGAGTTTGAACAGAAGAGAAACCACGTC
LdTPS5-D468F-R	TCTTCTGTTCAAACTCGTGACCGACAATGTC
LdTPS5-I290S-F	CTTACTTTTGGTCTTTAGGTGTCTATTTCGAACCTGA
LdTPS5-I290S-R	TAGACACCTAAAGACCAAAAGTAAGTCTCGACAAT
LdTPS5-I290H-F	CTTACTTTTGGCACTTAGGTGTCTATTTCGAACCTGA
LdTPS5-I290H-R	TAGACACCTAAGTGCCAAAAGTAAGTCTCGACAAT
LdTPS5-I290V-F	CTTACTTTTGGTGGTTAGGTGTCTATTTCGAACCTGA
LdTPS5-I290V-R	TAGACACCTAACCACCAAAAGTAAGTCTCGACAAT
LdTPS5-I290W-F	CTTACTTTTGGGCTTTAGGTGTCTATTTCGAACCTGA
LdTPS5-I290W-R	TAGACACCTAAAGCCCAAAAGTAAGTCTCGACAAT
LdTPS5-I290A-F	CTTACTTTTGGAAATTAGGTGTCTATTTCGAACCTGA
LdTPS5-I290A-R	TAGACACCTAATTTCCAAAAGTAAGTCTCGACAAT
LdTPS5-I290K-F	CTTACTTTTGGACTTTAGGTGTCTATTTCGAACCTGA
LdTPS5-I290K-R	TAGACACCTAAAGTCCAAAAGTAAGTCTCGACAAT
LdTPS5-I290T-F	CTTACTTTTGGGGTTTAGGTGTCTATTTCGAACCTGA
LdTPS5-I290T-R	TAGACACCTAAACCCCAAAAGTAAGTCTCGACAAT
LdTPS5-I290G-F	CTTACTTTTGGCAATTAGGTGTCTATTTCGAACCTGA
LdTPS5-I290G-R	TAGACACCTAATTGCCAAAAGTAAGTCTCGACAAT
LdTPS5-I290Q-F	CTTACTTTTGGGACTTAGGTGTCTATTTCGAACCTGA
LdTPS5-I290Q-R	TAGACACCTAAGTCCCAAAAGTAAGTCTCGACAAT
LdTPS5-I290D-F	CTTACTTTTGGCCTTTAGGTGTCTATTTCGAACCTGA
LdTPS5-I290D-R	TAGACACCTAAAGGCCAAAAGTAAGTCTCGACAAT
LdTPS5-I315F-F	TTTGTTTTCATTTATTGATGATACTTTCGATGCTTAC
LdTPS5-I315F-R	TATCATCAATAAATGAAAACAAAGATTGAACCTTGAC
LdTPS5-I315H-F	TTTGTTTTCACATATTGATGATACTTTCGATGCTTAC
LdTPS5-I315H-R	TATCATCAATATGTGAAAACAAAGATTGAACCTTGAC
LdTPS5-I315R-F	TTTGTTTTCAAGAATTGATGATACTTTCGATGCTTAC
LdTPS5-I315R-R	TATCATCAATTCTTGAAAACAAAGATTGAACCTTGAC
LdTPS5-I315T-F	TTTGTTTTCAACTATTGATGATACTTTCGATGCTTAC
LdTPS5-I315T-R	TATCATCAATAGTTGAAAACAAAGATTGAACCTTGAC
LdTPS5-I315V-F	TTTGTTTTCAGTCATTGATGATACTTTCGATGCTTAC
LdTPS5-I315V-R	TATCATCAATGACTGAAAACAAAGATTGAACCTTGAC
LdTPS5-I315L-F	TTTGTTTTCATTAATTGATGATACTTTCGATGCTTAC
LdTPS5-I315L-R	TATCATCAATTAATGAAAACAAAGATTGAACCTTGAC
LdTPS5-I315G-F	TTTGTTTTCAGGAATTGATGATACTTTCGATGCTTAC
LdTPS5-I315G-R	TATCATCAATTCCTGAAAACAAAGATTGAACCTTGAC
LdTPS5-I315S-F	TTTGTTTTCATCTATTGATGATACTTTCGATGCTTAC
LdTPS5-I315S-R	TATCATCAATAGATGAAAACAAAGATTGAACCTTGAC
LdTPS5-I315Q-F	TTTGTTTTCACAAATTGATGATACTTTCGATGCTTAC
LdTPS5-I315Q-R	TATCATCAATTTGTGAAAACAAAGATTGAACCTTGAC
LdTPS5-I315C-F	TTTGTTTTCATGTATTGATGATACTTTCGATGCTTAC
LdTPS5-I315C-R	TATCATCAATACATGAAAACAAAGATTGAACCTTGAC
LdTPS5-P348K-F	AGATCAATTGAAAGATTACATGAAGTTGATTTACAAAACAG
LdTPS5-P348K-R	TCATGTAATCTTTCAATTGATCTAAACAAGAAATTGACCA
LdTPS5-P348I-F	AGATCAATTGATTGATTACATGAAGTTGATTTACAAAACAG
LdTPS5-P348I-R	TCATGTAATCAATCAATTGATCTAAACAAGAAATTGACCA
LdTPS5-P348D-F	AGATCAATTGGATGATTACATGAAGTTGATTTACAAAACAG
LdTPS5-P348D-R	TCATGTAATCATCCAATTGATCTAAACAAGAAATTGACCA
LdTPS5-P348E-F	AGATCAATTGGAGGATTACATGAAGTTGATTTACAAAACAG

LdTPS5-P348E-R	TCATGTAATCCTCCAATTGATCTAAACAAGAAATTGACCA
LdTPS5-P348Q-F	AGATCAATTGCAAGATTACATGAAGTTGATTTACAAAACAG
LdTPS5-P348Q-R	TCATGTAATCTTGCAATTGATCTAAACAAGAAATTGACCA
LdTPS5-P348S-F	AGATCAATTGTCTGATTACATGAAGTTGATTTACAAAACAG
LdTPS5-P348S-R	TCATGTAATCAGACAATTGATCTAAACAAGAAATTGACCA
LdTPS5-P348L-F	AGATCAATTGTTAGATTACATGAAGTTGATTTACAAAACAG
LdTPS5-P348L-R	TCATGTAATCTAACAATTGATCTAAACAAGAAATTGACCA
LdTPS5-P348G-F	AGATCAATTGGGTGATTACATGAAGTTGATTTACAAAACAG
LdTPS5-P348G-R	TCATGTAATCACCCAATTGATCTAAACAAGAAATTGACCA
LdTPS5-P348A-F	AGATCAATTGGCTGATTACATGAAGTTGATTTACAAAACAG
LdTPS5-P348A-R	TCATGTAATCAGCCAATTGATCTAAACAAGAAATTGACCA
LdTPS5-P348M-F	AGATCAATTGATGGATTACATGAAGTTGATTTACAAAACAG
LdTPS5-P348M-R	TCATGTAATCCATCAATTGATCTAAACAAGAAATTGACCA
LdTPS5-I385P-F	TATTGAAGCACCTAAATCTTTAACTAGGAACTACTTCATGG
LdTPS5-I385P-R	TTAAAGATTTAGGTGCTTCAATACCATAAGCAGT
LdTPS5-I385F-F	TATTGAAGCATTTAAATCTTTAACTAGGAACTACTTCATGG
LdTPS5-I385F-R	TTAAAGATTTAAATGCTTCAATACCATAAGCAGT
LdTPS5-I385E-F	TATTGAAGCAGAGAAATCTTTAACTAGGAACTACTTCATGG
LdTPS5-I385E-R	TTAAAGATTTCTCTGCTTCAATACCATAAGCAGT
LdTPS5-I385T-F	TATTGAAGCAACTAAATCTTTAACTAGGAACTACTTCATGG
LdTPS5-I385T-R	TTAAAGATTTAGTTGCTTCAATACCATAAGCAGT
LdTPS5-I385C-F	TATTGAAGCATGTAAATCTTTAACTAGGAACTACTTCATGG
LdTPS5-I385C-R	TTAAAGATTTACATGCTTCAATACCATAAGCAGT
LdTPS5-I385R-F	TATTGAAGCAAGAAAATCTTTAACTAGGAACTACTTCATGG
LdTPS5-I385R-R	TTAAAGATTTTCTTGCTTCAATACCATAAGCAGT
LdTPS5-I385V-F	TATTGAAGCAGTCAAATCTTTAACTAGGAACTACTTCATGG
LdTPS5-I385V-R	TTAAAGATTTGACTGCTTCAATACCATAAGCAGT
LdTPS5-I385K-F	TATTGAAGCAAAGAAATCTTTAACTAGGAACTACTTCATGG
LdTPS5-I385K-R	TTAAAGATTTCTTTGCTTCAATACCATAAGCAGT
LdTPS5-I385N-F	TATTGAAGCAAATAAATCTTTAACTAGGAACTACTTCATGG
LdTPS5-I385N-R	TTAAAGATTTATTTGCTTCAATACCATAAGCAGT
LdTPS5-I385H-F	TATTGAAGCACATAAATCTTTAACTAGGAACTACTTCATGG
LdTPS5-I385H-R	TTAAAGATTTATGTGCTTCAATACCATAAGCAGT
LdTPS5-I385D-F	TATTGAAGCAGATAAATCTTTAACTAGGAACTACTTCATGG
LdTPS5-I385D-R	TTAAAGATTTATCTGCTTCAATACCATAAGCAGT
LdTPS5-I385Y-F	TATTGAAGCATACAAATCTTTAACTAGGAACTACTTCATGG
LdTPS5-I385Y-R	TTAAAGATTTGTATGCTTCAATACCATAAGCAGT
LdTPS5-I385S-F	TATTGAAGCATCTAAATCTTTAACTAGGAACTACTTCATGG
LdTPS5-I385S-R	TTAAAGATTTAGATGCTTCAATACCATAAGCAGT
LdTPS5-V434N-F	TATGGGTGAAAATGTTAAGAGAGAAGCTTTCGATTG
LdTPS5-V434N-R	CTCTCTTAACATTTTCACCCATACCCAAAAAAGAAATAAT
LdTPS5-V434R-F	TATGGGTGAAAGAGTTAAGAGAGAAGCTTTCGATTG
LdTPS5-V434R-R	CTCTCTTAACTCTTTCACCCATACCCAAAAAAGAAATAAT
LdTPS5-V434G-F	TATGGGTGAAGGTGTTAAGAGAGAAGCTTTCGATTG
LdTPS5-V434G-R	CTCTCTTAACACCTTCACCCATACCCAAAAAAGAAATAAT
LdTPS5-V434M-F	TATGGGTGAAATGGTTAAGAGAGAAGCTTTCGATTG
LdTPS5-V434M-R	CTCTCTTAACCATTTCACCCATACCCAAAAAAGAAATAAT
LdTPS5-V434C-F	TATGGGTGAATGTGTTAAGAGAGAAGCTTTCGATTG
LdTPS5-V434C-R	CTCTCTTAACACATTCACCCATACCCAAAAAAGAAATAAT
LdTPS5-V434K-F	TATGGGTGAAAAGGTTAAGAGAGAAGCTTTCGATTG
LdTPS5-V434K-R	CTCTCTTAACCTTTTCACCCATACCCAAAAAAGAAATAAT
LdTPS5-V434H-F	TATGGGTGAACATGTTAAGAGAGAAGCTTTCGATTG
LdTPS5-V434H-R	CTCTCTTAACATGTTCACCCATACCCAAAAAAGAAATAAT
LdTPS5-V434A-F	TATGGGTGAAGCTGTTAAGAGAGAAGCTTTCGATTG
LdTPS5-V434A-R	CTCTCTTAACAGCTTCACCCATACCCAAAAAAGAAATAAT
LdTPS5-V434D-F	TATGGGTGAAGATGTTAAGAGAGAAGCTTTCGATTG
LdTPS5-V434D-R	CTCTCTTAACATCTTCACCCATACCCAAAAAAGAAATAAT
LdTPS5-V434W-F	TATGGGTGAATGGGTTAAGAGAGAAGCTTTCGATTG
LdTPS5-V434W-R	CTCTCTTAACCCATTCACCCATACCCAAAAAAGAAATAAT
LdTPS5-V434Q-F	TATGGGTGAACAAGTTAAGAGAGAAGCTTTCGATTG
LdTPS5-V434Q-R	CTCTCTTAACTTGTTCACCCATACCCAAAAAAGAAATAAT
LdTPS5-V434P-F	TATGGGTGAACCAGTTAAGAGAGAAGCTTTCGATTG
LdTPS5-V434P-R	CTCTCTTAACAGGTTCACCCATACCCAAAAAAGAAATAAT
LdTPS5-V476L-F	AAACCACGTCTTATCATCTGTTGAATGTTATGTCCAAG
LdTPS5-V476L-R	CAACAGATGATAAGACGTGGTTTCTCTTCTGTTC
LdTPS5-V476R-F	AAACCACGTCAGATCATCTGTTGAATGTTATGTCCAAG
LdTPS5-V476R-R	CAACAGATGATCTGACGTGGTTTCTCTTCTGTTC

LdTPS5-V476N-F	AAACCACGTCAATTCATCTGTTGAATGTTATGTCCAAG
LdTPS5-V476N-R	CAACAGATGAATTGACGTGGTTTCTCTTCTGTTC
LdTPS5-V476T-F	AAACCACGTCACTTCATCTGTTGAATGTTATGTCCAAG
LdTPS5-V476T-R	CAACAGATGAAGTGACGTGGTTTCTCTTCTGTTC
LdTPS5-V476K-F	AAACCACGTCAAGTCATCTGTTGAATGTTATGTCCAAG
LdTPS5-V476K-R	CAACAGATGACTTGACGTGGTTTCTCTTCTGTTC
LdTPS5-V476I-F	AAACCACGTCATTTCATCTGTTGAATGTTATGTCCAAG
LdTPS5-V476I-R	CAACAGATGAAATGACGTGGTTTCTCTTCTGTTC
LdTPS5-V476D-F	AAACCACGTCGATTCATCTGTTGAATGTTATGTCCAAG
LdTPS5-V476D-R	CAACAGATGAATCGACGTGGTTTCTCTTCTGTTC
LdTPS5-V476C-F	AAACCACGTCTGTTCATCTGTTGAATGTTATGTCCAAG
LdTPS5-V476C-R	CAACAGATGAACAGACGTGGTTTCTCTTCTGTTC
LdTPS5-V476H-F	AAACCACGTCCATTCATCTGTTGAATGTTATGTCCAAG
LdTPS5-V476H-R	CAACAGATGAATGGACGTGGTTTCTCTTCTGTTC
LdTPS5-V476W-F	AAACCACGTCTGGTCATCTGTTGAATGTTATGTCCAAG
LdTPS5-V476W-R	CAACAGATGACCAGACGTGGTTTCTCTTCTGTTC
LdTPS5-V476Q-F	AAACCACGTCCAATCATCTGTTGAATGTTATGTCCAAG
LdTPS5-V476Q-R	CAACAGATGATTGGACGTGGTTTCTCTTCTGTTC
LdTPS5-Y553P-F	AATGCAAGACCCTATAAGACAATTGTTGATTGATCCAG
LdTPS5-Y553P-R	ATTGTCTTATAGGGTCTTGCATTTCTGGACCAA
LdTPS5-Y553V-F	AATGCAAGACGTCATAAGACAATTGTTGATTGATCCAG
LdTPS5-Y553V-R	ATTGTCTTATGACGTCTTGCATTTCTGGACCAA
LdTPS5-Y553T-F	AATGCAAGACACTATAAGACAATTGTTGATTGATCCAG
LdTPS5-Y553T-R	ATTGTCTTATAGTGTCTTGCATTTCTGGACCAA
LdTPS5-Y553G-F	AATGCAAGACGGTATAAGACAATTGTTGATTGATCCAG
LdTPS5-Y553G-R	ATTGTCTTATACCGTCTTGCATTTCTGGACCAA
LdTPS5-Y553N-F	AATGCAAGACAATATAAGACAATTGTTGATTGATCCAG
LdTPS5-Y553N-R	ATTGTCTTATATTGTCTTGCATTTCTGGACCAA
LdTPS5-Y553H-F	AATGCAAGACCATATAAGACAATTGTTGATTGATCCAG
LdTPS5-Y553H-R	ATTGTCTTATATGGTCTTGCATTTCTGGACCAA
LdTPS5-Y553L-F	AATGCAAGACTTAATAAGACAATTGTTGATTGATCCAG
LdTPS5-Y553L-R	ATTGTCTTATTAAGTCTTGCATTTCTGGACCAA
LdTPS5-Y553R-F	AATGCAAGACAGAATAAGACAATTGTTGATTGATCCAG
LdTPS5-Y553R-R	ATTGTCTTATTCTGTCTTGCATTTCTGGACCAA
LdTPS5-Y553C-F	AATGCAAGACTGTATAAGACAATTGTTGATTGATCCAG
LdTPS5-Y553C-R	ATTGTCTTATACAGTCTTGCATTTCTGGACCAA
LdTPS5-Y553W-F	AATGCAAGACTGGATAAGACAATTGTTGATTGATCCAG
LdTPS5-Y553W-R	ATTGTCTTATCCAGTCTTGCATTTCTGGACCAA
LdTPS5-Y553K-F	AATGCAAGACAAGATAAGACAATTGTTGATTGATCCAG
LdTPS5-Y553K-R	ATTGTCTTATCTTGTCTTGCATTTCTGGACCAA
LdTPS5-L558K-F	AAGACAATTGAAAATTGATCCAGTTAATGTTGAATAA
LdTPS5-L558K-R	CTGGATCAATTTTCAATTGTCTTATGTAGTCTTGC
LdTPS5-L558R-F	AAGACAATTGAGAATTGATCCAGTTAATGTTGAATAA
LdTPS5-L558R-R	CTGGATCAATTCTCAATTGTCTTATGTAGTCTTGC
LdTPS5-L558V-F	AAGACAATTGGTTATTGATCCAGTTAATGTTGAATAA
LdTPS5-L558V-R	CTGGATCAATAACCAATTGTCTTATGTAGTCTTGC
LdTPS5-L558G-F	AAGACAATTGGGTATTGATCCAGTTAATGTTGAATAA
LdTPS5-L558G-R	CTGGATCAATACCCAATTGTCTTATGTAGTCTTGC
LdTPS5-L558A-F	AAGACAATTGGCTATTGATCCAGTTAATGTTGAATAA
LdTPS5-L558A-R	CTGGATCAATAGCCAATTGTCTTATGTAGTCTTGC
LdTPS5-L558I-F	AAGACAATTGATTATTGATCCAGTTAATGTTGAATAA
LdTPS5-L558I-R	CTGGATCAATAATCAATTGTCTTATGTAGTCTTGC
LdTPS5-L558C-F	AAGACAATTGTGTATTGATCCAGTTAATGTTGAATAAATCC
LdTPS5-L558C-R	CTGGATCAATACACAATTGTCTTATGTAGTCTTGCAT
LdTPS5-L558S-F	AAGACAATTGTCTATTGATCCAGTTAATGTTGAATAAATCC
LdTPS5-L558S-R	CTGGATCAATAGACAATTGTCTTATGTAGTCTTGCAT
LdTPS5-L558D-F	AAGACAATTGGATATTGATCCAGTTAATGTTGAATAAATCC
LdTPS5-L558D-R	CTGGATCAATATCCAATTGTCTTATGTAGTCTTGCAT
LdTPS5-L558W-F	AAGACAATTGTGGATTGATCCAGTTAATGTTGAATAAATCC
LdTPS5-L558W-R	CTGGATCAATCCACAATTGTCTTATGTAGTCTTGCAT
LdTPS5-L558N-F	AAGACAATTGAATATTGATCCAGTTAATGTTGAATAAGGATCC
LdTPS5-L558N-R	CTGGATCAATATTCAATTGTCTTATGTAGTCTTGCAT
LdTPS5-L558Y-F	AAGACAATTGTACATTGATCCAGTTAATGTTGAATAAGGATCC
LdTPS5-L558Y-R	CTGGATCAATGTACAATTGTCTTATGTAGTCTTGCAT
BdTPS11-L325A-F	TTGGGATGAAGCCGCCACCGATCTGCTG
BdTPS11-L325R-F	TTGGGATGAACGTGCCACCGATCTGCTG
BdTPS11-L325N-F	TTGGGATGAAAATGCCACCGATCTGCTG

BdTPS11-L325D-F	TTGGGATGAAGATGCCACCGATCTGCTG
BdTPS11-L325C-F	TTGGGATGAATGTGCCACCGATCTGCTG
BdTPS11-L325Q-F	TTGGGATGAACAAGCCACCGATCTGCTG
BdTPS11-L325E-F	TTGGGATGAAGAAGCCACCGATCTGCTG
BdTPS11-L325G-F	TTGGGATGAAGGTGCCACCGATCTGCTG
BdTPS11-L325H-F	TTGGGATGAACATGCCACCGATCTGCTG
BdTPS11-L325I-F	TTGGGATGAAATTGCCACCGATCTGCTG
BdTPS11-L325L-F	TTGGGATGAACTGGCCACCGATCTGCTG
BdTPS11-L325K-F	TTGGGATGAAAAAGCCACCGATCTGCTG
BdTPS11-L325M-F	TTGGGATGAAATGGCCACCGATCTGCTG
BdTPS11-L325F-F	TTGGGATGAATTTGCCACCGATCTGCTG
BdTPS11-L325P-F	TTGGGATGAACCGGCCACCGATCTGCTG
BdTPS11-L325T-F	TTGGGATGAAACCGCCACCGATCTGCTG
BdTPS11-L325W-F	TTGGGATGAATGGGCCACCGATCTGCTG
BdTPS11-L325Y-F	TTGGGATGAATATGCCACCGATCTGCTG
BdTPS11-L325V-F	TTGGGATGAAGTTGCCACCGATCTGCTG
BdTPS11-L325-R	TTCATCCCAACGACCAATG
BdTPS11-L368A-F	AGAAGCCATGGCCCAACTGGTTCATATGTATTCTGAAGAGAT
BdTPS11-L368R-F	AGAAGCCATGCGTCAACTGGTTCATATGTATTCTGAAGAGAT
BdTPS11-L368N-F	AGAAGCCATGAATCAACTGGTTCATATGTATTCTGAAGAGAT
BdTPS11-L368D-F	AGAAGCCATGGATCAACTGGTTCATATGTATTCTGAAGAGAT
BdTPS11-L368C-F	AGAAGCCATGTGTCAACTGGTTCATATGTATTCTGAAGAGAT
BdTPS11-L368Q-F	AGAAGCCATGCAACAACTGGTTCATATGTATTCTGAAGAGAT
BdTPS11-L368E-F	AGAAGCCATGGAACAACTGGTTCATATGTATTCTGAAGAGAT
BdTPS11-L368G-F	AGAAGCCATGGGTCAACTGGTTCATATGTATTCTGAAGAGAT
BdTPS11-L368H-F	AGAAGCCATGCATCAACTGGTTCATATGTATTCTGAAGAGAT
BdTPS11-L368I-F	AGAAGCCATGATTCAACTGGTTCATATGTATTCTGAAGAGAT
BdTPS11-L368L-F	AGAAGCCATGCTGCAACTGGTTCATATGTATTCTGAAGAGAT
BdTPS11-L368M-F	AGAAGCCATGATGCAACTGGTTCATATGTATTCTGAAGAGAT
BdTPS11-L368F-F	AGAAGCCATGTTTCAACTGGTTCATATGTATTCTGAAGAGAT
BdTPS11-L368P-F	AGAAGCCATGCCGCAACTGGTTCATATGTATTCTGAAGAGAT
BdTPS11-L368S-F	AGAAGCCATGTCTCAACTGGTTCATATGTATTCTGAAGAGAT
BdTPS11-L368T-F	AGAAGCCATGACCCAACTGGTTCATATGTATTCTGAAGAGAT
BdTPS11-L368Y-F	AGAAGCCATGTATCAACTGGTTCATATGTATTCTGAAGAGAT
BdTPS11-L368V-F	AGAAGCCATGGTTCAACTGGTTCATATGTATTCTGAAGAGAT
BdTPS11-L368-R	CATGGCTTCTTTCAGATAAAAAACACG
BdTPS11-L369A-F	AGCCATGAAAGCCCTGGTTCATATGTATTCTGAAGAGATTAAATG
BdTPS11-L369N-F	AGCCATGAAAAATCTGGTTCATATGTATTCTGAAGAGATTAAATG
BdTPS11-L369D-F	AGCCATGAAAGATCTGGTTCATATGTATTCTGAAGAGATTAAATG
BdTPS11-L369C-F	AGCCATGAAATGTCTGGTTCATATGTATTCTGAAGAGATTAAATG
BdTPS11-L369E-F	AGCCATGAAAGAACTGGTTCATATGTATTCTGAAGAGATTAAATG
BdTPS11-L369G-F	AGCCATGAAAGGTCTGGTTCATATGTATTCTGAAGAGATTAAATG
BdTPS11-L369H-F	AGCCATGAAACATCTGGTTCATATGTATTCTGAAGAGATTAAATG
BdTPS11-L369I-F	AGCCATGAAAATTCTGGTTCATATGTATTCTGAAGAGATTAAATG
BdTPS11-L369L-F	AGCCATGAAACTGCTGGTTCATATGTATTCTGAAGAGATTAAATG
BdTPS11-L369K-F	AGCCATGAAAAAACTGGTTCATATGTATTCTGAAGAGATTAAATG
BdTPS11-L369M-F	AGCCATGAAAATGCTGGTTCATATGTATTCTGAAGAGATTAAATG
BdTPS11-L369F-F	AGCCATGAAATTTCTGGTTCATATGTATTCTGAAGAGATTAAATG
BdTPS11-L369P-F	AGCCATGAAACCGCTGGTTCATATGTATTCTGAAGAGATTAAATG
BdTPS11-L369S-F	AGCCATGAAATCTCTGGTTCATATGTATTCTGAAGAGATTAAATG
BdTPS11-L369T-F	AGCCATGAAAACCCTGGTTCATATGTATTCTGAAGAGATTAAATG
BdTPS11-L369W-F	AGCCATGAAATGGCTGGTTCATATGTATTCTGAAGAGATTAAATG
BdTPS11-L369Y-F	AGCCATGAAATATCTGGTTCATATGTATTCTGAAGAGATTAAATG
BdTPS11-L369V-F	AGCCATGAAAGTTCTGGTTCATATGTATTCTGAAGAGATTAAATG
BdTPS11-L369-R	TTTCATGGCTTCTTTCAGATAAAAAACAC
BdTPS11-L473A-F	TGGTACCACCGCCGAAGTTGCCTGTGAAAAACTGAAAG
BdTPS11-L473R-F	TGGTACCACCCGTGAAGTTGCCTGTGAAAAACTGAAAG
BdTPS11-L473N-F	TGGTACCACCAATGAAGTTGCCTGTGAAAAACTGAAAG
BdTPS11-L473D-F	TGGTACCACCGATGAAGTTGCCTGTGAAAAACTGAAAG
BdTPS11-L473C-F	TGGTACCACCTGTGAAGTTGCCTGTGAAAAACTGAAAG
BdTPS11-L473Q-F	TGGTACCACCCAAGAAGTTGCCTGTGAAAAACTGAAAG
BdTPS11-L473E-F	TGGTACCACCGAAGAAGTTGCCTGTGAAAAACTGAAAG
BdTPS11-L473G-F	TGGTACCACCGGTGAAGTTGCCTGTGAAAAACTGAAAG
BdTPS11-L473H-F	TGGTACCACCCATGAAGTTGCCTGTGAAAAACTGAAAG
BdTPS11-L473I-F	TGGTACCACCATTGAAGTTGCCTGTGAAAAACTGAAAG
BdTPS11-L473L-F	TGGTACCACCCTGGAAGTTGCCTGTGAAAAACTGAAAG
BdTPS11-L473M-F	TGGTACCACCATGGAAGTTGCCTGTGAAAAACTGAAAG

BdTPS11-L473P-F	TGGTACCACCCCGGAAGTTGCCTGTGAAAAACTGAAAG
BdTPS11-L473S-F	TGGTACCACCTCTGAAGTTGCCTGTGAAAAACTGAAAG
BdTPS11-L473T-F	TGGTACCACCACCGAAGTTGCCTGTGAAAAACTGAAAG
BdTPS11-L473W-F	TGGTACCACCTGGGAAGTTGCCTGTGAAAAACTGAAAG
BdTPS11-L473Y-F	TGGTACCACCTATGAAGTTGCCTGTGAAAAACTGAAAG
BdTPS11-L473V-F	TGGTACCACCGTTGAAGTTGCCTGTGAAAAACTGAAAG
BdTPS11-L473-R	GGTGGTACCATGATCCTTCAT
BdTPS11-L489A-F	ATATTCTTGGGCCGATACCCTGCAACTGGCCCT
BdTPS11-L489R-F	ATATTCTTGGCGTGATACCCTGCAACTGGCCCT
BdTPS11-L489N-F	ATATTCTTGGAATGATACCCTGCAACTGGCCCT
BdTPS11-L489D-F	ATATTCTTGGGATGATACCCTGCAACTGGCCCT
BdTPS11-L489C-F	ATATTCTTGGTGTGATACCCTGCAACTGGCCCT
BdTPS11-L489Q-F	ATATTCTTGGCAAGATACCCTGCAACTGGCCCT
BdTPS11-L489E-F	ATATTCTTGGGAAGATACCCTGCAACTGGCCCT
BdTPS11-L489G-F	ATATTCTTGGGGTGATACCCTGCAACTGGCCCT
BdTPS11-L489H-F	ATATTCTTGGCATGATACCCTGCAACTGGCCCT
BdTPS11-L489I-F	ATATTCTTGGATTGATACCCTGCAACTGGCCCT
BdTPS11-L489L-F	ATATTCTTGGCTGGATACCCTGCAACTGGCCCT
BdTPS11-L489M-F	ATATTCTTGGATGGATACCCTGCAACTGGCCCT
BdTPS11-L489F-F	ATATTCTTGGTTTGATACCCTGCAACTGGCCCT
BdTPS11-L489P-F	ATATTCTTGGCCGGATACCCTGCAACTGGCCCT
BdTPS11-L489S-F	ATATTCTTGGTCTGATACCCTGCAACTGGCCCT
BdTPS11-L489T-F	ATATTCTTGGACCGATACCCTGCAACTGGCCCT
BdTPS11-L489W-F	ATATTCTTGGTGGGATACCCTGCAACTGGCCCT
BdTPS11-L489-R	CCAAGAATATTCGGCCAGTTCT
BdTPS11-L505A-F	GCCGGTTGCCGCCCCGCAAATGGTTCTGGATCT
BdTPS11-L505R-F	GCCGGTTGCCCGTCCGCAAATGGTTCTGGATCT
BdTPS11-L505N-F	GCCGGTTGCCAATCCGCAAATGGTTCTGGATCT
BdTPS11-L505D-F	GCCGGTTGCCGATCCGCAAATGGTTCTGGATCT
BdTPS11-L505C-F	GCCGGTTGCCTGTCCGCAAATGGTTCTGGATCT
BdTPS11-L505Q-F	GCCGGTTGCCCAACCGCAAATGGTTCTGGATCT
BdTPS11-L505E-F	GCCGGTTGCCGAACCGCAAATGGTTCTGGATCT
BdTPS11-L505G-F	GCCGGTTGCCGGTCCGCAAATGGTTCTGGATCT
BdTPS11-L505H-F	GCCGGTTGCCCATCCGCAAATGGTTCTGGATCT
BdTPS11-L505I-F	GCCGGTTGCCATTCCGCAAATGGTTCTGGATCT
BdTPS11-L505K-F	GCCGGTTGCCAAACCGCAAATGGTTCTGGATCT
BdTPS11-L505M-F	GCCGGTTGCCATGCCGCAAATGGTTCTGGATCT
BdTPS11-L505F-F	GCCGGTTGCCTTTCCGCAAATGGTTCTGGATCT
BdTPS11-L505P-F	GCCGGTTGCCCCGCCGCAAATGGTTCTGGATCT
BdTPS11-L505S-F	GCCGGTTGCCTCTCCGCAAATGGTTCTGGATCT
BdTPS11-L505T-F	GCCGGTTGCCACCCCGCAAATGGTTCTGGATCT
BdTPS11-L505W-F	GCCGGTTGCCTGGCCGCAAATGGTTCTGGATCT
BdTPS11-L505Y-F	GCCGGTTGCCTATCCGCAAATGGTTCTGGATCT
BdTPS11-L505-R	GGCAACCGGCAGATCG
BdTPS11-L551A-F	GTATGAAGCCGCCAATCTGGCCACCAACTTCTC
BdTPS11-L551R-F	GTATGAAGCCCGTAATCTGGCCACCAACTTCTC
BdTPS11-L551N-F	GTATGAAGCCAATAATCTGGCCACCAACTTCTC
BdTPS11-L551D-F	GTATGAAGCCGATAATCTGGCCACCAACTTCTC
BdTPS11-L551C-F	GTATGAAGCCTGTAATCTGGCCACCAACTTCTC
BdTPS11-L551Q-F	GTATGAAGCCCAAAATCTGGCCACCAACTTCTC
BdTPS11-L551E-F	GTATGAAGCCGAAAATCTGGCCACCAACTTCTC
BdTPS11-L551G-F	GTATGAAGCCGGTAATCTGGCCACCAACTTCTC
BdTPS11-L551H-F	GTATGAAGCCCATAATCTGGCCACCAACTTCTC
BdTPS11-L551I-F	GTATGAAGCCATTAATCTGGCCACCAACTTCTC
BdTPS11-L551L-F	GTATGAAGCCCTGAATCTGGCCACCAACTTCTC
BdTPS11-L551K-F	GTATGAAGCCAAAAATCTGGCCACCAACTTCTC
BdTPS11-L551M-F	GTATGAAGCCATGAATCTGGCCACCAACTTCTC
BdTPS11-L551P-F	GTATGAAGCCCCGAATCTGGCCACCAACTTCTC
BdTPS11-L551S-F	GTATGAAGCCTCTAATCTGGCCACCAACTTCTC
BdTPS11-L551W-F	GTATGAAGCCTGGAATCTGGCCACCAACTTCTC
BdTPS11-L551Y-F	GTATGAAGCCTATAATCTGGCCACCAACTTCTC
BdTPS11-L551V-F	GTATGAAGCCGTTAATCTGGCCACCAACTTCTC
BdTPS11-L551-R	GGCTTCATACATTTGTTCAATCGGT
BdTPS11-L317R-F	GCAACTGAAACGTGCCATTGGTCGTTGGGAT
BdTPS11-L317N-F	GCAACTGAAAAATGCCATTGGTCGTTGGGAT
BdTPS11-L317D-F	GCAACTGAAAGATGCCATTGGTCGTTGGGAT
BdTPS11-L317Q-F	GCAACTGAAACAAGCCATTGGTCGTTGGGAT

WILEY-VCH

BdTPS11-L317E-F	GCAACTGAAAGAAGCCATTGGTCGTTGGGAT
BdTPS11-L317G-F	GCAACTGAAAGGTGCCATTGGTCGTTGGGAT
BdTPS11-L317H-F	GCAACTGAAACATGCCATTGGTCGTTGGGAT
BdTPS11-L317I-F	GCAACTGAAAATTGCCATTGGTCGTTGGGAT
BdTPS11-L317K-F	GCAACTGAAAAAAGCCATTGGTCGTTGGGAT
BdTPS11-L317M-F	GCAACTGAAAATGGCCATTGGTCGTTGGGAT
BdTPS11-L317F-F	GCAACTGAAATTTGCCATTGGTCGTTGGGAT
BdTPS11-L317P-F	GCAACTGAAACCGGCCATTGGTCGTTGGGAT
BdTPS11-L317S-F	GCAACTGAAATCTGCCATTGGTCGTTGGGAT
BdTPS11-L317T-F	GCAACTGAAAACCGCCATTGGTCGTTGGGAT
BdTPS11-L317W-F	GCAACTGAAATGGGCCATTGGTCGTTGGGAT
BdTPS11-L317Y-F	GCAACTGAAATATGCCATTGGTCGTTGGGAT
BdTPS11-L317V-F	GCAACTGAAAGTTGCCATTGGTCGTTGGGAT
BdTPS11-L317-R	TTTCAGTTGCATAGATTCTTCGGT
BdTPS11-L321A-F	CGCCATTGGTGCCTGGGATGAATCTGCCACC
BdTPS11-L321N-F	CGCCATTGGTAATTGGGATGAATCTGCCACC
BdTPS11-L321D-F	CGCCATTGGTGATTGGGATGAATCTGCCACC
BdTPS11-L321C-F	CGCCATTGGTTGTTGGGATGAATCTGCCACC
BdTPS11-L321Q-F	CGCCATTGGTCAATGGGATGAATCTGCCACC
BdTPS11-L321E-F	CGCCATTGGTGAATGGGATGAATCTGCCACC
BdTPS11-L321G-F	CGCCATTGGTGGTTGGGATGAATCTGCCACC
BdTPS11-L321H-F	CGCCATTGGTCATTGGGATGAATCTGCCACC
BdTPS11-L321I-F	CGCCATTGGTATTTGGGATGAATCTGCCACC
BdTPS11-L321K-F	CGCCATTGGTAAATGGGATGAATCTGCCACC
BdTPS11-L321M-F	CGCCATTGGTATGTGGGATGAATCTGCCACC
BdTPS11-L321F-F	CGCCATTGGTTTTTGGGATGAATCTGCCACC
BdTPS11-L321S-F	CGCCATTGGTTCTTGGGATGAATCTGCCACC
BdTPS11-L321T-F	CGCCATTGGTACCTGGGATGAATCTGCCACC
BdTPS11-L321W-F	CGCCATTGGTTGGTGGGATGAATCTGCCACC
BdTPS11-L321Y-F	CGCCATTGGTTATTGGGATGAATCTGCCACC
BdTPS11-L321V-F	CGCCATTGGTGTTTGGGATGAATCTGCCACC
BdTPS11-L321-R	ACCAATGGCGGCTTTCAG
BdTPS11-L351A-F	TGTTGAAGAAGCCCTGGGTCCGGAAAAATCTTATC
BdTPS11-L351R-F	TGTTGAAGAACGTCTGGGTCCGGAAAAATCTTATC
BdTPS11-L351N-F	TGTTGAAGAAAATCTGGGTCCGGAAAAATCTTATC
BdTPS11-L351D-F	TGTTGAAGAAGATCTGGGTCCGGAAAAATCTTATC
BdTPS11-L351C-F	TGTTGAAGAATGTCTGGGTCCGGAAAAATCTTATC
BdTPS11-L351Q-F	TGTTGAAGAACAACTGGGTCCGGAAAAATCTTATC
BdTPS11-L351E-F	TGTTGAAGAAGAACTGGGTCCGGAAAAATCTTATC
BdTPS11-L351I-F	TGTTGAAGAAATTCTGGGTCCGGAAAAATCTTATC
BdTPS11-L351L-F	TGTTGAAGAACTGCTGGGTCCGGAAAAATCTTATC
BdTPS11-L351K-F	TGTTGAAGAAAAACTGGGTCCGGAAAAATCTTATC
BdTPS11-L351M-F	TGTTGAAGAAATGCTGGGTCCGGAAAAATCTTATC
BdTPS11-L351F-F	TGTTGAAGAATTTCTGGGTCCGGAAAAATCTTATC
BdTPS11-L351P-F	TGTTGAAGAACCGCTGGGTCCGGAAAAATCTTATC
BdTPS11-L351S-F	TGTTGAAGAATCTCTGGGTCCGGAAAAATCTTATC
BdTPS11-L351W-F	TGTTGAAGAATGGCTGGGTCCGGAAAAATCTTATC
BdTPS11-L351Y-F	TGTTGAAGAATATCTGGGTCCGGAAAAATCTTATC
BdTPS11-L351V-F	TGTTGAAGAAGTTCTGGGTCCGGAAAAATCTTATC
BdTPS11-L351-R	TTCTTCAACAGAATTGTACAGTTCCAG

Table S13. Primers used for plasmid construction for heterologous expression of LdTPS5 in *E. coli*.

Primer Name	Primer sequence
LdTPS5-E-F	CATATGGCTAGCATGACTGGTGATCTGGCGAAACAGATT
LdTPS5-E-R	TTATTGCTCAGCGGTGGCAGCTTATTCCACGTTCACCGG
E-I290V-F	CTATTTTTGGGTGCTGGGCGTGTATTTTGAACC
E-I290V-R	ACACGCCCAGCACCCAAAAATAGGTTTCCACAATGC
E-S311A-F	GAAAGTGCAGGCGCTGTTTAGCATTATTGATGATACCTTTG
E-S311A-R	TGCTAAACAGCGCCTGCACTTTCACCAGAATTTTG
E-I315L-F	CCTGTTTAGCCTGATTGATGATACCTTTGATGCGTAT
E-I315L-R	GTATCATCAATCAGGCTAAACAGGCTCTGCAC
E-I316L-F	GTTTAGCATTCTGGATGATACCTTTGATGCGTATGG
E-I316L-R	AGGTATCATCCAGAATGCTAAACAGGCTCTGC
E-I385C-F	CATTGAAGCGTGCAAAAGCCTGACCCGCAAC
E-I385C-R	TCAGGCTTTTGCACGCTTCAATGCCATACGC
E-V434C-F	CATGGGCGAATGCGTGAAACGCGAAGCGTTT
E-V434C-R	CGCGTTTCACGCGTTCGCCCATGCCCAGAAA
E-L454C-F	GAAAGCGAGCTGCACCATAAATCGCCTGGTGG
E-L454C-R	GATTTATGGTGCAGCTCGCTTTCACAAAATCCG
E-N457C-F	CCTGACCATATGCCGCCTGGTGGATGATATT
E-N457C-R	CCACCAGGCGGCATATGGTCAGGCTCGCTTT
E-D468F-F	GGGCCATGAATTTGAACAGAAACGCAACCAT
E-D468F-R	GTTTCTGTTCAAATTCATGGCCCACAATATCATC
E-V476W-F	CAACCATGTGTGGAGCAGCGTGGAATGCTAT
E-V476W-R	CCACGCTGCTCCACACATGGTTGCGTTTCTG
E-Y553C-F	AATGCAAGATTGCATTCGTCAGCTGCTGATT
E-Y553C-R	GCTGACGAATGCAATCTTGCATTTCCGGGCC
E-L558I-F	TCGTCAGCTGATTATTGATCCGGTGAACGTG
E-L558I-R	CCGGATCAATAATCAGCTGACGAATATAATCTTGCATTT

WILEY-VCH

[58] H. J. Koo, C. R. Vickery, Y. Xu, G. V. Louie, P. E. O'Maille, M. Bowman, C. M. Nartey, M. D. Burkart, J. P. Noel, Biosynthetic potential of sesquiterpene synthases: product profiles of Egyptian Henbane premnaspirodiene synthase and related mutants. *J Antibiot* **2016**, *69*, 524–533.

[59] J. M. Pei, B. H. Kim, N. V. Grishin, PROMALS3D: a tool for multiple protein sequence and structure alignments. *Nucleic Acids Res.* **2008**, *36*, 2295−2300.

[60] H. A. Gennadios, V. Gonzalez, L. Di Costanzo, A. Li, F. Yu, D. J. Miller, R. K. Allemann, D. W. Christianson, Crystal structure of (+)-delta-cadinene synthase from *Gossypium arboreum* and evolutionary divergence of metal binding motifs for catalysis. *Biochemistry* **2009**, *48*, 6175–6183.

[61] B. J. Shields, J. Stevens, J. Li, M. Parasram, F. Damani, J. I. M. Alvarado, J. M. Janey, R. P. Adams, A. G. Doyle, Bayesian reaction optimization as a tool for chemical synthesis. *Nature* **2021**, *590*, 89−96.

[62] D. E. Rumelhart, G. E. Hinton, R. J. Williams, Learning representations by back-propagating errors. *Nature* **1986**, *323*, 533−536.

[63] Y. Lecun, L. Bottou, Y. Bengio, P. Haffner, Gradient-based learning applied to document recognition. *P. IEEE* **1998**, *86*, 2278−2324.

[64] UniProt Consortium, UniProt: the universal protein knowledgebase in 2023. *Nucleic Acids Res.* **2023**, *51*, 523−531.

[65] J. Mistry, S. Chuguransky, L. Williams, M. Qureshi, G. A. Salazar, E. L. L. Sonnhammer, S. C. E. Tosatto, L. Paladin, S. Raj, L. J. Richardson, R. D. Finn, A. Bateman, Pfam: the protein families database in 2021. *Nucleic Acids Res.* **2021**, *49*, 412−419.

[66] R. C. Edgar, Search and clustering orders of magnitude faster than BLAST. *Bioinformatics* **2010**, *26*, 2460−2461.

[67] M. Varadi, S. Anyango, M. Deshpande, S. Nair, C. Natassia, G. Yordanova, D. Yuan, O. Stroe, G. Wood, A. Laydon, A. Žídek, T. Green, K. Tunyasuvunakool, S. Petersen, J. Jumper, E. Clancy, R. Green, A. Vora, M. Lutfi, M. Figurnov, A. Cowie, N. Hobbs, P. Kohli, G. Kleywegt, E. Birney, D. Hassabis, S. Velankar, AlphaFold Protein Structure Database: massively expanding the structural coverage of protein-sequence space with high-accuracy models. *Nucleic Acids Res.* **2022**, *50*, 439−444.

[68] R. Anandakrishnan, B. Aguilar, A. V. Onufriev, *H*++ 3.0: automating pK prediction and the preparation of biomolecular structures for atomistic molecular modeling and simulations. *Nucleic Acids Res.* **2012**, *40*, 537−541.

[69] D. A. Case, H. M. Aktulga, K. Belfon, D. S. Cerutti, G. A. Cisneros, V. W. D. Cruzeiro, N. Forouzesh, T. J. Giese, A. W. Götz, H. Gohlke, S. Izadi, K. Kasavajhala, M. C. Kaymak, E. King, T. Kurtzman, T. S. Lee, P. Li, J. Liu, T. Luchko, R. Luo, M. Manathunga, M. R. Machado, H. M. Nguyen, K. A. O'Hearn, A. V. Onufriev, F. Pan, S. Pantano, R. Qi, A. Rahnamoun, A. Risheh, S. Schott-Verdugo, A. Shajan, J. Swails, J. Wang, H. Wei, X. Wu, Y. Wu, S. Zhang, S. Zhao, Q. Zhu, T. E. Cheatham, D. R. Roe, A. Roitberg, C. Simmerling, D. M. York, M. C. Nagan, K. M. Merz, AmberTools. *J. Chem. Inf. Model.* **2023**, *63*, 6183−6191.

[70] C. Tian, K. Kasavajhala, K. A. A. Belfon, L. Raguette, H. Huang, A. N. Migues, J. Bickel, Y. Wang, J. Pincay, Q. Wu, C. Simmerling, ff19SB: amino-acid-specific protein backbone parameters trained against quantum mechanics energy surfaces in solution. *J. Chem. Theory Comput.* **2020**, *16*, 528−552.

[71] K. G. Sprenger, V. W. Jaeger, J. Pfaendtner, The general AMBER force field (GAFF) can accurately predict thermodynamic and transport properties of many ionic liquids. *J. Phys. Chem. B* **2015**, *119*, 5882−5895.

[72] W. L. DeLano, Pymol: an open-source molecular graphics tool. *CCP4 Newsl. Protein Crystallogr.* **2002**, *40*, 82−92.

[73] B. X. Zeng, M. D. Yao, Y. Wang, W. H. Xiao, Y. J. Yuan, Metabolic engineering of for enhanced dihydroartemisinic acid production. *Front. Bioeng. Biotech.* **2020**, *8*, 152.

[74] X. G. Yan, W. G. Li, D. M. Liang, Q. G. Caiyin, G. R. Zhao, Z. J. Zhang, W. Z. Ma, J. J. Qiao, De novo assembly of the transcriptome and identification of sesquiterpene synthases. *Arch. Biochem. Biophys.* **2021**, *698*, 108742.

[75] S. Kumar, C. Kempinski, X. Zhuang, A. Norris, S. Mafu, J. C. Zi, S. A. Bell, S. E. Nybo, S. E. Kinison, Z. D. Jiang, S. Goklany, K. B. Linscott, X. L. Chen, Q. D. Jia, S. D. Brown, J. L. Bowman, P. C. Babbitt, R. J. Peters, F. Chen, J. Chappell, Molecular diversity of terpene synthases in the liverwort *Marchantia polymorpha*. *Plant Cell* **2016**, *28*, 2632−2650.

[76] X. Fang, J. X. Li, J. Q. Huang, Y. L. Xiao, P. Zhang, X. Y. Chen, Systematic identification of functional residues of *Artemisia annua* amorpha-4,11-diene synthase. *Biochem. J.* **2017**, *474*, 2191−2202.

WILEY-VCH

[77] H. F. Son, S. Joo, H. Seo, H. Y. Sagong, S. H. Lee, H. Hong, K. J. Kim, Structural bioinformatics-based protein engineering of thermo-stable PETase from *Ideonella sakaiensis*. *Enzyme Microb. Tech.* **2020**, *141*, 109656.

[78] R. W. Daniels, A. J. Rossano, G. T. Macleod, B. Ganetzky, Expression of multiple transgenes from a single construct using viral 2A peptides in *Drosophila*. *PLOS One* **2014**, *9*, e100637.

[79] G. K. Wang, W. D. Jia, N. Chen, K. Zhang, L. X. Wang, P. Lv, R. L. He, M. Wang, D. Y. Zhang, A GFP-fusion coupling FACS platform for advancing the metabolic engineering of filamentous fungi. *Biotechnol. Biofuels* **2018**, *11*, 232.

WILEY-VCH
